# Education level, contraceptive communication, and IUD use among rural-to-urban migrants in China: A qualitative study

**DOI:** 10.1371/journal.pone.0311022

**Published:** 2024-12-23

**Authors:** Danielle Kane, Bing Han, Yu Chung

**Affiliations:** 1 Department of Sociology, Purdue University, West Lafayette, IN, United States of America; 2 Center on Aging and the Life Course, Purdue University, West Lafayette, IN, United States of America; 3 School of Health Sciences, Purdue University, West Lafayette, IN, United States of America; Independent Consultant, UNITED STATES OF AMERICA

## Abstract

Chinese rural-to-urban migrant workers have high rates of unintended pregnancy, yet many are reluctant to choose the most effective forms of contraception, such as IUDs (intrauterine devices). Those who do are often socioeconomically disadvantaged, a finding that contradicts much health research, namely that higher SES individuals can access better healthcare. This puzzle highlights the need to understand better migrant workers’ contraceptive decision-making. This paper reports findings from interviews with 91 migrant workers. Across educational backgrounds, IUD use usually followed contraceptive-related communication. Female interviewees with less education were more likely to have contraception-related discussions, including with doctors, in which the benefits of IUDs emerged. Typically, however, these conversations happened after a pregnancy. The findings suggest a need for public awareness campaigns that destigmatize discussion of contraception and interventions targeted by education level.

## Introduction

Unintended pregnancy is a major health problem for China [1]. According to the Guttmacher Institute [2], from 2015–2019, 58% of all pregnancies were unintended; a study using data from the China Fertility Survey of 2017 found that about 42% of married women had unintended pregnancies [3]. Although to our knowledge there are no national data for China’s rural-to-urban migrant workers, who constitute more than one-third of the total workforce of approximately 876 million [4], they are considered to be even more vulnerable to this problem [5]. More specifically, migrant women are at risk for unintended pregnancy and health problems due to their lower socioeconomic status as migrants and their inferior status as women; moreover, they tend to have less knowledge of and greater unmet need for contraception, when most are of reproductive age [6, 7]. For instance, in a recent study, among married migrants who were pregnant within 24 months after having previously given birth, 8 in 10 pregnancies were unintended [8].

Long-acting reversible contraceptive (LARC) methods, including IUDs (intra-uterine devices) are considered to be among the most effective options for preventing unintended pregnancy [8–10] and have been identified as especially useful for family planning strategies in many countries [10]. This is because they offer many advantages: they have the lowest failure rates among all contraceptive methods [11]; have high continuation and user satisfaction rates [12]; and are significantly more effective than short-acting contraceptives in avoiding unintended pregnancy [13]. Therefore, LARCs, including IUDs, can be a cost-effective public health intervention that, when part of a mix of options, can serve to empower women and hence lower the incidence of unintended pregnancy [14].

China has a long history of IUD use. When family planning policies limited most couples to one child, the government required women to wear an IUD after a first birth. During the 1980s and 1990s, stainless steel rings were used; as many as 60 million women in China were using IUDs by 1988, and about 90% were these rings [15]. Because they had a higher failure and expulsion rate, the State Family Planning Commission decided to in 1993 to stop producing them [16]. Since that time, copper IUDs have become the most widely used form of IUDs in China [16, 17], although levonorgestrel-releasing (hormonal) IUDs have also been available since 2000 [18].

Despite the advantages of LARCs, it is important to recognize that they have been used coercively [14]. Because they require a trained health care provider to implant them successfully, a third party is inherently involved in making the decision to use this form of contraception, which creates an opportunity for coercion [14, 19]. China scholar Susan Greenhalgh [20] notes that while the country’s population size and scarce resources made provider-implemented strategies like IUDs a reasonable approach, “the control aspects of this strategy should not be overlooked.” For instance, access to oral pills–a method that gives women more control–was generally restricted to those who were physically unable to carry an IUD [20].

The approach taken by the government family planning service has changed from requiring IUDs after the birth of one child to a policy of informed choice [19]. High IUD use continued after this policy change, with IUDs being the second most common method of contraception as recently as 2015 [21]. However, some work is finding that recently, both exposure to and use of IUDs is declining for at least some segments of the population, including among migrant workers and women in rural areas (from where migrant workers hail). For instance, a 2009 study found that in rural areas, IUDs were the most popular LARC, and rural women felt that IUDs improved multiple aspects of their quality of life [22]. However, more recent studies found evidence that migrant workers preferred other forms of contraception, especially condoms [11, 19]. Another study of female migrant workers found that condoms were the first choice of contraception regardless of marital status; IUDs did not appear among the top three favored forms [6]. Finally, a study of married migrant workers in Changzhou found that about 55 percent used condoms as compared to the 37 percent who used IUDs [19].

In sum, the effectiveness of IUDs for preventing unintended pregnancy has made them something of a ‘gold standard’ for those concerned about the consequences of this problem, with some scholars even attributing high rates of unintended pregnancy in part to low use of LARCs, including IUDs [23, 24]. One reason for this is that unintended pregnancy is often associated with inconsistent and ineffective contraceptive use, and IUDs remove the need to implement contraception, such as condoms, correctly each time [7, 8, 11, 19, 25]. Moreover, condoms, the most commonly used form of contraception among Chinese migrant workers [7, 8, 19] requires the compliance of male partners, and so are not completely free from the coercion and power dynamics associated with IUDs [26, 27]. The effectiveness of IUDs, together with their relative accessibility, has made them a topic of interest among researchers, some of whom have documented how IUDs are being eclipsed by less effective forms of contraception [8, 11, 19].

Interestingly, in China higher levels of education have been associated with lower rates of IUD use [19]. This finding is surprising because higher education (like other aspects of high socioeconomic status (SES)) has been consistently linked to obtaining the most effective health treatments [28], and there is little reason to expect that the relationship between SES and contraceptive use would not follow this pattern [29]. It also contrasts with other empirical patterns related to disadvantage and contraceptive choice. Globally, lower education is associated with less contraceptive use [30]. For instance, American women with more education were more likely to use IUDs [29, 31]. More specifically, there was a positive relationship between nulliparous women and women who did not intend a future birth, leading the researchers to note the importance of social and financial resources in adopting advanced medical technologies and in overcoming provider bias against IUDs (in the case of nulliparous women) [31]. That the pattern of findings for education and IUD use in China departs from the more commonly found inverse relationship between education and efficacious health treatments warrants further investigation.

One component of the relationship between socioeconomic advantage and efficacious health treatments that has been extensively studied is the role of social capital [28, 32]. While this concept has generated debate in how it is defined, most scholars would accept a definition that includes participation in a social network, and this participation has been found to be found important for health outcomes in China [33]. One aspect of the importance of social capital for positive health outcomes is the potential for these networks to diffuse important health information [34]. This aspect of social capital can be especially important in the Chinese contraceptive context where the inadequacy of reproductive health services has been identified as a factor for widespread misconceptions about IUDs, leading to lack of use [6, 7, 9, 19, 35, 36].

On one hand, social capital has been associated with the utilization of contraceptive services among female migrants [37]. On the other hand, in China contraception is generally considered to be too embarrassing a topic for conversation. Discussing sex-related issues, even with doctors, can be taboo; for instance, the stigmatization of gynecological problems has prevented women from seeing doctors [38]. Rural areas of China, from which migrant workers hail, tend to be more conservative, so this taboo may be even more pronounced [39]. In short, the inadequacy of reproductive health service may make social capital all the more important for contraceptive decision-making, but some evidence suggests that norms against discussing birth control may limit the flow of information normally facilitated by social capital.

Given this context, it may be most instructive to ask who *is* adopting IUDs and explore the path that leads them to this use. Past literature directs our attention to the potential importance of education and of contraceptive-related communication, but the nature of the relationship between the two is unclear. Understanding this relationship, as with contraceptive decision-making more generally, requires uncovering the meanings that individuals assign to contraception [40]. However, the kind of qualitative work that would illuminate these meanings is uncommon for China [41]. This paper uses interview data to investigate how education level and contraceptive-related communication relate to Chinese migrant workers’ use of IUDs.

## Methods

The data for this paper come from interviews with 58 women (32 years old on average) and 33 men (31 years old on average), all of whom were married. Men were included because they are known to influence contraceptive choice but are often excluded from research on reproductive health [42]. Interviewees grew up in Henan province, which is economically under-developed and shares many characteristics with other migrant-sending areas [43]. As the daughter of migrant workers from Henan, the second author used family and acquaintance networks to identify initial interviewees. In addition, she posted invitations for participating in the study on social media, including WeChat and local websites. Additional interviewees were identified through snowball sampling, a method used in research contexts in which especially private topics are discussed [44]. Personal networks and publicizing the study through social media provided two very different seeds, or departure points, for snowball sampling, thus increasing the diversity of perspectives, addressing a main critique of this method [44]. The study protocol and interview guide received approval from the institutional review board of the authors’ university (IRB-2022-697).

The screening of interviewees for eligibility was conducted over the chat app WeChat. China loosened its COVID lock-down policy only in January 2023, making in-person data collection nearly impossible. Data collection took place from July 17th, 2022, to July 14th, 2023. Interviews were conducted by the second author and a research assistant, both of whom are native speakers of Mandarin. The second author also speaks Henanese dialect and, having grown up in Henan, is familiar with the cultural context from which interviewees originated. She reviewed the transcripts of interviews conducted by the Chinese (but not Henanese) research assistant and annotated the transcript in (the few) instances of dialectic or cultural ambiguities.

A guiding strategy of data collection was to include a sufficient number of interviewees in each socioeconomic subgroup that would allow for systematic comparison. To avoid asking participants sensitive, detailed questions about income, we instead asked about their education level, which is correlated both with socioeconomic status and with health outcomes [45]. Table 2 shows the sample size for each education subgroup (that is, those with a high school degree; more than a high school degree; and less than a high school degree) are roughly equal, and we had a target number of 30 for each subgroup. Initially, more interviewees with higher socioeconomic status (SES) showed interest than did people with lower SES, so as recruitment progressed, we prioritized reaching more people from lower educational backgrounds. More specifically, we increased the payment from 100 to 200 yuan (back-tracking to update the payment to those who had already participated). In addition, when asking interviewees to refer to us their friends or colleagues, we emphasized that we were particularly interested in speaking with workers who had a middle school or high school education. Finally we launched an additional round of recruitment on social media.

The data for this study come from a larger study of Chinese migrant workers’ family lives. The interviews were semi-structured and included a set of pre-planned, mostly open-ended questions that allowed for some flexibility in the ordering of questions to accommodate how participants chose to share information and to allow the interview to proceed more organically than would a set of questions that were more rigidly followed. In addition, some questions included probes to elicit more detailed information; in some cases, interviewers also generated probes during the course of the interview that were specific to interviewees’ responses. Probing was especially important for the topic of contraception, for which interviewees sometimes gave shorter or more indirect answers. (For instance, some interviewees referred to condoms as ‘that thing.’) Prior to the section of questions on contraception, interviewees were asked if they would be willing to answer questions related to contraceptive use; three (all with middle school degrees or below) refused, leading to a final sample of 91 respondents.

The full interview guide included questions on marital history (such as how the interviewee met his or her spouse); household dynamics around housework and child care, including the role of parents and in-laws; home ownership; general opinions about marriage; and contraceptive practices and sources of information. For this paper, interviewees were asked about their contraceptive histories; current contraceptive practices; and with whom they discussed contraception. Before the interview, the interviewer introduced the aim of the study and described her background. Interviewees’ written informed consent was obtained before each interview, and they acknowledged that they had the right to pause or stop the interviews at any time, and could refuse to answer any questions. The interviews were conducted through the video function of WeChat, Zoom, or Tencent Meeting, according to the interviewee’s preference. All but two interviews were conducted in Mandarin (two interviewees answered questions in Henan dialects) and lasted about 45–90 minutes, then were transcribed and coded. The interview transcripts can be found in S1 Raw data.

Interviews were analyzed using Deterding and Waters’[46] flexible coding approach, a strategy designed for large interview samples. The first step of flexible coding is data exploration and preparation [46]. At this stage, each of the three authors read the transcripts and used MaxQDA software to create index codes based on responses to interview questions (such as ‘source of contraceptive knowledge’ and ‘current practice’). The authors met to discuss any discrepancies in this coding. Then, the first two authors returned to the material extracted according to the index codes, and generated potential analytic codes. Analytic codes capture material that reflect a focused set of concepts that would form the basis of a single manuscript and are the central component of the second step of flexible coding, data reduction [46]. They emerge through a closer reading of material extracted according to index codes, and identify patterns that emerge in interviewees’ responses that are often unanticipated when the interview guide is drafted. We view analytic codes as a means to increase the resistance of data to preconceived notions, to borrow Tavory and Timmerman’s [47] terms, in an effort to avoid allowing cognitive biases to determine the analysis. The authors then each independently coded a subset of 30 transcripts based on these analytic codes, at which time some additional analytic codes emerged. The authors met again to settle discrepancies in coding of the analytic codes and discuss the merits or shortcomings of the new codes. After this discussion, the second and third authors used MaxQDA to code the remaining transcripts.

The final stage in flexible coding is connecting analytic codes to participant attributes. Based on previous research that found that education level was associated with contraceptive choice [19, 29, 31], the third author used MaxQDA software to code each transcript by education level (high school completion, less than high school, and more than high school). These were then connected to analytic codes so that patterns of responses by education level could be detected. Table 1 shows the index and analytic codes used in this paper, including the sub-theme of each.

**Table 1:** Categories and codes related to contraceptive knowledge and usage.

	Main Themes	Sub-Themes
Index Codes	Sources of contraceptive knowledge	*Friends*
		*Parents*
		*Medical professionals*
		*Internet*
	Change in Contraception	*Switching methods*
		*Discontinuation*
Analytic Codes	Taboo on discussion	*Private*
		*Shame*
	Confusion about method	*Lack of comprehensive education*
		*Inconsistent information*
	Side effects	*Physical health concerns*
	Uncertainty	*Lack of clarity on effectiveness*
		*Misunderstanding of proper use*

Table 2 shows the demographic characteristics of the interviewees. Sixty-four percent were women and were an average of 32 years old. The sample was divided roughly evenly into thirds by education level–that is, by those who attained a high school degree; those with less than a high school degree; and those with more than a high school degree. Interviewees were more likely to be blue-collar workers than white collar workers (34% as compared to 25%). We designated as ‘blue-collar’ occupations such as factory worker, housekeeper, and salesperson. We designated as ‘white-collar’ occupations such as office worker, health care worker, and pharmacist.

**Table 2:** Descriptive statistics (N = 91).

	Freq.	Mean/Prop.
Age		32.08
Marriage Length		7.34
Men	33	.36
Women	58	.64
*Educational Attainment*		
Primary School	6	.07
Middle School	27	.30
High School	31	.34
Vocational College	14	.15
Bachelor’s	7	.08
Master’s	4	.04
PhD	2	.02
*Occupational Status*		
Blue-Collar Workers	31	.34
White-Collar Workers	23	.25
Stay-at-home Mother	24	.26
Self-employed	9	.10
Farmer	1	.01
Student (Hired)	1	.01
Unemployed	2	.02

### Findings

Education level was associated with some differences in contraceptive practice. Table 3 shows the differences in contraceptive methods between interviewees with and without a high school degree. The most noticeable difference was that 45 percent of those with less than a high school degree had used an IUD as compared to only 10 percent of those with a high school degree or more. At the same time, those with less than a high school degree were also more likely to have been sexually active without using contraception (52 percent, as compared to 31 percent of those with at least a high school degree). Most other differences were smaller. The safe period (or rhythm method) was used by 27 percent of interviewees without a high school degree and by 33 percent of interviewees with a high school degree. In addition, more interviewees without a high school degree (27 percent) reported having used emergency pills than did those with a high school degree (19 percent). Other contraceptive practices were similar between the two groups. The rate for people with (27 percent) and without (28 percent) a high school degree using withdrawal was about the same. Sterilization and use of regular (non-emergency) pills were equally rare for both groups.

**Table 3:** Comparisons of contraception method between people with/without A high school degree (N = 91).

	Below High School (n = 33)		High School and Above (n = 58)	
	Freq.	Mean/Prop.	Freq.	Mean/Prop.
Ever Used IUD	15	.45	6	.10
Ever Used Condoms	25	.79	51	.88
Ever Experienced No Contraception	16	.52	18	.31
Ever Used Safe Period	9	.27	19	.33
Ever Had Sterilization	0		1	.02
Ever Had Withdrawal	9	.27	16	.28
Ever Used Emergency Pills	9	.27	11	.19
Ever Used Contraceptive Pills	0		2	.03

### Interviewees with less education were more likely to discuss contraception

Interviewees with less than a high school education were more likely to have had contraceptive-related communications than were those with a high school education or more. For instance, a woman with a primary school education said that a doctor discussed contraception with her when she gave birth to her first child. When asked if she found this helpful, she replied,

“Of course, otherwise I wouldn’t know about it since I just got married. At that time, I was so young, how could I know? After I gave birth to my son, the doctor told me that if …you don’t want a child you can use an intrauterine ring, condoms, contraceptive injections, etc., and I remembered that. Women must protect their own bodies, otherwise who’s going to take good care of them?”

Men were less likely than women to discuss contraception with friends, but when they did, they reported that they found it beneficial. A man with a primary school education did not discuss contraception with family or doctors but noted that he did have these conversations with friends because they “might know something I don’t. By exchanging information, you might learn something new. So I find it useful.”

By contrast, those with at least a high school degree were much more likely to avoid discussing contraception. This was equally true of men and women. The most common reason given for this was that the topic was simply too private–even shameful–to discuss. A junior college graduate said that she rarely discussed birth control with anyone because she “felt too embarrassed to talk about it.” A high school graduate explained, “when it comes to this topic, everyone feels embarrassed. If you’re the kind of person who talks about this, people might look at you differently.” Consistent with her point, in the course of the interview itself, some participants avoided direct reference to the specifics of contraception, referring to condoms, for instance, as “that thing.” A man with a high school degree explained, “you can’t bring this kind of thing to the table. After all, it’s a private matter between a husband and a wife.”

Instead, interviewees with at least a high school degree were much more likely to use the Internet to investigate birth control. When they did, they sometimes found the information confusing; often the biggest impressions they left with was that contraception had harmful side effects. A woman with a high school degree reported that no one had told her about contraception so she looked on the internet and found that birth control pills were not safe. She also noted that “in fact, I really do not understand what the safe period is.” Only later after becoming pregnant did she learn about birth control. A man with an advanced degree, when asked if he knew about forms of birth control besides condoms, replied that he investigated IUDs on the internet; he then asked the interviewer whether she thought IUDs were harmful. He went on to say, “I am not sure whether some contraceptive measures are poisonous, so we haven’t used IUDs…It was out of consideration for her physical health that I didn’t pick these methods.” Similarly, another man believed that the Internet was helpful because it told him about the potential risks of an IUD, so he and his wife opted against using one.

In sum, interviewees with at least a high school degree were much less likely to discuss contraception with others, preferring instead to rely on the internet, which could be a source of misinformation and was also likely to emphasize dramatically the potential for side effects. Men were less likely than women to discuss contraception, relying heavily on the internet, but the same SES pattern was found; that is, men with less education were more likely than their more-educated counterparts to have those conversations.

### IUD use typically came after discussion with others, especially doctors

Most women who adopted IUDs reported that they did so following a discussion about contraception. In some cases, the discussion took place within personal networks. For instance, when asked why she chose an IUD, one woman with a middle school education said that it was recommended by a neighbor, who “said it was better [than condoms].” A woman with a primary school education who had an unintended pregnancy said that she did not want more children because childrearing was very tiring. Her mother told her to get an IUD, so as soon as she recovered from the pregnancy, she went to the hospital to have one fitted.

Other women who used IUDs did so after discussing it with a doctor. A middle school graduate who did not want a third child first went online to check for basic information and then went to the hospital to learn more. She explained, “if you ask someone else [that is, not a doctor], you feel too embarrassed to talk about it. It feels too private..” Another woman with a middle school education who did not want a second child said that she “learned about the IUD from a doctor at the hospital.”

In fact, those interviewees with a high school education or more who had been fitted for an IUD (or whose wives had been) also made the decision to do so following a doctor’s recommendation. Most of the high-school-educated women who had contraceptive-related communications had them in healthcare settings rather than with friends or family. (For instance, among the nine interviewees with a high school degree who reported any contraception discussion, only one had this conversation with family or friends; the remainder spoke to doctors.) In a few cases, this happened in a more formal setting such as a hospital or a local health clinic. A high-school-educated woman who had been fitted for an IUD did so after getting information about it at a local health clinic, where she learned that it was a safe option. She explained that she valued getting this communication “because we wouldn’t really look up this information ourselves. If they promote it, we take notice, but we wouldn’t actively search for it.”

Some interviewees who had not discussed birth control with friends or family suggested they might be willing to do so with a doctor if they could access one. A man with a junior college education, when asked if he discussed contraception with friends or family, replied,

“I haven’t. I couldn’t open my mouth. I feel that this is a very private and secret thing. When I talk to others, I feel as if I have made something private public.” When then asked whether he would consider talking to a doctor or nurse, he responded, “If it’s a doctor and a nurse, I might talk about this, but I haven’t seen a doctor or nurse in this area.” In other words, access to doctors was constrained. In fact, one man with a high school degree, when asked if he had discussed contraception with doctors, responded, “no, my social network is very small, only co-workers and classmates.” That this man seemed to assume that he would only have access to a doctor through a private social connection reveals how uncommon it was for migrant workers to have access to health care providers.

As may already be clear, most conversations about IUDs took place after a pregnancy. For instance, one of the relatively few women with a junior high school education who had an IUD described the hospital-wide program for pregnant mothers in Shenzhen where she learned about this form of contraception. She explained,

“When you are pregnant, you will go to the hospital for the first time, and then there will be a classroom, and then they will tell you everything. If you don’t want to do this, you will go to the gynecology department, and they will give you some contraception…Anyway, the hospital will educate you [about birth control] even if you want the child.

While a few interviewees described being aware of IUDs before marriage because it was a commonly used form of contraception in a particularly rural area, the vast majority of those who had contraception-related discussions did so after pregnancy.

## Discussion

Most interviewees who adopted IUDs did so after discussions with others, and female interviewees with less education were more likely to have these discussions than any other gender-education group. However, these discussions usually took place after pregnancy. By contrast, men and women with at least a high school education were much more likely to find contraception-related discussions too shameful, especially with friends or family. Some of these interviewees indicated a greater willingness to have these conversations with doctors (and those who had IUDs had spoken with doctors), but most relied on the Internet. Based on their reports, however, what they found online was often inaccurate.

The data do not provide a clear answer to why less-educated migrant workers were more likely to have contraception-related communication. Possibly doctors were more likely to initiate discussions of IUDs with less-educated interviewees; for instance, in one study in the US, LARCs were recommended more often to a low SES group than to a high SES group [48]. In China, doctors are sometimes under great time pressures, treating as many as 40 to 70 patients a day [49], which could lead them to initiate these discussions in situations they judged to be the most urgent, as those with lower socioeconomic status have a high rate of premarital pregnancy (which, in the Chinese context, is effectively unintended pregnancy) [25]. Interviews with or ethnographic observations of doctors in the Chinese context could shed additional light on the differential rate of contraceptive conversations.

On the other hand, those with lower education may have been more willing to have these conversations, as they were also more likely than their counterparts to discuss contraception with friends. Although to our knowledge there is no recent work on China regarding education level and willingness to discuss contraception, this explanation would be consistent with Neil Diamant’s [50] finding that after the implementation of the 1950 Marriage Law, rural peasants were more likely than urbanites to attain a divorce in part because of rural peasants’ greater willingness to discuss intimate details of family life in court.

To be clear, wearing an IUD is not in itself a good outcome, if it is not part of an informed choice about contraception. Past research has highlighted the inadequacy of reproductive health education in China, without which, as Zong and colleagues [19] point out, migrant workers cannot make an informed choice. Therefore, a key implication of this study’s findings is the need to improve accessibility of accurate information about all forms of contraception. Given the preference of many to rely on the Internet for contraceptive information, health clinics’ promotion of this information online would be most useful for combatting the confusion some interviewees experienced from the sources they found. Those interviewees who did have contraception-related communications with doctors all reported finding them helpful; for some women, this led them to adopt an IUD. This suggests that the practice of doctors waiting until pregnancy to initiate conversations about contraception, and in particular not offering IUDs to nulliparous women [19, 51], may contribute to high rates of unintended pregnancy among migrant workers. To frame this more positively, that interviewees of all backgrounds were more likely to adopt a LARC after contraceptive-related communication provides concrete evidence for the effectiveness of educational interventions. Among workers who do not have easy access to health care, peer educators could be a cost-effective means to promote informed choice and reduce unintended pregnancy; for this to happen, contraception cannot be treated as a taboo topic. Therefore, there is a need for more general campaigns to reduce the stigma around discussing contraception. Such campaign materials should not be limited to hospitals and could be broadly distributed to smaller local health clinics as well as workplaces. (One interviewee, for instance, found it helpful that condoms were available at her workplace; this could also be a site for providing contraception information).

This study had limitations. As qualitative work the findings are not generalizable to the entire population. Instead, the study aimed to identify a potential mechanism for the finding from survey research that educational disadvantage was associated with a more efficacious medical outcome, a departure from more typical patterns in education and health outcomes [28]. A next step for future research will be testing this mechanism on generalizable samples. In addition, the nature of contraceptive communication with doctors was not examined in a way that systematically distinguished formal presentations from more casual conversations. In future research, it would be useful to know whether it was the doctor or the patient who initiated the conversation. Moreover, it would be helpful to have more details about what specific websites are consulted, as this could aid family planning services in countering misinformation. It would also be useful to identify through survey research other sites for encountering contraceptive information, ranging from schools to more traditional media such as television. Finally, this research focused on the experience of a sample of interviewees who departed from Henan province. Future work should seek a broader slice of the migrant worker population.

## Conclusion

This study investigated how contraceptive communication related to the adoption of IUDs. Women with less than a high school degree were more likely to have these conversations, both with doctors and with family or friends, than were interviewees with more education. Those with a high school degree were more likely to find such discussions embarrassing and instead relied on the internet for contraceptive information, where they sometimes encountered misinformation and extensive discussions of risk that dissuaded them from adopting an IUD. By contrast, in these contraceptive-focused discussions, women with less education learned about their benefits and were more likely to adopt them. However, this usually happened after a pregnancy.

These data reveal that interactions with doctors and nurses can address unmet needs for contraception and suggest that medical personnel and peer health educators be proactive in initiating these conversations. At the same time, public service campaigns that destigmatize birth control as a topic of discussion, both with family and others, could increase the frequency of conversations and hence address unmet contraceptive needs and unintended pregnancy. Tailoring interventions according to education level may also increase their effectiveness for migrant workers.

## Supporting information

S1 Raw data Raw data in English. (DOCX)

S1 Questionnaire Inclusivity questionnaire. (DOCX)
