# Supplementary material for: Education level, contraceptive communication, and IUD use among rural-to-urban migrants in China: A qualitative study
Source: PLoS One. 2024 Dec 23;19(12):e0311022. doi: 10.1371/journal.pone.0311022 (PMC11665986; doi:10.1371/journal.pone.0311022)
Supplement: S1 Raw data — (DOCX) [file pone.0311022.s001.docx]

Interviews were conducted and transcribed in Chinese; a Google translated version is provided to meet the journal’s requirements. Original transcripts are available upon request.

DOL_05:

Role 2: Yes, I understand. My last question is about contraception. Do you think you can answer it easily?
Role 1: Yes, no problem. Role 2: What kind of contraception do you use now? Role 1: We have always used condoms. Role 2: Is it the only way? Role 1: Currently, I have not changed the method. Role 2: Why did you choose this method? Role 1: Because I have never thought of other methods, including no other methods, and I really don’t know much about other methods. From the beginning, I only thought of this one, and then I have always taken this measure. Role 2: I understand. Have you ever had different ideas about this matter? Role 1: I don’t think so. My wife seems to be quite afraid of having children. Role 2: Ok. Okay. Do you talk about contraception with friends? Or family members. Role 1: No. Role 2: Do you talk about related things with doctors or nurses? Role 1: We haven’t really talked to doctors, because using condoms should be a relatively simple and convenient method. Because I'm considering going to the hospital for a pregnancy test recently, and I might talk about this, but I haven't talked to the doctor before. If I talk about this, I think that if the doctor needs to know, I should explain it to them clearly. Role 2: Yes, do you think you have enough knowledge of contraception? Where do you usually get this knowledge? Role 1: I usually search for it on the Internet, and I don't specifically consult relevant people or search for anything on the Internet. The two of us should not be (inaudible 51:01), because we only have this one method, and we have never taken any other methods.

DOL_06:

Role 2: Thank you. This is the last part, and this is about contraception. OK, what form of contraception do you use now?

Role 1: There are the following, one is mainly ejaculation outside the body, and the second is also using condoms, which are probably these two contraceptive methods.

Role 2: Why do you choose these two methods?

Role 1: One is relatively cheap, and the method of use is relatively simple, and it is also relatively mainstream, and everyone also uses this method, probably these two. This is the main one, because other methods, such as taking medicine or going to the hospital for some treatment, are not suitable for us young people, and are also relatively complicated, and the cost is high, so we take the simplest ones.

Role 2: So do you use contraception every time?

Role 1: This is also to look at her period table, the specific ovulation period, but it will account for more than 85%. A high-probability event.

Role 2: Understand. When you discuss contraception, do you always have the same opinion?

Role 1: It's not quite the same, because men sometimes pursue pleasure, which will affect this aspect, but most of the time they will respect women's opinions.

Role 2: Under what circumstances will they not respect? Because you said most of the time.

Role 1: When will they not respect? Because I think about it, for example, if I am happy that day, I will be more impulsive, and sometimes I may not be able to control myself well, which may be a little worse.

Role 2: What will be her reaction and attitude to this?

Role 1: Generally speaking, I will still accept it, but sometimes there will be some contradictions, but generally there will be nothing. Because this is equivalent to the probability I mentioned, about once in 10 times, so if you have seven or eight times a month, it will only happen once or twice in a month and a half or one and a half months. In fact, the probability is relatively low, and it is not said that you will take it very seriously.

Role 2: Understand. Sometimes she may not be so willing to do what you just mentioned. Will you discuss this matter later?

Role 1: We will discuss it, but it is not the main issue, because we have never been pregnant, so it is not a major concern. And if we have a child, it will not affect our current life too much. At most, it will bring the joy forward a little, so we don’t regard it as a whole pressure.

Role 2: Understanding. If she is not very willing to do this, how will she express it to you?

Role 1: That should be direct verbal expression or some rejection in action, probably mainly in two aspects. Generally, there will be no discussion afterwards, because I am also more rational in many cases. In terms of verbal expression, I will pay attention to it. It should be like this. It will not happen very often.

Role 2: Understanding. Do you talk about your contraception-related matters with your friends or family?

Role 1: Not many family members, and friends, think about it, not many either. Because to be honest, there are not many married people around me, that is, there are not many master and doctoral students, not many undergraduates who get married, and many high school students get married, but you are no longer in the same circle, which means that there is less external communication. For example, the classmate opposite you is not married, and if you talk to him about this topic, he may not be very interested.

Role 2: Yes. Will you talk to the doctor or nurse about this?

Role 1: Because I don't always come into contact with doctors and nurses, I haven't consulted with them.

Role 2: Understand. You just mentioned that you are actually mentally prepared and can accept the joy of an unexpected pregnancy. Do you think that because this may happen, you need to consult with the doctor or nurse first?

Role 1: No, this is completely based on Internet searches. I haven't specifically learned about it. Because we have considered that if we do a full set of pregnancy tests, we are more inclined to do a set of these questions after she officially enters the pregnancy and our work is officially stable. Because we are not completely stable yet, we have not fully invested in thinking about these things. At that time, we thought that if there was an accident, we would just let it go. If not, we can just follow the routine.

Role 2: Understand. In addition to the Internet, what other channels do you use to obtain some contraceptive knowledge?

Role 1: Probably not, because I won't go to the library to look up relevant literature. At most, I will communicate with classmates, but I won't communicate much about this because it is a relatively private topic for Chinese people. It is not a topic that is discussed a lot in daily life. Communication should be limited to the Internet.
DOL_07:

Role 2: Yes, how have you been using contraception since you started dating?
Role 1: I used it when we were dating, but after we got engaged, I didn’t care about it that much after a long time, so I just let it go.
Role 2: So you don’t use contraception anymore?
Role 1: No, I didn’t do it well. Role 2: Do you rely more on the safe period, but not really. Role 1: Yes, not very cautious. Role 2: When you were discussing how to use contraception, did you discuss this? How to use contraception? Role 1: I didn’t discuss it much. Role 2: Yes. Role 1: Let it go. Role 2: Have you had different ideas and opinions on contraception in the past four or five years? Because my friend and I sometimes feel that the way of contraception between our partners, whether it is wearing condoms or using the safe period, is sometimes different, and it feels that the things that both parties care about are not particularly consistent. Sometimes boys may not be so careful and think they can get pregnant right away, but for me, I will be very cautious. I think I will be more worried than him. Do you think this will happen to you? Role 1: Is the signal bad on your side or on my side? I didn't hear it clearly. Role 2: That is to say, sometimes girls may be more careful and more afraid of getting pregnant, but boys may not be so worried sometimes. I want to ask if this will happen to you? Role 1: We are about the same. My husband is very worried now. He cares more than me because he has a lot of financial pressure.
Role 2: Yes. Role 1: He is also afraid. Role 2: Will you change your contraceptive method now? Role 1: This will definitely be more careful, and now most people have less frequency after marriage and childbirth, and they will take precautions once in a while. Role 2: Will you use condoms? Role 1: Yes, basically this is the safest. Role 2: Do you plan to have another child? Role 1: No, I already had two before, right? I have a son and a daughter. Role 2: You are so happy. Congratulations. Role 1: Thank you. Role 2: Do you talk to your friends about contraception? Role 1: Yes, I do. Sometimes when I talk to my friends, some of them are not married yet and they don’t have any children. When we talk about this, they say that they must take good contraceptive measures and not have any more accidents. Role 2: I understand. Do you talk about this with your family? Role 1: With your husband? Role 2: For example, my mother, sisters, and father. Role 1: No, why should I talk to them? Role 2: Yes. Role 1: I have to listen to my own wishes. The elderly think that it’s okay to have more children, as long as they can be raised and supported. I will definitely listen to myself. Role 2: Yes, if you can choose again, will you take different contraceptive measures than before? Role 1: I was too young at that time. If I were to choose now, I would definitely plan training well, or just have one child. It’s too tiring to raise a child now.
DOL_08:

Role 1: I understand. If you plan to have a child in two or three years, will you take contraceptive measures now?
Role 2: Yes, I will take contraceptive measures now.
Role 1: Will you always use contraception?
Role 2: Yes, I will definitely wait until two years later. Role 1: Yes,
what kind of contraceptive measures do you generally choose?
Role 2: Condoms, because this is safer now, because some people may postpone their menstrual period or something, I think this method is really not very reliable, if it is reliable, we may have had children a long time ago, because the reason why my wife and I didn’t have a child before, I think a large part of it may be that we calculated the menstrual period, which is not very correct, which is equivalent to my wife going to the hospital for a check-up, checking the follicles, this is the key point, you should pay attention to that.
Role 1: OK.
Role 2: For example, if you want to have a child in the future, you can go to the hospital to check the maturation time of your follicles, to see when your follicles mature, and when they are too mature, the doctor will recommend that you have sex during the mature period, and the chance of getting pregnant will be much greater at this time.
Role 1: I understand.
Role 2: We had been speculating on our own before, according to the time of menstruation, and pushing forward and backward by how many days, but it was not necessarily reliable and might not be scientific. My wife's follicles might mature a few days later.
Role 1: I understood correctly. You actually planned to get pregnant as soon as you got married.
Role 2: Yes.
Role 1: Do you talk to your friends and family about contraception or pregnancy?
Role 2: Yes, my college classmate was also in Beijing before. He was studying for a doctorate at the Chinese Academy of Sciences at the time. He was married. He and his wife should be the same. They wanted to have a child as soon as possible, but they never had one. At that time, my wife was pregnant. We had a chat once, and I asked him, because we used to rent a house together and lived together. Later, because his father-in-law and mother-in-law wanted to come to Beijing for a trip or to see their daughter, we separated later. At that time, we were also chatting, and I asked him, "Is your wife pregnant?" He said no, so I told him the method I just told you, asking your wife to check when the follicles matured. Unexpectedly, they really got pregnant two months later.
Role 1: That's great.
Role 2: He told me he was very grateful when we met once before.
DOL_ 09 :

Role 1: Yes. What kind of contraception do you use now?
Role 2: Contraception, the most primitive way of contraception. Role 1: Condoms, right? Role 2: Yes. Role 1: Is this the only way? Role 2: I think you should ask about the number of times you have sex, right? Role 1: No, I just want to ask whether you use contraception every time? Role 2: Yes, it has become a habit. He can't say that it is simply for contraception. Isn't it relatively hygienic?
Role 1: Yes, yes. Have you ever had different opinions on this matter? Role 2: No. I think my husband is a more responsible person. Because as I said just now, when I gave birth to my son, I had heavy bleeding and was very scared. So every time, even if I told him not to use condoms , he would say no, in case I really got pregnant, it would still be no.
Role 1: Do you talk about contraception with friends, family, doctors, or nurses? Role 2: I won't talk to my friends or anything. I have never talked about contraception, but I will talk about sex.

DOL_011:

Role 1: It's pretty good. What do you usually do since you got married? Contraception, that is, when you don't plan to have children?
Role 2: Anyway, we haven't been together for several years. Role 1: Yes. With your current division of labor in the family, do you have some friends or acquaintances around you? Maybe they are the kind of people who work outside together. What do you think are the advantages and disadvantages of their doing so? Role 2: Yes, there are many people like this around me. They go out to earn money anyway, and the two of them earn more, but there is still the problem of children. The education of children is still not good. Because children nowadays are very good at playing with mobile phones. When they are left at home to go to school, I feel that many children in rural areas play with mobile phones as soon as they come home from school, and they don't do anything else. Housework or anything. This is also a disadvantage. The advantage is that two people are more free when they are together, and they can also earn money.
Role 1: The kind of people you are talking about are the ones whose grandparents take care of the children, right? Role 2: Yes, the grandparents farm at home and take care of the children, and the two go out to work. Role 1: So cute. You think it's because your husband works in the city, and he is watching the couples around him to see what kind of status they have. Will it affect how he gets along with you? Role 2: No, because many of his current jobs are like me or single people who are younger, in their 20s and not married. I don't think he has any. Role 1: I understand. When you and your husband first discussed having children, do you think your parents said they wanted you to have a third child or something? Role 2: No, both parents didn't support it, and it felt too stressful, two boys. Role 1: Yes. If you are working now, what kind of life do you think that kind of life is? Role 2: I think, first of all, it is very free. In addition to work, you can go home. It's that without children around, this and that must be much easier. You can make money and you can go out to play. Better, right? At home, you basically don't go out, it's very boring. Role 1: When you had your first child, did you mention it to him, or did he mention it to you, or did you discuss it? Role 2: No. I just thought that I was old enough. I wanted to have a child when I was 25, so I thought that I would just let it happen naturally. Role 1: So after you got married, both of you were ready, so you basically stopped using contraception and just let him? Role 2: Yes. Role 1: I understand. Do you talk to friends or family about contraception at other times? Role 2: No, never. Role 1: Do you talk to doctors or nurses? Role 2: No. Role 1: Do you think you have enough knowledge in this area? Role 2: Anyway, I don’t have any knowledge in this area. I think that after getting married, the first child will just happen naturally, and the second child will be just one. Two children will be companions and can help each other in the future. I didn’t think much about other things. You mentioned work and so on. I didn’t think about having a child at that time. Role 1: Yes. If you were asked to make a choice now, would you make the same choice as before?

Role 2: Probably not. If it was like before, I would have only had one child. In the current situation, it feels very troublesome. The contradiction is that I have to worry about the child's schooling and going out to work every day. I don't think about that every day.
DOL_012:

Role 1: If you don't plan to have a child now, will you use contraception?
Role 2: We have always used contraception, except for having children. We didn't use contraception in the past. In fact, after this incident, we were rarely together. We didn't feel like going home. Either he wasn't in the mood or I wasn't. The main reason was that I didn't have that kind of thing. He wasn't particularly interested in it. We basically lost it now. If we are relatively tacitly in love now, we will use it on the safest day, if it happens to be a weekend, or within the safest three days, if it happens to be a weekend. Now it's only once. We don't do much contraception on weekdays, and the frequency is also low. Yes, only once a month, basically, based on the current situation.
Role 1: Then you will use safe period behavior contraception, right? Role 2: Yes. Role 1: If it was before, when you just got married, would you use other contraceptive methods? Role 2: When we just got married, she used emergency contraceptive pills, but it wasn't good. He didn't want a child at the time, so I didn't let him take it, and he took it secretly. Later, we used some condoms, but they were rarely used. We didn't use other contraceptives. We didn't use other contraceptives. We used them in vitro, which is more common. Role 1: What happened when she needed to take emergency contraceptive pills? Role 2: Because she didn't like to use condoms. We had just been married for a short time and didn't plan to have children. He also believed that if we wanted to have children, we had to make full preparations and make some adjustments. We didn't prepare for it after we were ready. Then it was a short separation. A short separation is better than a new marriage. I can't calculate the time accurately. I don't remember the time every day. He took the pills during that period. He took it several times during the relationship. I think he took it two or three times. But at that time, I said it was okay. For example, when I told him the number of days of the safe period, he didn't like it. He felt that the safe period was not good, so he took it secretly. The main reason was that he didn't want to have this child. He was not prepared. He didn't want to take any risks. He didn't want any risks. Role 1: Why is he so cautious? Is he so cautious when using contraception? Role 2: She doesn’t like it. I said that there are condoms, but they are all expired. A box can’t be used up in a year. He may have expired. He doesn’t like it. If he doesn’t like it, you, as a boy, can’t control yourself. Sometimes you can’t control yourself. In fact, it’s very rare, very few times, but he doesn’t want to take risks every time. When he doesn’t want to take risks, it’s relatively early, mainly before marriage and when he just got married. Role 1: I understand. Do you talk about this kind of contraception with friends or family? Role 2: No. Role 1: Do you talk to doctors or nurses? Role 2: No, rarely. When you are ready to have a child, you go to the hospital for eugenics and prenatal examination. Then we will talk about it, but we don’t talk much about eugenics. Role 1: Yes, like now if you and your wife may not want a second child for the time being, if your family wants it, it will bring you some pressure. Will their ideas affect your ideas? Role 2: It won’t affect. Because my thoughts are the same as theirs, I want one too, but I can't have it, I can't have it. Role 1: Yes, have you ever thought about having another child, a girl or a boy? That's the preference. Role 2: Either is fine, I don't have a lot of opinions on gender, not a lot. In fact, I feel that the first one is not a girl. Theoretically, you should have a girl and then a boy. In fact, I think girls are pretty good. Role 1: If your economic situation is on the right track later, will you consider having a second child? Role 2: Not only do I want a second child, I would say a third one is fine, because that's human nature. If you look at the United States, if your family has many children, it's a family happiness, and it's good for the children. We, especially those born in the 80s and 90s, especially the 90s generation now, we have suffered from the family planning reform period, the historical reform period, and we are actually a very tragic generation. Many things have been torn apart by this era. I don't think two or three children are too many. Role 1: Yes.
DOL_013:

Role 1: Yes. After you decided not to have children, what kind of contraception methods did you generally use?
Role 2: Condoms, because we haven't had any yet, and we haven't been together for a long time. Role 1: So at other times, for example, when you don't plan to have children, do you also use this method of contraception? Or will you combine it with something else? Role 2: Yes, Role 1: Will you combine it with the safe period? Role 2: It must be combined with the safe period.
Role 1: How did you consider choosing these two methods at that time?
Role 2: I haven't thought about it yet. This question may be discussed further later. Role 1: Do you think there may be other methods? Role 2: Maybe wearing an IUD or something like that. Role 1: In addition to this, will you consider something similar? Is this the only option if you consider it further? Role 2: What else is there? Role 1: For example, men can also get sterilized. Role 2: I haven't considered this. Role 1: IUDs are probably more well-known, and everyone may know more about them. Role 2: This is more common, yes. Role 1: What does your wife think about this? Role 2: She thinks the same as I do. Role 1: I understand. Do you talk to other friends or family about contraception? Role 2: I haven't talked to my family, but I have talked to my friends. Role 1: What do you usually talk about? Role 2: For example, what you just mentioned, what measures to take, etc. Role 1: Do you get some new information from them, or just chat? Role 2: I don't think I have gained any useful knowledge, because after all, I have been working in the medical industry for so many years, so I may know more about this than them. Role 1: So they may have gained more knowledge from you? Role 2: Yes. Role 1: What do you think your knowledge in this field will be improved compared to ordinary people? Role 2: Regarding this knowledge improvement, I don't quite understand what you mean. Role 1: If you didn't work in this industry, what knowledge do you think you might lack now? Role 2: I think the Internet is relatively developed now, and you can search for all kinds of problems on the Internet, so there shouldn't be anything. Role 1: Yes, do you usually go online to learn about it? Role 2: Yes. Role 1: For example, if parents still have expectations for their future children and have their own ideas. Will it affect your future decisions? Role 2: No. Role 1: I understand. Did you plan for both children at that time, or were there any unexpected things? Role 2: We planned it.
DOL_014:

Role 2: Before your second pregnancy, what kind of contraception did you usually take?
Role 1: In vitro.
Role 2: Does it mean ejaculation outside the body?
Role 1: Yes.
Role 2: Is this the only way?
Role 1: And condoms.
Role 2: Do you also consider the safe period?
Role 1: Rarely.
Role 2: What do you think is the approximate rate of condom use? In all these years?
Role 1: Rarely.
Role 2: Why did you choose this method?
Role 1: Condoms are uncomfortable.
Role 2: I understand, so the second accidental pregnancy.
Role 1: The accidental pregnancy was also due to the problem of the safe period.
Role 2: It was because I calculated the safe period, but did not take the in vitro design or condoms, and then I got pregnant.
Role 1: Yes, so the safe period is not safe at all.
Role 2: I have learned a lesson. After this incident, will you and your husband have new ideas about contraception? I just said that after the second pregnancy, will you have new measures for contraception?
Role 1: Currently, we still use in vitro fertilization.
Role 2: Is it mainly he or you who mentioned the discomfort, that is, the discomfort of wearing a condom?
Role 1: Me.
Role 2: I understand. Do you talk to your friends or family about contraception?
Role 1: We talk about specific things. If there is a need, we need to talk to them. We will talk about very little else.
Role 2: Do you consult some doctors or nurses for advice?
Role 1: No.
Role 2: Do you think your knowledge of contraception is enough? Where do you usually learn about this knowledge?
Role 1: I usually see it on some public accounts that do medical promotion, and then I go online to check it out.
DOL_015:

Role 1: What kind of contraceptive methods have you adopted when you don't want to have children?
Role 2: Condoms.
Role 1: Will you combine it with the safe period?
Role 2: Yes. Why?
Role 1: Will you consider using the safe period method?
Role 2: I thought about it before, but she said it was unreliable.
Role 1: Did you have any different opinions when you discussed this matter? When she thought it was unreliable, did you discuss it again?
Role 2: Then I also checked some information and it was indeed unreliable, so I had to accept it.
Role 1: So condoms have always been the only way?
Role 2: Almost.
Role 1: Have you ever learned about, for example, long-acting contraceptive pills, or some contraceptive devices placed in the body, or other forms of contraceptive measures?
Role 2: Contraceptive measures, right? I have learned about them. I also checked online before, and there are contraceptives for men or men. Can men also use contraception? According to my understanding, is it that long-term contraceptive pills are said to be, because I haven't actually studied it in depth, whether long-term contraceptive pills are more harmful to the body. I don’t know if some contraceptive pills are harmless to the body if taken for a long time. So I have never taken such measures. As for the IUD or another kind of IUD, I forgot what it was called. I feel that there may be some sequelae. It may be mainly considered in this aspect. For women, there may be some adverse reactions to their bodies, so I didn’t do anything about it.
Role 1: Yes, do you talk about contraception with your friends and family?
Role 2: I don’t think so.
Role 1: Have you talked about related things with doctors or nurses?
Role 2: Doctors and nurses, I haven’t asked doctors about this. I just looked up information on my own.
DOL_016:

Role 1: What contraceptive methods do you use when you are not considering having children?

Role 2: Basically, condoms are used.

Role 1: Will it be combined with some short-acting contraceptive safety period or other methods?

Role 2: I don't really believe in the safe period, so I might use some short-acting contraceptives like you mentioned during the safe period. Role 1: Is it emergency contraceptives or the short-acting contraceptives that you have to take many times a month?

Role 2: I have to take it many times a month. There is a 30-day cycle and I take it for 21 days, and it seems to stop after 7 days.

Character 1: How did you come up with this approach?

Role 2: Because the contraceptive pills that are taken after sex, such as the 72 emergency ones, are too harmful, so I don't like to take them. I also don't like them very much. Sometimes I would rather use condoms. Maybe in the past, especially before, I felt that the effect might not be very good, so I took monthly pills for a while. People said that injections and IUDs always felt not very good, so we don't trust those very much.

Character 1: Why didn’t you take short-acting contraceptives later?

Role 2: Now that I have a child, I basically spend all my energy taking care of the child, so I have less sex than before. Before, I had sex at least once or twice a week, but now I only have sex once or twice a month, with longer intervals between sexes. So using condoms is relatively more convenient.
Role 1: Do you think it's easy to get short-acting contraceptives where you are?

Character 2: Easy, we have it in every pharmacy here.

Role 1: Is it prescription or over-the-counter?
Role 2: Over-the-counter, just tell him you want birth control pills and he will give them to you.

Role 1: Understand.

Role 2: What does a prescription mean? Is it written by a doctor? No, you don't need a doctor's prescription. You can just go to the pharmacy and get it.

Role 1: Do you think you would talk about contraception with your family, friends, doctors, or nurses?
Role 2: You might have been embarrassed to tell others about it before you had children, but now that you have children and are married, it’s OK to tell others about it. You may have been a little shy before you got married or had children, but now that you have children, you don’t think it’s a big deal.

DOL_017:

Role 1: I understand. So what kind of contraceptive methods did you generally use when you were not considering having children?

Role 2: It’s the safe period.

Character 1: Do you think this is safe?

Role 2: But it seems to be quite safe for me, because we had been in love for about four or five years before we had children, and it has been ten years since we met. We have been together for more than ten years, although we have only been married for three years, and there have been no problems. Only at that time, my daughter was born about three months after we got married. So I also think that the reason why I don’t feel sacrificed is that the child came at the right time. I think it seems that because my menstrual cycle is relatively stable, this may be more accurate for me.

Role 1: I understand. Was it planned when you got pregnant with your daughter, or did it feel like an accident?

Role 2: It was a bit of an unexpected plan, because you said that after dating for so long, we decided to get married. In fact, we were both mentally prepared to become parents. But we didn’t have time to do the checkup before having a baby, so we were actually in a state of being prepared but not completely ready.

Character 1: I see. Is this the only form of birth control you use?

Character 2: No, there is also that one.

Role 1: Condom.

Character 2: What's that called? Yeah.

Role 1: Have you ever considered or used, for example, short-acting contraceptives?

Role 2: I used it once and it affected my menstrual cycle, so we felt that it really had a big impact on my body, so we didn't use it again. We are both considering it now. I said it seems that there is a kind of male called

Role 1: Ligation.

Character 2: It’s not called sterilization, it’s called something like that. Is it sterilization? But isn’t sterilization complete? It seems like there is something that can last for several years.

Role 1: Understand. Understand.

Role 2: I have considered it, but I haven’t understood it in depth yet.

Character 1: So you were using emergency contraceptive pills, the short-acting kind that needs to be taken more than 20 days a month.

Role 2: Urgent.

Role 1: Why did you choose this?

Role 2: Because it wasn’t the safe period at that time, and I was worried because I was not married at that time.

Role 1: I understand. Many people think that short-acting contraceptive pills, which are taken for more than 20 days a month and taken for a long time, are more effective in preventing pregnancy and regulating menstruation. Have you ever looked into them or considered using them?

Role 2: I don’t know. I think it might be because I ate it once and it had a big impact on my body. I might have a feeling of being bitten by a snake and being afraid of wells for ten years.

Character 1: Yes, because.

Character 2: Now that you mention it, I can actually go and learn more about it.

Role 1: Because there may be some confusion in China about emergency contraceptive pills and short-acting contraceptive pills, sometimes they may...

Character 2: Maybe I don’t know enough.

Role 1: I only figured it out later. So you and your husband, from the time you started dating to now, have you ever had any disagreements about which contraceptive method to use?

Character 2: Yeah, sometimes he might not want to bring it, but I will ask for it.

Character 1: Does he listen to you every time you ask him to?

Character 2: Listen, my ultimate move is, I just need to say one sentence, if I get pregnant, it will have a huge impact on my body, then he might wake up.

Role 1: Understand. How do you learn about contraception?

Role 2: I went to Baidu, but I haven’t seen any promotions. Or maybe I just didn’t notice it.
Role 1: Maybe there are not many in China.

Character 2: Because we haven’t watched TV in recent years.

Role 1: Do you talk about contraception with friends, family, doctors, or nurses?

Role 2: Yes, but rarely, really rarely.

Role 1: Understand.

Character 2: I would feel embarrassed
.

Role 1: I especially want to understand how you took contraceptive measures after you first got engaged and before having your first child.

Role 2: The first child.

Character 1: Yes.

Role 2: No action was taken.

Character 1: Do you guys pay attention to the safe period or do you just let nature take its course?

Role 2: No, I don’t think the safe period is safe either.

Character 1: Did you have any concerns about not taking birth control measures at the time? Or were you not worried at all?

Role 2: I guess there was a little bit of it at the beginning, but maybe I was just hoping for the best and thought I’d just take it if I had it.

Role 1: Did you express your initial concerns to your then-fiancé?

Character 2: No.

Role 1: You choose to digest it by yourself. Have you told your friends or family?

Character 2: No, he must have had selfish motives at that time.

Role 1: What do you think his selfish motive is?

Role 2: You can get married after you get pregnant.

Character 1: How did you gradually stop worrying about that?

Role 2: I think since I’m already engaged, I might as well get married. We’re about the same age anyway, so I’ll just let nature take its course.

Role 1: How long after you got engaged did you get pregnant?

Role 2: Three or four months.

Role 1: After you gave birth to your first child, your daughter, did you take any safety measures? Before you gave birth to your second son.
Role 2: I think I did, but I felt like I couldn't last long.

Character 1: Who among you would say that we should stop using contraceptive measures? Was it you who suggested it or him?

Role 2: He.

Character 1: Would you disagree?

Role 2: He might have some concerns. He had just started working at that time, and he was
a little worried if he got pregnant right away. It's not easy to get pregnant anyway. Role 1: Did you tell him to be careful at that time?
Role 2: He said that he didn't want to have a baby so soon, but he still wanted the baby after he had it.
Role 1: Neither he nor you were actually ready to have a second child, but maybe you didn't take good contraceptive measures every time, and then you got the second one. Is that right?

Character 2: Yes.

Role 1: When you take contraceptive measures, do you use condoms?

Role 2: You used condoms at that time because you were planning to have a second child, and it would not be a good idea to do anything else.

Role 1: After giving birth to your second child, what were your contraceptive measures?

Role 2: The ring.

Character 1: How long did it take you to use contraceptive measures?

Role 2: More than a year.

Character 1: How did you decide to use this approach?

Role 2: I think this is safer.

Character 1: Was it you who proposed it or him?

Character 2: I mentioned it, but he doesn’t understand this aspect.

Role 1: Do you think he has this need, so you take this measure, or do you have this need more yourself, so you take this measure?

Role 2: I think this way is safer, that way is not safe, it would be bad if I get pregnant.
Role 1: Did you consider other measures at that time? For example, there are short-acting contraceptive pills.

Role 2: Contraceptive pills cannot be taken frequently, but I have used them before. However, the side effects are too great, and the long-term side effects are too great, so they cannot be used frequently. I still think this one is more reliable.

Character 1: The kind of emergency contraceptive pill you take that you decide to take after the fact.

Role 2: I have also used emergency contraceptive pills.

Role 1: In what situations is it used?
Role 2: It may be that two people have not seen each other for a long time, and then there will be such an emergency situation when they meet.
Role 1: How many times do you remember taking emergency contraceptive pills?
Role 2: Two or three times.
Role 1: Do you think this kind of emergency contraceptive pills and short-acting contraceptive pills are easy to buy?
Role 2: They are easy to buy and can be sold in pharmacies.
Role 1: The short-acting contraceptive pills you understand are the ones that need to be taken for more than 20 days, and then he may answer that you want to take them for a long time. Is there a kind of short-acting contraceptive pill?
Role 2: The kind that is taken once a month.
Role 1: Have you heard of the kind I just mentioned? It will be taken for more than 20 days a month, and then every month, and it may also have the effect of regulating menstruation.
Role 2: I have never come into contact with it.
Role 1: Who do you usually discuss contraception-related matters with?
Role 2: I have never discussed it with others.
Role 1: How do you learn about it?
Role 2: The Internet.
Role 1: Do you think your husband will do his homework on contraception?
Role 2: He shouldn't worry about it, he doesn't know much about it.
Role 1: You will understand, because the IUD is mainly for contraception, but you will understand, for example, there may be the spread of diseases or something, will you take certain contraceptive measures because of this concern?
Role 2: I think there should be no problem for the couple.
DOL_019:
Role 2: Before you decided to have this child, how did you take contraceptive measures?

Role 1: We all use some condoms and birth control pills on the market.
Role 2: Are you taking short-acting birth control pills? The kind that you have to take more than 20 days a month.

Role 1: But we don't take medicine, because it harms the body.
Role 2: So it's mainly condoms, right? Do you consider the safe period or ejaculation outside the body?
Role 1: I think in this case, your safe period is generally not that safe, so I'm more cautious.
Role 2: So I always wear condoms. When you two take this contraceptive measure, have you ever had a disagreement? No.
Role 1: Because he's thinking about my health, and I'm thinking about my own health.
Role 2: Do you know the difference between emergency contraceptive pills and short-acting contraceptive pills?
Role 1: I know. Aren't emergency contraceptive pills more harmful?
Role 2: Do you think short-acting contraceptive pills are easy to buy in China now?

Role 1: It seems that contraceptive pills are all prescribed by hospitals.
Role 2: How do you usually learn about contraception?
Role 1: I usually search online.
Role 2: Do you talk to friends and family about it?
Role 1: Usually not.

Role 2: I talked to the doctor or nurse.
Role 1: I talked to the doctor or nurse during the physical examination.

Role 1: So what methods have you used to prevent pregnancy? When you don't plan to have children.

Role 2: Normal contraception.

Character 1: Condom, huh?

Character 2: Yes.

Role 1: You will also take measures, for example, some people will have a safe period, some people will choose to ejaculate outside the body, and then.

Role 2: No, not at all. Because we are probably under a lot of pressure, and we don’t have this kind of thing, not even once a month.

Role 1: Understanding.

Role 2: Very rarely.

Role 1: Do you ever have disagreements on how to prevent pregnancy?

Character 2: No.

Role 1: When you had your current child, you said you got pregnant before you got married. Was it a planned thing or was it an accident?

Role 2: It’s within my plan. My husband has no plan.

Character 1: Does he know?

Character 2: He doesn't know.

Character 1: Did he know later?

Character 2: He might know, but he won't say it.

Character 1: What were you thinking at that time?

Character 2: I told you, because I want to be with him so much.

Role 1: Understand. Do you usually talk about contraception with your friends or family?

Character 2: No.

Role 1: Will you talk to the doctor or nurse?

Role 2: We haven’t discussed this issue.

Role 1: If you decide not to have children or not to have children for the time being, will you continue to use condoms for contraception? Or will you have other considerations?

Character 2: No, keep going like this.

DOL_021:

Role 1: When you are not considering having children, what methods of contraception do you use?

Role 2: Use a condom.

Role 1: Would you consider other methods? For example, some people would consider safe period, ejaculation outside the vagina, or some contraceptive devices.

Role 2: As for us, I work in pharmaceutical sales, so I have some knowledge about this aspect. Maybe birth control is not very friendly to women, so we don't do it. In addition, for example, if you use ejaculation outside the body to prevent pregnancy, I have a little medical knowledge, and I think this is also very unsafe, so at my request, we still prefer to use the safest and most reliable method for contraception.

Character 1: Did he mention other ways at the beginning?

Role 2: No, I think my husband respects me very much in this regard.

Role 1: I see. If someone around you needs short-acting contraceptives, do you think it would be easy to buy them where you are?

Role 2: This one is very easy to buy.

Role 1: Do you talk to your friends or family about things related to contraception?

Role 2: As for family, we actually rarely talk about this aspect.

Character 1: What about the doctor or the nurse?

Role 2: Doctors or nurses. In fact, we will go to the hospital to come into contact with these doctors and nurses unless we are sick. Under normal circumstances, except for friends around us who may have such friends, we rarely come into contact with them.

DOL_022:

Role 2: Understand. When you were together, what kind of contraceptive measures did you take? Some people would use condoms or calculate the safe period, some would ejaculate outside the vagina, and some might take contraceptive pills or IUDs. Which measures did you generally take?
Role 1: None of us did.

Character 2: Has it never happened?

Character 1: Yes.

Character 2: Why did you decide not to use contraception?

Character 1: Because I think if she gets pregnant we can get married.

Character 2: I understand. And then after you got married?

Role 1: After we got married, we felt that we should have a child. We wanted to have another child, that's it.

Character 2: You didn’t take any contraceptive measures either?

Character 1: Yes.

Role 2: Who of you two do you think took the lead in not taking contraceptive measures?

Character 1: It should be me.

Character 2: Did she ever have a different opinion?

Character 1: Basically no. Basically we want to have another child, and we think it's good to have a child so that we can take care of each other.

Role 2: If she has concerns sometimes, how would she tell you about her concerns?

Role 1: The financial conditions do not allow it for the time being, so I will not consider it for now.

Role 2: If she asks you to take some contraceptive measures, would you agree?

Role 1: I would definitely agree. Why not?

Role 2: Do you talk about contraception with other people, such as friends and family?

Role 1: I think it’s mainly because no one knows much about this. And basically no one talks about it. If you want to talk about this in China, basically, first, no one knows about it. Second, no one talks about it, so it’s rare.

Role 2: Understand. Do you learn this knowledge from the Internet, for example?

Character 1: Ninety-nine percent of it is watched online.

Role 2: Will you consider taking some contraceptive measures in the future?

Character 1: Yes, there are.

DOL_023:

Role 1: You said you got pregnant before you got married. Did you take any contraceptive measures at that time?
Role 2: No.
Role 1: Did you know that you had to take contraceptive measures at that time?
Role 2: Not much.
Role 1: Did you have any concerns at that time? If you didn't take contraceptive measures, would you feel worried?
Role 2: I didn't think about it.

Role 1: What kind of contraceptive measures did you take later?
Role 2: Then I had an IUD inserted.
Role 1: How many years after you got married?
Role 2: I think I had the IUD inserted three or four months after I had my son.

Role 1: How did you make the decision to get an IUD?
Role 2: I don’t want to have any more children.
Role 1: Was it you who said you didn’t want one? Or was it your husband who said it?
Role 2: It was me who said it.
Role 1: Why didn’t you want one at that time?
Role 2: They are only one year apart in age, and I really can’t afford to raise more children.
Role 1: I want to confirm that you mean after your son was born, and then you got an IUD. How did you get pregnant with your son?
Role 2: My daughter was still breastfeeding at the time, and my mother told me that there was no need to take precautions against pregnancy, and that breastfeeding wouldn’t cause pregnancy. I didn’t understand, and we didn’t say anything or worry about anything. Who knew that my daughter was about 6 or 7 months pregnant with my son.

Role 1: So you actually thought about at least waiting, right? But maybe after your mother said that, you thought you didn't need to worry?
Role 2: Yes, I think my mother would understand since she's been through it.
Role 1: I understand. There are other contraceptive measures now. For example, some people use condoms, and some take birth control pills. Do you have other contraceptive methods? Except for IUDs.
Role 2: I know that too. I heard about it from others. Role 1
: Did you consider taking other contraceptive measures at the time?
Role 2: No.
Role 1: Is it because IUDs are more common in your area? Or did your family recommend it or something?
Role 2: This was recommended by someone else. They just said that having an IUD is better.
Role 1: Is it a friend or a family member?
Role 2: A neighbor.
Role 1: If you want to buy birth control pills, do you think they are easy to buy?
Role 2: I don’t know. I haven’t bought any.

Role 1: It sounds like you talk about contraception with your family and friends. What do you usually talk about?
Role 2: This is what a colleague said at work.
Role 1: Do you sometimes talk about contraception with nurses and doctors?
Role 2: No.
Role 1: Do you think that having the IUD installed has any impact on your sex life and your body?
Role 2: After the IUD was installed, there was inflammation, nothing else. Role 1:
How long has this been going on? How long after the IUD was installed?
Role 2: Two years after the IUD was installed.
Role 1: Have you seen a doctor?
Role 2: Yes.
Role 1: What did he say?
Role 2: It means there is inflammation, and then I was given some medicine to take, saying that there is some uterine fibroids, but it is still small now. Role
1: Is it caused by the IUD?
Role 2: I don’t know about this, I don’t know about uterine fibroids, but the serious ones should be caused by the IUD.

DOL_024:

Role 1: When you are not planning to have children, how do you usually prevent pregnancy?

Role 2: Don’t be together during the dangerous period or use condoms.

Character 1: Usually you just look at the safe period, right?

Character 2: Yes.

Role 1: Yes, you may need to talk to your doctor about the side effects, because sometimes the side effects are quite serious. Do you think the safe period is safe?

Role 2: Safe period. I feel it is quite safe for me. I just avoid it during the days of pregnancy. Some people wear condoms when they are about to have their period. I have not been pregnant for such a long time, so I just use these.

Role 1: Your current mindset is that if he gets pregnant, you're ready, right? Psychologically?

Role 2: I am ready, so I will let it happen naturally. If I want it, I will take it. If I don’t want it, I will not force it, but let it happen naturally.

Role 1: I understand. Yes, the safety period is still very risky for some people. They may get pregnant accidentally because it is not so accurate. So some people will use condoms more frequently, basically every time. Some people will take the form of contraceptives, not the emergency contraceptive, but the kind that you may take for more than 20 days a month, which also has the effect of regulating menstruation. Do you think this is true?

Character 2: No.

Role 1: I understand. Have you ever had a disagreement about contraceptive measures?

Role 2: No, because my husband knows my body. When I gave birth to my son, other people had to take injections repeatedly, and they felt no pain. But I couldn’t. The injections didn’t work, and my husband knew that. When my son was over two years old, I went for a checkup and the doctor said that it was okay, that he was completely healed, and that I could have a second child. I told my husband whether we should have a second child, and he said no. I asked why. He said that you don’t know your own body. When I gave birth to my son, I didn’t know it myself. He said that you should wait for two years until your child is older and your body recovers, and then you can have another one. My husband knows how much I suffered from this child. My skin is not very good, and the doctor said that your skin will not recover easily, so we didn’t have one. I wanted one, but my husband said to wait a little longer, wait for more than a year, and he said that if you want one, we will have it, so we never had one.

Role 1: I understand. Would you talk to your family and friends about contraception-related matters?

Character 2: No, I really don’t have that.

Role 1: Where do you generally learn about contraception?

Role 2: Now you can check it online yourself.

Role 1: Have you talked to a doctor or nurse?

Role 2: When I gave birth to my son, the attending physician said this.

Character 1: Do you find this helpful?

Role 2: It must be helpful, otherwise you just got married and didn’t know so much. I felt so young at that time, how could I understand so much? After I gave birth to my son, the doctor told me that if he didn’t want to have a child, he would tell me what contraceptive methods to use. If you don’t want to have a child, he would tell you about methods such as IUD, condoms, and contraceptive injections. I said yes, okay, remember it. A woman’s own body, you have to take good care of it yourself. If you don’t take good care of it, who will take care of you?
DOL_025:

Role 1 : I understand. When you don’t want to have children, what kind of contraceptive methods will you use?
 Role 2 : Basically a safety measure.
 Character 1 : Condom, huh?
 Character 2 : Yes.
 Role 1 : You will consider all these factors. For example, some people may use safe periods or contraceptives or something like that. Will you consider that?
 Role 2 : I basically didn’t consider contraceptive pills.
 Role 1 : Do you avoid pregnancy according to the safe period?
 Role 2 : Also rarely. My wife is my wife. Although she sometimes lets things take their own course with children, she may subconsciously still be quite resistant to what is being asked of her now.
 Character 1 : Do you disagree on contraceptive measures?
 Character 2 : No. It's nothing.
 Role 1 : Yes. How do you usually learn about contraceptive measures? Or things related to contraception?
 Role 2 : I don’t think there is any way. I know about it from hearsay, and I looked it up online to find out the principle, but there is nothing special about it. I didn’t go out of my way to learn about it.
 Role 1 : Will you talk to friends or family?
 Role 2 : No, friends might talk about it, but family members won’t.
 Role 1 : Have you talked to a doctor or nurse?
 Role 2 : I haven’t talked about it, and I don’t know if my wife has talked about it. They should have talked about it at that time, because my wife had an abortion before I had my first child, and it was an accident. They should have talked about those things?
 Role 1 : Why did this accident happen?
 Character 1 : I forgot about it at that time. Anyway, I didn’t plan to have a child. My wife might have said that we should have a lucky mentality. She said at that time that we were outside, not at home. I remember that she said she bought a pill and forgot to take it, so she felt that she shouldn’t be so unlucky. That’s her thought. That kind of thing.
 Role 1 : Did you buy the medicine after the fact or take it as planned?
 Role 2 : Medicine purchased afterwards.
 Role 1 : I understand. Why didn’t you take any safety measures at that time?
 Role 2 : I was not at home at the time, I was outside, so I did not make any special preparations.
 Character 1 : Was it you who proposed it or she?
 Character 2 : I forgot about it. Why would I mention this? It's almost there.
 Role 1 : I understand. What were your thoughts when you decided not to have children? Did you have any disagreements?
 Role 2 : The disagreement was not that big, because we were not married at that time, but were preparing to get married. We were planning to get married, but we hadn’t gotten married yet, and it would be soon. However, I had an idea at that time that was consistent with my parents’ idea that there was no need to fight since we were ready to get married.
 But since we are not married, you can only tell her your ideas and you can’t force her to think. So my parents, because we are not married, you can make suggestions to her, but it is not appropriate for you to interfere with her. At that time, I just let her make her own arrangements.
DOL_026:

Role 1 : What contraceptive measures have you taken since you started dating?
 Character 2 : Do you want to say this too?
 Role 1 : Get a general understanding. For example, most people will use condoms, but some people may use birth control pills or ejaculate outside the body.
 Role 2 : We just have sex outside the vagina because my husband doesn’t like condoms.
 Character 1 : Has it always been like this?
 Character 2 : Yes, it has always been like this, including now that we are married and have not had children in the past few years.
 Role 1 : Did that unexpected pregnancy give you any new ideas about your contraceptive measures? Did you change your mind because of the unexpected pregnancy?
 Character 2 : Yes, I thought it would be scary to get pregnant in this state.
 Character 1 : Why are you still like this after you got married?
 Character 2 : Because he doesn't like it and he doesn't want to wear it.
 Character 1 : So you just accept his attitude, right?
 Character 2 : Yes.
 Role 1 : This should involve some risks. In fact, you have also experienced it. Will the two of you have any unpleasantness due to communication about contraceptive measures?
 Role 2 : I feel that it has been quite safe in the past two years, because I have never wanted a child and I have been taking this measure without any accidents, so I feel relieved. In addition, I now think it would be nice to have a second child. Although I also want to have a second child after preparing for pregnancy, if an accident really happens, I can accept it, so I will still do it this way.
 Character 1 : I see. Would you prepare some birth control pills or something just in case?
 Role 2 : Yes.
 Role 1 : How do you usually learn about contraceptive measures?
 Character 2 : Did you say contraception?
 Character 1 : Yes.
 Character 2 : Because I think that person's common sense is these three points? A birth control pill, a condom, and ejaculation outside the body. If he accidentally ejaculates inside me one day, I will take the birth control pill.
 Role 1 : Emergency contraception.
 Character 2 : Yes.
 Character 1 : How often do you take this medicine?
 Role 2 : Not much, because most of the time I told him to ejaculate outside the body and I asked him to wear a condom, but he always felt uncomfortable after using it and he didn’t want to use it anymore.
 Role 1 : Do you think you feel the same way about wearing condoms as he does? Because some couples say that it is actually the women who feel uncomfortable wearing condoms.
 Role 2 : I think it’s okay. I think it’s okay and it doesn’t have any impact.
 Role 1 : So it mainly depends on your husband's thoughts. Do you understand? Will you consult a doctor or nurse about your current contraceptive method?
 Role 2 : Not consulted.
 Role 1 : Do you think birth control pills are easy to buy where you live?
 Role 2 : It’s easy to buy and available in pharmacies.
 Character 1 : Have you ever considered short-acting contraceptives, not the emergency contraceptives that you might have to take more than 20 days a month?
 Character 2 : I haven't thought about it, and I haven't eaten it
.

Role 2: I understand. When you are not considering having children, what kind of contraceptive measures will you take? Is it condoms? Or other contraceptive measures?
Role 1: (01:01:59) Of course, I am very careful. I try to do it outside the body if possible, and I also pay attention to the safety issues during the safe period.
If it is after marriage, I will actually pay attention to it. The safe period includes these things outside the body. But if you don’t think about it after marriage, you definitely won’t think about it before you have a baby after marriage. It doesn’t matter when you get pregnant. It’s legal. She also said that I want to have a baby, so why would I care about these things. After giving birth to the first child, I will pay attention to it again, because we still don’t want a second child for the time being.
Role 2: I understand how you decided to use these two modes of in vitro and safe period. Role 1
: Actually, I bought condoms before marriage, but both of us felt bad, that is, the experience was extremely bad. Then we think that we haven’t used up all of them until now, so we bought a box and didn’t use it. Maybe both of us think that the experience of this thing is too bad.
Role 2: I understand. Do you consider that there may be accidents with these two methods?
Role 1: Before marriage, I don’t know what my wife thinks. I think before marriage, if you really get pregnant by accident, you should get married quickly.
It doesn’t matter, anyway, the house has been bought. And after marriage, when you get pregnant for the first time, you won’t think about it at all, as long as you don’t pay it back at any time. Now that we are married after the first child, my account is almost settled. In fact, even if there is an accident and I get pregnant with a second child, it is not completely unacceptable. And my wife also said that if the financial pressure is less, we can consider having a second child.
So it doesn’t matter if there is an accident. I have a legal child, so who cares about having a second child.
Role 2: Have you two ever had different opinions on contraceptive methods?
Role 1: No, we have the same opinion. That thing is too bad, and we don’t like it.
Role 2: Do you talk about contraception with friends, family, doctors and nurses?
Role 1: Probably not. She definitely won’t, and basically I won’t either. Very rarely, basically never mentioned, I don't remember it. When I talk with male friends who are more familiar with me, I occasionally discuss this matter, not to say that I can talk about it occasionally, but I will pass it quickly.

DOL_028:

Role 1: The last part of the question is about contraception. If you are not considering having children, what kind of contraception do you usually take? Condoms, safe period, ejaculation outside the body, or contraceptive pills?
Role 2: We would probably choose condoms more often. Role 1
: Why did you choose this contraception method?
Role 2: Because I think this is the safest. We both consider that we must not have children. The pressure will be greater in the past two years. Because now our monthly mortgage is nearly 30,000 yuan. In Zhengzhou, a second-tier city, I think we were stupid back then. Maybe we were too poor before. Suddenly we have some money but don’t know how to spend it. This is also a lesson for us. It’s better to be cash-oriented in the future. Don’t let your life be so bad. Now we dare not have children, because I calculated that our child’s personal expenses are nearly 4,000 to 5,000 yuan per month, which is equivalent to the loan for a house, so we are determined not to have children. After that, there are still the problems mentioned above. I felt that I went through a lot of hardships during the process of giving birth to my first child. Although I am much luckier than those who don't have a mother-in-law or parents to help take care of their children, I feel that this process was too hard and difficult. Especially after the first year of giving birth, your whole mental state and state, I think at that time, you are particularly prone to depression and anger. Thinking about it again, my current financial situation, plus my mother-in-law is now over 60 years old, so if I have another child, she will not be able to help me take care of it. Because in the past two years, I have felt that her physical strength is not as good as before, and she should not be able to take care of children anymore. So if I have another child, I have to adjust my current financial situation before it is possible. But I calculated that day, if I adjust these things, such as selling the house and not paying off the mortgage, if the market economy is better, I can only give birth to another child in three or four years. By that time, I will be 37 or 38 years old, old, and it is very likely that I will not be able to give birth. Let nature take its course. So in this matter, he and I are very cautious. Because I think I am easy to get pregnant, so I am very cautious about this.

Role 1: I understand. Have you ever accidentally gotten pregnant before?
Role 2: No. Because it was very strange at that time, he said he wanted his father to take a look at our child, and we got pregnant that time. I think I am easy to get pregnant, because I can digest everything I eat very well, and I rarely have period, so sometimes we also take the method of external ejaculation.

Role 1: I understand. Have you ever had different opinions on deciding on contraceptive measures?

Role 2: No.

Role 1: It's good.

Role 2: I think we don't have any big differences in our thinking.

Character 1: I think so too, it’s good, this is especially good.

DOL_029:

Role 1: Yes. When you don't plan to have children, what kind of contraception do you usually take?
Role 2: On the one hand, we wear condoms, and on the other hand, we ejaculate outside the body. Role 1
: These two are indeed more common. Why do you choose these two methods?
Role 2: We don't know other methods, and we haven't considered other methods.

Role 1: Some people may consider the safe period or use some contraceptive pills, or place it in the uterus for birth control.
Role 2: I know what you said about this method. My wife may be easy to get pregnant, because I feel that I have to have sex once if I want to have a child. The second accident is also very easy to have sex, and we have not considered things like ovulation. But I feel that she may be easy to get pregnant, and it is easy to have a child. We have not considered the ovulation period, fearing that it is not safe. On the other hand, my wife is particularly opposed to the idea of using an IUD. She doesn't want to, saying that it is not good for girls. I don't force it, it doesn't matter. As for taking medicine, I have never considered it, because I know that those medicines are very harmful to the body, and I have never considered this kind of thing.
Role 1: Have you seen the difference between emergency contraceptive pills and short-acting contraceptive pills? In fact, these two are very different. The one you said may be an emergency contraceptive, and the other is a short-acting contraceptive, which may be taken every day when you don't have your period, and taken for a long time. That is not the same, but I think condoms are also good. Have you ever had unpleasant experiences because of contraceptive methods?
Role 2: No, nothing unpleasant happened.
Role 1: Where do you usually learn about contraception?
 Role 2: We were both not young when we met, almost 30. At that time, each of us probably learned from different channels. I don't know where I learned from, but I learned more from Baidu.
Role 1: Yes. Do you talk to friends, family, doctors, or nurses about related matters? Do they have any suggestions and help?
Role 2: I haven't talked about it. I feel like I can't open my mouth.
Role 1: I understand. Do you think your life would be different if you were not married now?
Role 2: I think marriage is good. If I hadn't been married, I might not work at home, and I might still work outside. Maybe no one would care about me. If I hadn't been married at that time, I think I might have had money to spend, but after getting married, I know how to save money and I might consider things more.
Role 1: Back to the question just now, why do you feel that you can't open your mouth to talk about matters related to interests with others?
Role 2: I feel that this is a very private and confidential matter. When you talk to others, it feels like you are exposing some of your privacy.
Role 1: Even if the other party is a doctor or nurse, who is a professional, will they do so?
Role 2: If it is a doctor or nurse, I have not seen any doctor or nurse in this field. If it is a doctor or nurse, I might talk to him if we talk about this topic, but I have not seen any doctor or nurse in this field, nor have I consulted them.

DOL_030:

Role 1: I understand. You said it was God's will, can I understand that you had an accidental pregnancy?
Role 2: Yes.
Role 1: What methods of contraception did you use at that time, or have you always used? Most people may use condoms, some may use safe periods or contraceptive rings, contraceptive pills, and ejaculation outside the body. What methods do you mainly use?
Role 2: Condoms.
Role 1: Are there any others?
Role 2: Did you have an IUD?
Role 1: Was it after you had three children?
Role 2: I had it for a while after the second one.
Role 1: Why did you decide to take it out?

Role 2: Not used to it, not in good health.
Role 1: She had a reaction, right?
Role 2: Yes.
Role 1: What did the doctor say at that time?
Role 2: The doctor said that I don’t know about her physical condition, I don’t know what he said, you just can’t use it, so you took it out.
Role 1: I understand. How did you decide to get an IUD after having two children?

Role 2: Did you not want it at that time?
Role 1: I understand. When you did not want it, you could use condoms. How did you feel about getting an IUD? Did you find it convenient?

Role 2: Yes.
Role 1: Did she bring it up?
Role 2: We discussed it.
Role 1: Did you bring it up first?
Role 2: Yes.
Role 1: When you had your first child, was it before or after you got married?
Role 2: I got pregnant before I got married. Role
1: How did you feel at that time?
Role 2: Not much, it was a bit unexpected.
Role 1: Yes, did you feel unprepared? Because you said you were about 21 years old at that time, some people may think it's a bit early, some people may not think it's early, because my father and mother gave birth to me when they were 20 years old, it's not certain, what kind of mood did you feel at that time?
Role 2: How should I put it? It was an accident, and I didn't think it through at that time.
Role 1: What did she think at that time?
Role 2: Not much, anyway, I was pregnant, and both parents had met, and they had no objections, so I went back to get married. Role
1: I understand. If you hadn't gotten pregnant at that time, when do you think you would have gotten married?
Role 2: Maybe two years later.
Role 1: I understand. Would you discuss contraception with other people?
Role 2: Not really. I can't discuss this with anyone.
Role 1: Why do you think it's impossible to discuss?
Role 2: How do you put it? There's no one to discuss it with. You can't find the right person to talk to. You can't bring this up on the table? After all, it's a matter between you and your wife.
Role 1: Do you talk about these things with doctors or nurses?
Role 2: No.
Role 1: Does your wife know about it? Because she might be in the hospital, and there might be such an opportunity at any time.
Role 2: No, nothing.
Role 1: I know. After your wife's IUD was removed, what kind of contraception did you use afterwards?
Role 2: Condoms.
Role 1: Are you planning to have a third child?
Role 2: Almost.
Role 1: I know. Have you ever had a different opinion with your wife on the matter of contraception?
Role 2: Yes.
Role 1: Can you tell me about it?
Role 2: How do you put it? She's afraid of getting pregnant, what should I say? Whatever makes you feel comfortable.
Role 1: If there are different opinions, what will you do?
Role 2: Listen to her. Role 1
: Understand. Do you learn about related things from the Internet? Contraception-related.
Role 2: I have seen it.
Role 1: Some people may consider using condoms to prevent diseases or something besides pregnancy. Do you consider things related to diseases and decide how to take contraceptive measures?
Role 2: No.

DOL_031:

Role 1: Yes, I see. So when you had your first child, because it was an accident, did you become more cautious about contraception?
Role 2: Yes, that's right.
Role 1: So before the accidental pregnancy, what kind of contraception did you usually take? Or did you not use contraception?
Role 2: I did use contraception.
Role 1: Yes.
Role 2: But not too often.
Role 1: Well, did you use condoms at that time? Or contraceptive pills, or did you just use the method of ejaculation outside the body?
Role 2: I never used contraceptive pills, I just used condoms or ejaculation outside the body.
Role 1: Yes, I see. After you got married, how did you usually use contraception?
Role 2: After marriage, I usually use condoms.
Role 1: It sounds like you will have more frequent and more careful use of contraception, right?
Role 2: Yes. Yes.
Role 1: Is this because you are worried about accidental pregnancy?
Role 2: Yes, that's right.
Role 1: So what do you think this worry feels like to both of you? Are you worried that having another child will be a heavy burden? Like you said before, or?
Role 2: Yes, I am afraid that if I have another child, my life will definitely be affected in the future, or because if two people, she takes care of the child, it may be more financially, mainly financially, and it will be more stressful.
Role 1: Indeed, some considerations about health and safety will affect your choice of contraceptive measures? For example, some people are more worried about the spread of sexually transmitted diseases, so they will consider different contraceptive measures. Do you think you have ever considered this?
Role 2: No. I think using condoms is still safe.
Role 1: Yes, yes, so you have gone through two stages of choosing contraceptive measures from the beginning to now. During this process, do you think your opinions with your wife have always been the same? Or at what stage did the two of you actually have some disagreements about this matter?
Role 2: No disagreement on this.
Role 1: Yes, I understand. Do you talk to your family about how to use contraception?
Role 2: No.
Role 1: What about friends?
Role 2: I don’t talk to my friends, I rarely talk about this.
Role 1: Do you talk to doctors or nurses?
Role 2: No.
Role 1: Where do you usually get information about contraception?
Role 2: How should I put it? I think I will learn it when I grow up. Role
1: Is it through the Internet or something else?
Role 2: The Internet, mainly the Internet.
DOL_032:

Role 1: I see. Can you ask, what kind of contraceptive method have you adopted all along?
Role 2: I had an IUD inserted before, and later used condoms.
Role 1: Do you adopt methods like safe period, withdrawal, and contraceptive pills?
Role 2: Yes. Not very often, I think.
Role 1: Why?
Role 2: I have never taken that kind of medicine, and I don’t know. It might be too late, and then it will be like that. Role 1:
So it sounds like you are quite careful.
Role 2: Not bad.
Role 1: So when you just got married, you got pregnant unexpectedly. Why do you think you got pregnant unexpectedly?

Role 1: I don't have any knowledge of this. I haven't studied it.

Character 2: Were there any safety measures in place?

Role 2: No.
Role 1: Did you not take any safety precautions during those months of dating?
Role 2: Yes.
Role 1: Did you calculate the safe period or something like that?

Role 2: No.
Role 1: There were no contraceptive measures at all, right?
Role 2: It seemed that there was no such awareness at that time.
Role 1: When you were taking contraceptive measures, did you take certain measures because you were worried about accidental pregnancy?
Role 2: Yes.
Role 1: Do you also have some concerns about sexually transmitted diseases?
Role 2: No, I don’t think I’ve thought about it too much.
Role 1: I understand. Have you and your husband ever had different opinions on how to take contraceptive measures?
Role 2: No.
Role 1: For example, before you got married, you both thought there was no problem with it, so you just did it that way, right?
Role 2: I understand.
Role 1: Do you talk to your family or friends about contraceptive measures?
Role 2: No. How can you talk about such a private matter? It doesn’t matter.
Role 1: Do you talk to others now?
Role 2: No.

Do you talk to doctors and nurses?

Except when I go to see a doctor, he asks me, I might say.
Role 1: Do you learn about contraception or reproductive health from the Internet or books?
Role 2: I have learned about it before, because after I gave birth to my first child, I had a high chance of getting pregnant, so I went to see and researched this.

DOL_033:

Role 2: What was the reason for you to have a child?
Role 1: I didn't plan to have a child, but then I got one for no apparent reason, so I kept it. And then until now, it's actually pretty good.

Role 2: Indeed, did you have your child before or after you got married?
Role 1: A few months before you got married. After we met, we bought a house, and she met her parents. Both parents met, and then they looked for a date, and found a fairy, and chose a date. After looking, they confirmed the date, and a few months before the date was confirmed, we got pregnant.

Role 1: How did you decide to keep the child?
Role 2: Let nature take its course.
Role 1: Yes, I see. So how did you and your current wife use contraception? Role 2: We used condoms. Role 1: How
often do you think you would use condoms?
Role 2: Always.
Role 1: So you used condoms and got pregnant unexpectedly?

Role 2: Not before, I got pregnant during the safe period.
Role 1: I see. Then.
Role 2: Now I am cautious, and I have been using it all the time.
Role 1: I see. Is it because you are more worried about accidental pregnancy now?
Role 2: Yes, now I don’t plan to have a second child, so I went for it. If I get pregnant unexpectedly, I will have surgery, but she has a caesarean section, and if she has surgery, it will hurt her body a lot, right?
Role 1: Will you consider other contraceptive measures in the future?
Role 2: No.
Role 1: OK. Will you use condoms because you are worried about the spread of diseases? Role 2:
I don’t worry about that.
Role 1: You and your wife are here, you say.
Role 2: Because we are both mysophobes, (inaudible 00:
4:20) There are no other problems. What is the third point? We don’t do all these messy things.
Role 1: Yes, I see. Have you and your wife ever had different opinions on what kind of contraception to take since you met? For example, the early safe period, have you two reached a consensus?
Role 2: No, I took the pills at first, the contraceptive pills, but the contraceptive pills can cause menstrual irregularities, and her mood was not very stable, so she didn't take them very often, and she always used condoms.
Role 1: Is it the kind of contraceptive pills that you take every day? Or the emergency contraceptive pills?
Role 2: Emergency.
Role 1: Oh, I see.
Role 2: I took it twice.
Role 1: How did she feel at that time?
Role 2: She was not very happy.
Role 1: Was it because she was not very happy about the fact that she needed to take the emergency contraceptive pills? She also told me that she wanted me to get sterilized, and the side effects seemed to be greater.
Role 2: It was a joke.
Role 1: Where do you usually learn about contraception?
Role 2: It seems that I haven't deliberately learned about it, but these are common sense.
Role 1: Do you learn about it from the Internet?
Role 2: No, I should have seen it before, but I can't remember the details now.
Role 1: Do your family talk to you about how to prevent pregnancy?
Role 2: No.
Role 1: What about friends?
Role 2: No friends either.
Role 1: What about doctors and nurses?
Role 2: No, I don’t have any. My social circle is very small. Apart from my colleagues and classmates, there is not much else.

DOL_034:

Role 1: I see. How have you been using contraceptive measures?
Role 2: I bought condoms.
Role 1: Did you consider safe period ejaculation and other methods, such as emergency contraceptive pills?
Role 2: I have never taken contraceptive pills. Role
1: Have you used the safe period?
Role 2: I used it once in a while.
Role 1: I see. Before marriage, when you had your first accidental pregnancy, what contraceptive method did you use during that period?
Role 2: That seemed to be the safe period, she remembered it wrongly.
Role 1: I see. Later, after you got pregnant with your first child, do you think you changed your contraceptive method?
Role 2: No.
Role 1: Still consider the safe period and use condoms?
Role 2: Yes.
Role 1: Will you use more condoms because you are worried about accidental pregnancy ?
Role 2: Yes.
Role 1: Are you worried about the spread of diseases?
Role 2: Yes, of course.
Role 1: How did you decide to use this contraceptive method?
Role 2: Because I don’t want children.
Role 1: Have you ever had different opinions? In this matter?
Role 2: No.
Role 1: Has she ever asked you to wear a condom because she felt that the safe period or the method of ejaculation outside the vagina was unsafe?
Role 2: Yes, that happened. Role
1: If this happens, how do you usually deal with it?
Role 2: Wear a condom.
Role 1: So you will listen to her.
Role 2: Yes.
Role 1: Where do you usually learn about contraception?
Role 2: I think you know all about contraception, but I don’t say you know it in depth.
Role 1: Do you learn about it from the Internet?
Role 2: I don’t know it in depth, and I didn’t search it online. It’s basically common sense.
Role 1: Have you learned some knowledge about contraception from your family, friends, doctors, or nurses?
Role 2: No, because my family members are studying medicine and don’t know much about it.
Role 1: I understand, so she knows more about it.
Role 2: Yes.
Role 1: I understand. Do you listen to her opinions more on these things? Because it sounds like she may have more knowledge.
Character 2: Yeah. Yes.

DOL_035:

Role 1: Next, your second daughter was born during an unexpected pregnancy. We would like to know whether you and your spouse have considered contraception during the whole process from meeting to getting married and having two children. Role 2: After the second child was
born, we considered contraception.

Role 1: What about before this?
Role 2: I hadn't considered it before. I had this idea before, but I hadn't considered it. I also took measures. I remember buying condoms. I took this measure, but you know, sometimes men and women forget about it when they are excited.
Role 1: Yes. So generally speaking, before this, because I may have planned to have children, I had the awareness of contraception, but it didn't mean that I would always take contraceptive measures. And if I took contraceptive measures, did I usually use condoms?

Role 2: Yes.
Role 1: Have you considered other contraceptive methods?
Role 2: Other contraceptive methods. Now that we have our second child, she has discussed it with me. Now that she is in good health, she has undergone sterilization surgery.
Role 1: It is intrauterine sterilization.
Role 2: Yes.
Role 1: I see. Before you had your second daughter, what were your main reasons for considering contraception?
Role 2: I didn't expect to have another child. I originally thought that one was enough and I would just raise one child.
Role 1: After you had your second daughter, were your reasons for contraception different from before? It sounds like
you both wanted to avoid unwanted pregnancies? Role 2: Actually, we were in this aspect. After we had our second child, my wife didn't want to have a third child.
For me, it doesn't matter. I think the original idea was that one child was enough. If the second child came, then it would come. If we could have a third child by accident, as long as my wife said she was willing to have it, I would have no problem.
Role 1: Actually, from your perspective, you can accept that after having your second daughter, you will continue to take some relatively compulsory contraceptive measures, but because of your wife's ideas, you still respect her feelings.
Role 2: Yes, that's right.
Role 1: I understand. In this contraceptive process, will you consider, in addition to preventing accidental pregnancy, will you consider other factors? Because some people will also consider, for example, avoiding the spread of sexually transmitted diseases, etc.
Role 2: No, we have never considered this aspect. When taking contraceptive measures, she talked about some of her ideas, such as not wanting to have another child, and the pressure of life is too great. I also told her that she doesn't need to consider the pressure of life, but she told me that having a child is like dying once, and she is worried about her health. I agree with her ideas, so there is no problem.
Role 1: It is also the same as other decisions in the family. You two negotiate and discuss, and then finally reach a consensus.
Role 2: Yes.
Role 1: In terms of contraceptive measures, for example, you mentioned that you mainly used condoms before, and there are many other contraceptive measures, such as the safe period, emergency contraceptive pills, or ejaculation outside the body, etc. Who decides what kind of safety measures to use? Or is it also a discussion process?
Role 2: This has not been discussed. I bought the condoms, and she also got some in the community. This is one. I often ejaculate outside the body. This is when I don’t wear that, I ejaculate outside the body.
Role 1: I understand. It’s not that the two of us openly discuss what kind of contraception we should take, but it’s more like a feeling of letting it go. Role 2: Yes
, let it go.
Role 1: Just now I seemed to hear you say that, for example, your spouse would go to the community and get some condoms. In fact, overall, where did you and your spouse learn about contraception? For example, family, friends, or some medical staff.
Role 2: This is all seen and heard a lot, everyone knows it, and no one needs to teach it. We are all adults.
Role 1: Do you remember where you first learned this information?
Role 2: At first, I probably saw TV commercials for condoms.
Role 1: Some information on TV and in the media.
Role 2: Yes.

DOL_036:

Role 1: How have you been using contraception since you met? Because you mentioned that the first time was an accidental pregnancy, what kind of contraception did you usually use before that?
Role 2: Condoms.
Role 1: Did you use safe period or ejaculation outside the body, birth control pills, or other methods?
Role 2: No birth control pills, you said both. Role
1: So did you get pregnant unexpectedly because you used safe period or ejaculation outside the body?
Role 2: Safe period, the first time I got pregnant was during the safe period. Role
1: Were you worried about an accidental pregnancy at that time?
Role 2: I didn't think about it at that time, I just suddenly felt that my period was delayed, and I felt a little nauseous, which was a relatively minor reaction, a physical reaction. Later I bought an early pregnancy test strip and checked it and it said I was pregnant.
Role 1: When you use the safe period method, do you have a different attitude from your husband? Who of you two is more concerned about this matter?
Role 2: No, we didn't.
Role 1: After the first pregnancy, will you change your contraception measures? Or be more cautious.
Role 2: I didn’t use condoms before, but I used them later.
Role 1: I see. When you didn’t use condoms at the beginning, were you worried? What do you think?
Role 2: I didn’t worry before, because my menstrual period is usually very regular, so-called safe period contraception, and there have been no accidents.
Role 1: How did you feel when you were pregnant?
Role 2: I was a little overwhelmed and a little scared.
Role 1: What were you mainly afraid of?
Role 2: Because I got pregnant suddenly, I didn’t know what to do.
Role 1: I’m not ready yet, is that right?
Role 2: Yes.
Role 1: Did you consult other people’s opinions and seek help from others?
Role 2: Yes, I called my mother, and my mother said it was a good thing. Role 1
: What was your husband’s mentality at the time?
Role 2: He was quite happy and was still working hard. He also said it was a good thing and there was no need to be afraid. If you get pregnant, you can just give birth to the baby slowly.
Role 2: Where do you usually get knowledge about contraception?
Role 1: TV propaganda, or local community health and local community propaganda.
Role 2: Do you work in a hospital? Do you know more about this?
Role 1: Yes.
Role 2: What do you do in the hospital? Is it convenient to ask?
Role 1: I am an internal medicine doctor. Role 2:
Do you talk about contraception with your family?
Role 1: No.
Role 2: Why?
Role 1: Because I feel embarrassed to talk about it.
Role 2: Do you talk about it with your friends?
Role 1: No.
Role 2: Is it the same reason?
Role 1: Yes, including me and my child’s father.
Role 2: Why do you feel embarrassed?
Role 1: Talking about this with your elders or your friends may be because of the national conditions of our China, which is not publicized enough. When it comes to this matter, everyone feels embarrassed. If you talk about this matter a lot in your daily chats, it may mean that others will look at you with tinted glasses.
Role 2: Do you talk about related matters with other doctors or nurses?
Role 1: This is also rare, unless someone around us gets pregnant unexpectedly, we will say something about it, such as if you don’t want it, why don’t you take precautions?

DOL_037:

Role 1: Under what circumstances did you absolutely want to have a child?
Role 2: We had a plan, not an accident. We had a plan and an intention, because my husband had to drink alcohol to do this project. When we wanted to have a child, I went to get checked first, because I felt that it was scientific parenting. I don’t know why I was like that at the time, but we paid attention to this. There has never been an accident, including me until now. We have been married for so many years and there has never been any accident, not once. If we don’t want it , we will use contraception. If we want it, we will prepare for pregnancy. At that time, my husband quit drinking for half a year, and I took folic acid for half a year. During this period, I used ovulation test strips to test, and later went to the hospital to test it. It was indeed ovulation, so we had sex, that’s it.
Role 1: Already married? Role 2
: Yes, that’s
right. Role 1: You just said that in fact, you were very careful about contraception during the stage when you didn’t want children. Since you have been together, what methods have you used for contraception? For example, there are.
Role 2: Condoms, nothing else, no medicine.
Role 1: For example, safe period, or ejaculation outside the body, are there any?
Role 2: No, why not? Because I often procrastinate, I can never find out my exact date. I don't know, because my period is often delayed for three, four, or five days. That's it. Why did I prepare for pregnancy before? Because I took Chinese medicine for a while, because my period was always irregular. People said that it's not good to have a child if the period is irregular. I didn't understand this either. We took Chinese medicine for treatment, and then it became smooth. Later, we took folic acid, and we had it.
Role 1: When you might take measures to prepare for pregnancy, what is the main reason? Just don't want to get pregnant? Or are there other considerations?
Role 2: Because I am, I have always been very traditional. I think you can only have children after getting married, and you can't have children without getting married. That's it. I think people say that women are harmful to their bodies. If you get pregnant, it will hurt your body. So that's how we are. He also respects me and never says anything. He also agrees with me that there is no accidental pregnancy. We both got married. You see, we had our wedding in October 2017, November. We had our baby in 2018 because we gave birth in June 2019. It took more than a year.
Role 1: So on the matter of preparing for pregnancy and pregnancy, both sides have relatively consistent opinions?
Role 2: Yes, consistent.
Role 1: Actually, these are almost all our questions. We have some questions about reproductive health, and some of the contraceptive measures we just talked about. I would like to ask where you and your husband first learned about these contraceptive measures, such as talking to family elders, brothers and sisters, or professionals?
Role 2: I feel that contraceptive measures, I don’t know who has been exposed to you, it seems that you subconsciously know about condoms when you go to school. At most, you want to ask who told you this. I feel that you know these words and how to use them when you go to school. I was like that at that time.
Role 1: Did you not specifically learn about it?
Role 2: I didn’t learn about it. No one said anything, including taking medicine. I just read about it online. Oral medicine is not safe during the safe period. In fact, I don’t understand it very well. Sometimes I really don’t understand what the safe period is. It was not until I started to prepare for pregnancy seriously that I learned about the safe period and ovulation period. I didn’t understand before. Why was my stomach bloated? It turned out to be the ovulation period. I didn’t know how to stretch.
Role 1: Later, I gradually gained experience, especially during the process of preparing for pregnancy, I learned more relevant knowledge from the Internet.
Role 2: Yes, but we didn’t try the oral medicine that people said. We didn’t. We always took measures.
DOL_038:

Role 1: You just mentioned that, for example, when you had your second child, you didn't have this plan at first, but then you got pregnant. Since you and your husband met on a blind date, what kind of contraceptive measures have you generally taken? Or do you take some contraceptive measures on your own initiative?
Li Rong: I took medicine.
Role 1: Was it emergency contraceptive pills or short-acting contraceptive pills?
Li Rong: Emergency.
Role 1: Emergency contraceptive pills. The reason for taking this medicine at that time was also because you didn't want to get pregnant, right?
Li Rong: At that time, I had my first child.
Role 1: After having my first child, I thought that I didn't want a second one in the short term, so I took the medicine?
Li Rong: Yes.
Role 1: Did you take other contraceptive measures?
Li Rong: I used condoms.
Role 1: Are there any other ones? For example,
Li Rong: I also had an IUD.
Role 1: I had an IUD. Have you taken it out now? Or after giving birth to the second child?
Li Rong: After the second child.
Role 1: I had the IUD after giving birth to the second child. There are some other common contraceptive methods, such as women calculating the safe period, or men choosing to ejaculate outside the body. Have these ever happened?
Li Rong: No.
Role 1: Okay. What were you worried about when you were considering taking contraceptive measures?
Li Rong: I didn’t want it either.
Role 1: Pregnancy itself, right?
Li Rong: Yes, I felt that the child was not big enough, so I didn’t want it. The first child was not big enough either.
Role 1: Did you consider some other things, such as the spread of sexually transmitted diseases?
Li Rong: No.
Role 1: When choosing what contraceptive method to take, did you and your husband have any disagreements?
Li Rong: No.
Role 1: We were pretty consistent.
Li Rong: Not bad.
Role 1: How did you make this decision at that time? Did you decide to get an IUD inserted?
Li Rong: I put it in myself.
Role 1: You didn’t discuss it with your husband, and you made this decision? You really didn’t want to get pregnant again.
Li Rong: Yes.
Role 1: I understand. Does your husband know about it now?
Li Rong: Yes.
Role 1: What is his reaction now that he knows?
Li Rong: There is no translation. It is quite normal, because it is normal not to want children.
Role 1: He also thinks it is quite normal. He doesn’t want to have any more children.
Li Rong: Yes.
Role 1: Regarding a series of contraceptive knowledge, such as using full condoms, taking emergency contraceptive pills, or inserting intrauterine devices. How did you first learn about this contraceptive knowledge? For example, did you talk with your family and elders, or did you learn from friends, or did you learn from professionals such as doctors and nurses?
Li Rong: Some of it was said by others, and some of it was said in the hospital.
Role 1: It was just from chatting with friends. Sometimes we talked about some things, and then when we were in the hospital, for example, when we had a physical examination or something like that.
Li Rong: Yes.
DOL_039:

Role 1: How have you been doing contraception?
Role 2: No contraception.
Role 1: Will you use the safe period or ejaculation outside the body?
Role 2: I have never considered it. If it comes, it's fine. It depends on whether God will give it to me.
Role 1: Are you worried about accidental pregnancy or disease transmission?
Role 2: Disease transmission does not exist, and accidental pregnancy is not an accident, because we must have a second child. There is no problem with that age. After all, the older one is so old, more than three years old. If you want to consider it, you can consider it. If you don't want to consider it, no one can do anything.
Role 1: So it sounds like you and your wife have reached a consensus on this matter. Since you started dating, you will not take any contraceptive measures. Role 2
: Never, there was a period of time, but I thought about it and let it go. Role
1: Why?
Role 2: If you want it, you have it. I was under a lot of pressure after the Chinese New Year or something. During the epidemic, I didn't have it for two or three months. I asked if I was pregnant? If I was pregnant, I would take it. I took good care of it at home and bought you delicious food and drinks. Later, it was the second and third day after I went to Hainan. I had a stomachache. How could I have a stomachache? Did you feel better after coming to Hainan?
Role 1: Maybe, where do you usually learn about contraception?
Role 2: No need to know, why should I know about it?
Role 1: Like family, friends, doctors, nurses, do you talk to them about related things?
Role 2: I haven't talked to them, it's impossible to say how, I just know which day to compare that.
DOL_040:

Role 1: Indeed, since you met, how have you taken contraceptive measures? Some common measures include condoms, safe period, ejaculation outside the body or contraceptive pills. You heard that you used them twice, which is a little unexpected. What kind of contraceptive measures have you taken all along?
Role 2: Generally condoms.
Role 1: What percentage of people use condoms?
Role 2: Basically 80%.
Role 1: Have you ever worried about unwanted pregnancy?
Role 2: Yes, I have.
Role 1: Are you worried about the spread of diseases?
Role 2: Yes.
Role 1: You and your wife should be using condoms. Have you ever had different opinions, for example, one party does not want to use it and the other party does?
Role 2: Yes.
Role 1: What is the general situation at this time?
Role 2: Anyway, sometimes I feel uncomfortable wearing condoms, so I have never worn them.
Role 1: Your wife thinks it is safer to wear them, right?
Role 2: Yes.
Role 1: If this happens, what will you do?
Role 2: He will take contraceptive pills.
Role 1: I will still choose not to wear it, and then take the birth control pill afterwards. Role
2: Yes.
Role 1: From what channels do you learn about contraception?
Role 2: From friends.
Role 1: Do you talk a lot with friends?
Role 2: Men chat together. Sometimes I just listen to what they mean when I don’t pay attention.
Role 1: Have you talked about this with your family or other people?
Role 2: No.
Role 1: Do you talk about related things with doctors or nurses?
Role 2: I talked to the doctor. Role
1: What do you talk about?
Role 2: There are many kinds of contraceptive measures, so I will know more.
Role 1: Do you think it helps?
Role 2: Yes.
Role 1: In what aspects do you think it helps obviously? Can you talk about it in detail?
Role 2: At least you should be prepared. You can’t come unprepared.
Role 1: Have you learned about contraception from your family?
Role 2: No.

DOL_041:

Role 1: It sounds like your mother is still very concerned about your health. Do you think she would teach you some contraceptive knowledge before marriage or before pregnancy?
Role 2: No, I think parents in rural families feel embarrassed to say these things.
Role 1: So you have learned about contraception through other channels, such as the Internet, friends, doctors and nurses.
Role 2: I know these.
Role 1: Yes, how have you been doing contraception?
Role 2: How do you say it? Anyway, condoms and so on.
Role 1: Some common contraceptive measures may include condoms. Some people will calculate the safe period, some people will use external ejaculation, or contraceptive pills and contraceptive rings. How often do you use condoms?
Role 2: Nothing, no frequency, and nothing. I can't remember it after a long time.
Role 1: Then when you got pregnant by accident for the first time, did you calculate the safe period or ejaculate outside the body or didn't take any safety measures ?
Role 2: It doesn't matter. He didn't count it anyway because it was an accident. It was also an accident. It’s been a long time. It really makes me think that it has been several years, about 7 years, 8 years.

Role 1: I see. Is the second child in the plan?
Role 2: What if the second child is a plan? The second child is like a task. I think my eldest child is already a few years old. When my eldest child was four or five years old, I thought my eldest child was four or five years old. My plan was to have two children. When my eldest child was in the first grade, my youngest child could go to kindergarten. At first, I was very resistant. Later, my colleague chatted with me and said, "Let's have it." He already had a second child. Then he persuaded me, "Let's have it. It's different to have a second child. How about a second child?" Then I was tempted. Then I thought that sooner or later I would have to do this. Why not bring the time forward
? Role 1: So it sounds like the people around you, including some of your colleagues and family members, will actually affect you.
Role 2: Yes, it is because their thoughts have already influenced me. When I just gave birth to my first child, I was very resistant at that time. I didn't want to have it because I had too much morning sickness. I was very resistant. Later, they just said that time has passed. It's really healed and forgotten the pain. People forget how painful it was to have a baby after a long time, and then they persuade them to do so.
Role 1: I see. After you have a second child, will you and your husband be more cautious about contraception? Because it sounds like you don't plan to have a third child. Excuse me, how will you be more cautious? Role
2: Condoms.
Role 1: So it sounds like you will use them more frequently, right?
Role 2: Yes.
DOL_042:

Role 1: Got it. So when you decide not to have children, what kind of contraceptive methods do you usually use? For example, safe period, IUD, you said. You just had it recently after giving birth to your second child, right?
Role 2: It's been several years.
Role 1: It's been several years. It sounds like you are very determined and don't want to get pregnant again. So in the process of thinking about what kind of contraceptive measures to take, do you and your husband have similar opinions? In other words, there are some differences.
Role 2: No. Because he is on a business trip today, we rarely do it.
Role 1: Not very often.
Role 2: Yes. It's not unusual, but it's very rare.
Role 1: Okay.

Role 2: He had never thought about this issue. Later, he got pregnant unexpectedly once, and then he said that he was determined not to take the medicine. Then he found that the doctor who went to the hospital in the first week looked at me in surprise and asked me if I had just found out. I said that I would not take it if I found out.
Role 1: Haha. I understand. Before getting an IUD, for example, if you thought about contraception, what methods would you usually take?
Role 2: I haven't thought about it.
Role 1: Because everyone is just talking.
Role 2: Really rarely.
Role 1: You rarely think about contraception methods carefully, right?
Role 2: Yes, and then let's talk about family conditions. He often travels on business, and we are at home, and we have children. We rarely have the methods you mentioned.
Role 1: For example, we don't have other methods such as calculating the safe period, condoms, ejaculation outside the body, or contraceptive pills.
Role 2: No. The community health center gave me two boxes, and there are still two boxes there.
Role 1: I understand. So in general, where do you usually learn about contraception knowledge? For example, how did you learn about getting an IUD?
Role 2: Community health, we have family doctors in community health, there will be (promotion).
Role 1: It is mainly about some things that the community health doctor promotes, and tells you that it is okay. Role 2:
I think it seems that biology classes have it, not all junior high schools have it.
Role 1: Then there is biology class, some childhood memories, some sex education about how to receive adolescents, etc.
Role 2: Yes, there are some books, and there is nothing else, you will know if you go to the hospital more. Role 1:
It seems that I have learned some things in the hospital. Role
2: After you give birth, the doctor will give you a lecture every time you give birth. Every time you give birth, maybe I don’t know about Shenzhen, I don’t know about other cities, when you are pregnant, you will go to the hospital for the first time to open a file, and then there will be a lecture classroom, and then they will tell you everything, if you don’t want it, you will go to some gynecology department or something, and go to another department to give you some contraceptives or something like that, anyway, the hospital said there is a lecture, even if you want the child, even if you want it, they will also give lectures, so basically when you reach adulthood, if you say you want to get pregnant, you want to go to something, basically you will be lectured.
Role 1: It is true that the supporting services in Shenzhen are quite complete.
Role 2: Yes, normally, before we got married, the community health center would have some pre-pregnancy checkups, prenatal checkups, and brochures, etc. Anyway, there are all kinds of services.

DOL_043:

Role 1: Raising children still requires a lot of time and energy. The next question, the last one, is about some issues related to reproductive health. Have you and your husband ever considered contraception since you met? Role 2: I think
so.
Role 1: When you don’t want children, you usually take contraceptive measures.
Role 2: Yes.
Role 1: So what kind of contraceptive measures have you taken? Generally speaking, there are methods like condoms, birth control pills, and ejaculation outside the body. Role 2
: Condoms.
Role 1: Mainly condoms.
Role 2: Yes.
Role 1: You just mentioned that it’s mainly because you don’t have the energy to raise another child. After this, will you always be more careful and take contraceptive measures to avoid getting pregnant again?

Role 2: Yes.
Role 1: Have you ever considered contraception for any other reason?
Role 2: No, I just don't want to have any more children.
Role 1: In the process of taking contraceptive measures, whether to use condoms, etc., did you and your husband make the same decision? Are there any differences of opinion?
Role 2: No, he listens to me.
Role 1: I will take care of the child, so he will definitely listen to me.
Role 2: He can see that it is really hard for you to take care of the child.
Role 1: I also want to know where you learned about contraception?
Role 2: Didn't you give me a book when you got married? You can search for this information on the Internet.
Role 1: The information on the Internet, the book you gave me when you got married, are you referring to some manuals on eugenics?
Role 2: Yes, there is no contraception written in eugenics.
Role 1: In addition to some information on the Internet, have you talked to family members, elders, or friends, or other professionals, such as doctors and nurses, about this information?
Role 2: Probably not.
Character 1: I mainly rely on books and websites.
Character 2: Yes, how can I ask my elders about this?
DOL_044:

Role 1: That's true. We just talked about children, and I want to ask about children. Under what circumstances did you and your husband decide to have your first child?
Role 2: This was an accident.
Role 1: Before marriage? Or after marriage?
Role 2: When we were getting married, we didn't use contraception, and we got pregnant the next month. When we got pregnant, it wasn't long before we got married.

Role 1: Next, we may ask some questions related to reproductive health. We also mentioned that the first and second children were both wanted children, but they happened to get pregnant without being prepared. Have you and your husband ever considered taking contraceptive measures in the process of getting along?
Role 2: Yes.
Role 1: What methods do you usually take? Sometimes people will consider using condoms, or women will consider the safe period, or intrauterine contraceptive rings, or taking contraceptive pills, in vitro fertilization, etc. There are various methods. Which one or which ones do you take? Role
2: At first, I used condoms, and then the safe period, but the safe period was not very accurate. Later, I also tried in vitro fertilization. Role 1:
I have tried different methods.
Role 2: But I also got pregnant. Once after giving birth to my first child, I got pregnant, but I didn’t know it at the time. I also caught a cold in the middle and took some medicine. I was afraid that the medicine would be bad for the future child, so I didn’t keep it.
Role 1: At the beginning, I used more condoms. What was the opportunity that made you consider using more safe periods or in vitro fertilization as a contraceptive measure later? What is the reason for the change?
Role 2: My husband said it was more troublesome. Because he felt it was not as comfortable as not wearing it.
Role 1: A difference in experience. When you were considering what kind of contraceptive measures to choose, did you two discuss it together, or who made the main decision? Were there any disagreements?
Role 2: No, he bought the things.
Role 1: Basically, your husband was responsible for buying condoms, and he decided what kind of contraceptive measures to take, and you were generally more cooperative. Role 2
: Yes.
Role 1: Later, when you chose to take contraceptive measures, what was the main reason? Was it because you didn't want to get pregnant? Some people would worry about the spread of diseases, etc. Role
2: At that time, it was because I was afraid of getting pregnant, because the children were still young. If you had two children too close together, wouldn't you be unable to take care of the children? When I gave birth to the first child, I didn't think about having a second one right
away. Role 1: How many years did you think it would take to have a second child?
Role 2: At that time, I thought four to five years would be better.
Role 1: I would also like to ask about these knowledge related to contraception. Where did you learn it? Did you learn about it online, or did you ask your family elders about contraception, or did you talk to your friends, or did you talk to professional doctors and nurses about it? How did you learn about the various methods?
Role 2: At that time, we used condoms because everyone knew about it, so it was safer to use condoms. Later, I accidentally got pregnant once. I went to Huashan Hospital in the city, which is a relatively large hospital. I have a relative who works there, so I went to see them. I was pregnant at the time, and I didn’t want to say that I didn’t want him. I thought I would have sex if I was pregnant. Didn’t I say that I took some medicine, but I was afraid that it would be bad for the child. When I consulted the doctor, the doctor had also experienced this problem and told couples to be more careful about contraception in the future.
Role 1: Anyway, there are two aspects. One aspect is natural. Everyone around me knows that condoms are used for contraception. The other aspect is that I also talked about it with the doctor.
DOL_045:

Role 2: I see. Under what circumstances did you decide to have a child?
Role 1: We had it naturally, without any contraception. Then we had it
when we got pregnant. Role 2: So did you get pregnant before or after marriage? You got pregnant after marriage, right?
Role 1: Yes, after marriage.
Role 2: I probably didn’t think about contraception after marriage. I had it as soon as it came.
Role 1: I had it as soon as it came.
Role 2: Under what circumstances did you have the second child?
Role 1: I found out I was pregnant again when the first child was over two years old, and then I gave birth to it.
Role 2: It was relatively natural, right?
Role 1: Yes, I had both children by natural birth, not by caesarean section.
Role 2: How did you use contraception all along?
Role 1: I could use an IUD.
Role 2: When did you start using it?
Role 1: I had an IUD when the second child was two years old.
Role 2: So you made a decision at that time not to have a third child, right?
Role 1: Right.
Role 2: What contraceptive measures did you usually take before this?
Role 1: I didn't take any contraceptive measures before this. I only knew about the IUD after I had my second child. I didn't take any measures before.
Role 2: Just condoms, and I didn't use anything else, right?
Role 1: I didn't use it. Role 2
: I see. Before you got the IUD, did you have any concerns about unwanted pregnancy? Role
1: Let me think about it. No, I didn't have any such concerns.
Role 2: I see. Do you worry about the spread of diseases?
Role 1: No, because my husband is often at home, so I don't worry about the spread of diseases. I believe in him and he won't mess around outside.
Role 2: Yes, who decided to get the IUD between you and your husband?
Role 1: I said at that time that I already had two children, so I decided to get the IUD, and he said it was okay.
Role 2: Yes, did you consider other contraceptive measures besides the IUD at that time? For example.
Role 1: I listened to the doctor's advice. The doctor suggested that we could get the IUD like we did. I went to the hospital and asked the doctor.
Role 2: I see. Do you learn about contraception from your family?
Role 1: Because I don't have a mother-in-law, I don't have one either. I really don't have one.
Role 2: What about your parents?
Role 1: No, my parents don't tell me either.
Role 2: Do you talk to your friends about contraception?
Role 1: Sometimes, I talk to my cousin when we sit here, because we have a good relationship. I will talk to him about private matters.
DOL_046:

Role 1 : I understand. When you wanted your first son, under what circumstances did you have the chance to have him?
Role 2 : I forgot.
Role 1 : Was it before or after marriage?
Role 2 : Before marriage.
Role 1 : So you got pregnant quite naturally, and decided to give birth to him, right?
Role 2 : Yes.
Role 1 : Did you decide to get married because of the pregnancy?
Role 2 : No. Because we were engaged before, and planned to get married during the Chinese New Year, but after getting pregnant, we got married two or three months earlier?
Role 1: I understand. How have you been using contraception all along?
Role 2: Wearing an IUD, using condoms.
Role 1: So you should have worn the IUD after giving birth to the second child, right?
Role 2: No, I wore the IUD after giving birth to the first child, and then removed it when I was preparing to have the second child.
Role 1: What do you think? I heard that sometimes the process of removing the IUD is more difficult. Do you feel this way?
Role 2: No, because I changed it in the middle, that is, I changed it every few years. If you don't change it for a long time, it will have an impact. It will be painful to remove the ring, but it has side effects.
Role 1: I understand. Do you use condoms frequently? Role
2: Not very often.
Role 1: Are you worried about accidental pregnancy?
Role 2: I am worried.
Role 1: Will you communicate with him? I want him to use condoms better.
Role 2: Yes, but he is also afraid of what we will do if we get pregnant again.
Role 1: So it sounds like he may listen to your communication.
Role 2: Yes.
Role 1: Is there a time when he doesn't listen?
Role 2: No.
Role 1: That's good. When you first started dating and got married, because it sounded like you wanted to have a child naturally, at that stage, who decided not to take contraceptive measures?
Role 2: We didn't have this common sense before. I don't know if he had it, but I didn't have it.
Role 1: I understand. Where do you usually learn about contraception later? Can you hear me? Hello, I couldn't hear you just now, but I can hear you now.
Role 2: I read it in a book and heard others talk about it. Role 1:
Understand, who are the others?
Role 2: Friends around me, such as friends and colleagues around me.

DOL_047:

Role 1 : You and your wife are under great financial pressure, especially considering that you have two children to raise and a mortgage. It sounds like you are not considering having a third child for the time being or in the future. How have you considered and adopted contraceptive measures?
 Character 2 : This is artificial.
 Role 1 : Because of some specific measures, for example, the ones that people often use are like using condoms.
 Role 2 : Use condoms.
 Role 1 : Have you tried other methods? For example, girls consider the safe period or going, especially after giving birth to two children, using contraceptive rings or contraceptive pills, and some methods such as external ejaculation in the south, etc. Have you considered other methods?
 Role 2 : None else.
 Role 1 : Mainly condoms.
 Role 2 : She said that this thing is bad for the body or something. She said that taking medicine or something is not good, but if there is a safe period, then that thing is not necessarily safe.
 Role 1 : One is to consider the side effects, and the other is to consider whether it is really safe. I feel that condoms are safer.
 Character 2 : Yes.
 Role 1 : I see. What was the main reason why you and your wife took contraceptive measures?
 Role 2 : If you were to get pregnant again, would you consider this? You said you had not considered having another baby.
 Role 1 : Are you mainly worried about pregnancy?
 Character 2 : Yes.

Role 1 : Got it. Your wife will be like this: when you and your wife decide whether to use a certain method, such as using condoms as a form of contraception, do you two agree?
Role 2 : The opinions should be unified.
Role 1 : Have there been any small disagreements? Role 2 : No. Because it has always been like this, no one has any other ideas, because she doesn't like to use anything else.
Role 1 : Got it, and actually haven't thought about other options.
Role 2: Yes, she considered it, but she thinks that it is not very good.
Role 1: Mainly because of some considerations in terms of health and safety.
Role 2: Yes, so this is still better.
Role 1: Got it, it seems that your wife and you are actually more concerned about one is safety, and the other one seems to be the one you mentioned first, which is health.
Role 2: Yes, I definitely think so.
Role 1: Understand. Where do you usually learn about contraception? For example, do you learn about it from the Internet, or do you learn about it from your family elders, because they are more experienced? Or do you talk to your friends, or doctors and nurses?
Role 2: You learned about this from your friends on the Internet. The Internet is so developed now, you can learn about it anywhere on the Internet. Some things you know naturally, right?
Role 1: Yes, I just feel like I know it naturally when I grow up.
Role 2: Yes, some things you know naturally, you don’t have to learn them deliberately, right?
DOL_048:

Role 1 : Yes. Is that so? Next, I might also like to ask about some issues related to reproductive health, especially considering that you just said that you only planned to have one child. After you and your husband had your first child, how did you take contraceptive measures? Or did you take the initiative to take contraceptive measures?
 Role 2 : Yes.
 Role 1 : What kind of contraceptive measures are generally taken?
 Role 2 : My periods are not very regular because I was not weaned until I was two years old. Now I am two years and four months old and my periods are still not regular. We did not use any contraception during this period because my periods would not come. I was very nervous when I was just weaned. I basically used condoms. Later I found that it was not very frequent. My periods are not regular either. Besides, we rarely see each other ( 00:40:50 ). It's that kind of thing.
 Role 1 : I see.
 Role 2 : I didn’t take any birth control pills on purpose.
 Role 1 : Got it. Now I am considering not having children in the future, for example, contraceptive methods like intrauterine devices.
 Character 2 : Impossible, I wouldn't think of that.
 Character 1 : OK, I understand. So the main reason for contraception is because you don’t want to get pregnant again.
 Character 2 : Yes, is the intrauterine contraceptive device anti-human? That is something from the old days, and now it has been proven that it is not good for girls.
 Role 1 : Yes. You just said that when you choose different contraceptive methods, such as using an in vitro fertilization method or using a condom, do you and your husband have any disagreements when deciding which method to use?
 Character 2 : No.
 Role 1 : These are relatively unified opinions.
 Character 2 : Yes.
 Role 1 : OK. The last question is about contraception. I want to know where you learned about contraception, especially what you just said, such as which one might be less healthy for girls, etc. How did you learn about it?
 Character 2 : I told you, I used to work in the pharmacy over there.
 Character 1 : Yes.
 Role 2 : I work in the pharmacy of the maternal and child health care center over there. How could you not know these things? Do you know the family planning center? The place where family planning sterilization is done for contraception, you have seen too many of them in the hospital. There are many people who bleed due to rejection reactions, and there are also many who are unable to have children for life. Because they want to have children, they have to open the fallopian tubes and so on. I know it is against humanity. It is related to my work experience.
 Role 1 : Indeed. So in fact, to some extent, you are also a professional, so you have learned a lot of such knowledge in the workplace. This is different from the knowledge you had before you started working, such as the knowledge you learned about the causes of the disease from friends, family elders, or the Internet.
 Role 2 : Friends rarely talk about this topic, and parents won’t talk to you about it. My parents never talked to me about this, but there might be some time on the Internet, right? I would Baidu some information, or for example, I just had my period, I just had my first period after I turned two, I just had my period after I was weaned, and we discussed what to use, and he took the initiative to ask to buy it. I said okay, and then we used condoms.
DOL_049:

Role 1 : Yes. Okay, thank you. I know we talked about this when we were talking about children just now. We didn't plan to have a baby at first, but we welcomed this baby. I want to know that from the time you and your husband met and got to know each other, to the time you just mentioned getting married in name, and then to the time you got married officially, that is, during different stages, did you consider taking contraceptive measures?
 Role 2 : The contraceptive measures we have are mainly condoms.
 Role 1 : Mainly condoms.
 Role 2 : Have you tried anything else?
 Role 1 : None else.
 Role 2 : Now that the second child is born, we have decided that we don’t want another one anyway, so we chose to have an IUD inserted.
 Role 1 : Has an IUD.
 Role 2 : Because I think it’s safer.
 Character 1 : Yes. The main reason for taking contraceptive measures is to avoid getting pregnant again, right? It's mainly a consideration.
 Character 2 : Yes.
 Role 1 : Got it. You and your husband are deciding what kind of contraceptive measures to take, such as whether to use condoms before, and whether to get an IUD after having two babies. Are you consistent in all these processes? Or are there some disagreements?
 Role 2 : We were quite consistent in our opinions at the time. He just put me first. As long as the requests I made were not excessive or anything, he would always go along with me.
 Role 1 : Sure enough, it's very sweet. Okay. Where do you and your husband usually learn about contraception? For example, maybe you talked to your elders at home, or talked to friends, or talked to professional medical staff. Is there such a way to learn about it?
 Role 2 : We usually search on Baidu and then go to the hospital for consultation.
 Role 1 : First search on Baidu and the Internet, and then go to the hospital to ask after you have some basic information.
 Character 2 : Yes, because if I ask others, I always feel as if I am embarrassed to say it out loud, as it feels too private and confidential
.

Role 1 : Before you and your husband decided to have a child, did you consider taking the initiative to use contraception because you were observing him?
 Role 2 : Yes, I want this. If you don’t want a baby, you definitely want it.
 Role 1 : At that time, I was mainly considering not wanting to get pregnant and was not ready to take any measures.
 Role 2 : Yes, you can’t rely on luck, including if you don’t want to have a baby in the future, you have to do this, you can’t rely on luck. So we first have a clear understanding of this kind of thing, and we don’t relax at all.
 Role 1 : Yes, so what kind of contraceptive measures have you taken before?
 Role 2 : It’s a condom.
 Role 1 : Mainly condoms, right? Have you tried, for example, some short-acting oral contraceptives or emergency contraceptives, or ejaculation outside the body?
 Role 2 : I have also taken the oral emergency contraceptive pills you mentioned, but not much because they are harmful to the body.
 Role 1 : Yes. Now let me know how you first learned about this series of contraceptive measures?
 Role 2 : Contraceptive measures. I think this thing is so big now, and everyone should know something about it, right?
 Role 1 : Did you learn about this from the Internet?
 Character 2 : The key point is that he is not a teenager and does not understand at all. If he were a teenager, he should understand it. I think this is common sense.
 Character 1 : I don’t have much recollection of this, so where did you first learn about it?
 Role 2 : Because my husband is not my first, so we all seem to know this at the same time.
 Role 1 : It’s not the state of an ignorant teenager.
 Character 2 : Yes.
 Role 1 : When choosing a contraceptive method, do you and your husband have relatively consistent opinions, or do you sometimes have some disagreements?
 Role 2 : No disagreement, that's fine. But you know men are not always calm, they may be impulsive sometimes, but they are calmer. I am calmer in this respect, maybe it has something to do with my education, so I absolutely do not allow myself to get pregnant before getting married. It is absolutely not allowed.
 Role 1 : So even though your husband may not be calm sometimes, you are always quite persistent, so he can respect your suggestions and opinions.
 Character 2 : Respect, he totally respects.

DOL_051:

Role 1 : I understand. Okay. You just mentioned that it was your first accidental pregnancy when you gave birth to your child, but since you didn’t want to have another child, you firmly took contraceptive measures, right?
Role 2 : Yes, that’s right.
Role 1 : How have you been taking contraceptives all along?
Role 2 : For contraception, she took pills before, and I thought that taking pills might be more or less bad for the body. So I took.
Role 1 : In general, people will take some measures, such as using condoms, safe period or ejaculation outside the body, etc.
Role 2 : Basically, it’s staggered safe period or condoms.
Role 1 : Okay, I see. You just said that your wife took pills before. Was it the emergency contraceptive pill, or the short-acting contraceptive pill? 333 is the emergency contraceptive pill.

Role 1 : I see. Which of these contraceptive methods have you used more often?
 Character 2 : Wear the full set.
 Role 1 : Wearing a condom. When discussing what contraceptive method to use, how do you usually make a decision? When discussing what contraceptive method to use, how do you and your wife usually make a decision?
 Role 2 : You can discuss it beforehand, and then after agreeing on it, you can do it according to that method.

DOL_052:

Role 1 : It sounds like the state of your wife's parents trying to induce labor had an impact on you and your wife, but in the end, you two seemed to have reached a consensus to let nature take its course.
 Character 2 : Yes.
 Character 1 : So when the baby was really conceived, you just said that to some extent you were not fully prepared.
 Role 2 : I wasn’t prepared much at the time, just like my wife said, I just let things take their course.
 Character 1 : Was this an accidental pregnancy?
 Role 2 : But my wife has always been paying attention to this, so it’s not a surprise, but it’s also a surprise. It’s hard to say.
 Character 1 : You said that your wife has been paying attention to these things, for example, she has been calculating her ovulation period, and does that mean she doesn’t take any contraceptive measures?
 Character 2 : That’s about what I mean, yes.
 Role 1 : I understand. If you have been taking contraceptive measures, what methods do you use?
 Role 2 : In the beginning, I used drugs more often. Finally, during a meal, I met a friend in the medical field. He told me that the drug itself is harmful to the body. If used for a long time, it may cause some kind of physical discomfort or bad things. His advice was very good. After listening to it, he suggested choosing condoms. Although they are made of rubber materials, you should not buy the cheap ones, but buy better ones, which are still good for the body and much better than taking the medicine. Then I changed.
 Character 1 : I see. So what you took at the beginning was the emergency contraceptive pill or the short-acting contraceptive pill?
 Role 2 : Short-acting ones should be taken in advance.
 Character 1 : The kind that you have to eat all the time, or the kind that you eat after having sex?
 Role 2 : Eat beforehand, and then nothing happens after sex, what is it called? I can't seem to remember the name, and it's useless now.
 Role 1 : Prepares something to eat before having sex.
 Character 2 : Yes.
 Role 1 : How did you decide to use this contraceptive pill as a contraceptive? Before you knew it might be harmful.
 Role 2 : At the time, I thought that medication would be faster, and then I saw that condoms had leaked a lot in the past, so I felt it was unsafe, so I chose medication.
 Character 1 : I see. So what was the main reason for using contraception before? It was because I didn't want to get pregnant.
 Role 2 : When we had our first child, we didn’t have parents to take care of the child, and we were worried about hiring a nanny, so we took care of the child all the time. It was very tiring to take care of the child until he was a few years old. It was really unbearable to take care of a child alone. I was quite busy at that time, so I didn’t take care of the child much. She was alone and didn’t work before. It was very hard to take care of the child alone at home. I understood and I have always been grateful. After we had the first child, I thought that when the child grew up and went to school, if we wanted, we would have another one.
 Role 1 : Mainly because I feel too busy. It is difficult to balance child care and work, so I feel that I cannot have a second child in such a short period of time, so I took safety measures. Sorry, the network seems to be not very good now.
 Role 2 : Yes, that's right.
Role 1 : Can you hear me now?
Role 2 : Yes.
Role 1 : When you used safety measures, in addition to worrying about pregnancy, did you consider preventing the spread of sexually transmitted diseases?
Role 2 : We are very careful about this, because we will pay attention to this before doing anything, and then take measures for cleaning or care.
Role 1 : After learning from a doctor friend that contraceptives may not be so healthy, how often do you use condoms?
Role 2 : Generally, we still count the safe period.
Role 1 : If there is a safe period, we may not use it, but outside of this, we will use it.
Role 2 : Yes.
Role 1 : Have you taken other contraceptive methods? Because you just mentioned condoms, safe period, contraceptives, for example, there are some other methods such as ejaculation outside the body, etc.
Role 2 : Ejaculation outside the body is relatively rare, not too much.
Role 1 : I understand. Generally speaking, when to consider the safe period and when to use condoms, for example, if you don't use condoms, but it is your wife's safe period, will your wife ask you to use condoms because she is worried about pregnancy?
Role 2 : Now that the child is older, she doesn't take such measures too much. In the past two years, they paid more attention to this aspect, because we were all burdened, and she was more strict about this. Now we can basically take a breath. And there are not too many requirements for this.
Role 1 : I understand. Before, because it was really too stressful to take care of the child, she would be more persistent. Your wife would be more persistent.
Role 2 : Yes, because women take care of children for a long time, their ideas will be a little different.
Role 1 : What are the main differences?
Role 2 : Emotions, that kind of irritability can be reflected.
Role 1 : It means that especially during the process of having sex, the mood may be a little worse.
Role 2 : Yes.
Role 1 : I understand. Generally speaking, if your wife asks you to use condoms, if there is a disagreement in this situation, how do you usually deal with it in the end?
Role 2 : There won't be much disagreement on this. Basically, the disagreement is not big.
Role 1 : If she brings it up, you won't think it's a disagreement. Basically, you can still reach an agreement.
Role 2 : Yes.
Role 1 : Got it. If we talk about using condoms among all the contraceptive methods, who mainly brings it up? Generally speaking, it's between you two.
Role 2 : Generally, she brings it up more.
Role 1 : Got it. What about when you used birth control pills before?
Role 2 : It seems that I brought it up when I was taking birth control pills.
Role 1 : Were you also worried about pregnancy when you brought it up?
Role 2 : Yes.
Role 1 : Got it. In general, do you remember where you learned about these contraceptive knowledge? I know you just mentioned that you may have talked to your doctor friends. In addition, some people will talk to experienced elders at home, or talk to friends about contraceptive-related matters, as well as some other medical staff. In general, where do you think you learned more?
Role 2 : Generally, I read more from the Internet.
Role 1 : On the Internet, I searched online and got some information.
Role 2 : Yes.
Role 1 : Do you think this knowledge is helpful to you and your wife in your life?
Role 2 : What is said on the Internet seems to be a little different from what is done in reality. His online practices are very talkative. When playing with friends, I feel that they still choose drugs and condoms more often, and other things are very rare.
Role 1 : I understand. There is too much information on the Internet. In fact, some of it is not so certain and not so helpful. It may be more helpful to refer to the experience of friends.
Role 2 : Yes.
DOL_053:

Role 1 : Was this pregnancy a planned pregnancy or was it unexpected?
 Role 2 : I didn’t have a plan. I didn’t think too much about it at the time. I just let things take their course.
 Role 1 : I didn’t take any contraceptive measures or have any plan, I just got pregnant.
 Character 2 : Yes.
 Character 1 : I see. That's very hard. Have you ever taken any contraceptive measures?
 Character 2 : Yes.
 Role 1 : What contraceptive measures have you taken?
 Role 2 : It’s given to you by the family planning department throughout the year for health care.
 Role 1 : They will give out condoms, right? I see. In addition to condoms, have you used any other contraceptive methods?
 Character 2 : They are all birth control pills.
 Character 1 : Is it the kind that you take when you need contraception, or is it the kind that you have to take all the time?
 Role 2 : The kind you eat when you need it.
 Character 1 : OK, I see. Are there any other contraceptive methods?
 Character 2 : No.
 Role 1 : What was the main reason you took contraceptive measures at that time?
 Character 2 : I don’t want so much, two sons are enough.
 Role 1 : I thought about the contraceptive method I adopted after I had two children and gave birth to two sons.
 Character 2 : Yes. Those two sons are under enough pressure.
 Role 1 : Definitely, that's right. Do you think you would ask your husband to use condoms during sex because you are worried about pregnancy?
 Character 2 : That's for sure. Otherwise it's not good for your health.
 Character 1 : Yes. Did you have any conflicts or disagreements during this process?
 Role 2 : There is no middle ground.
 Role 1 : You mentioned that it is generally okay. Okay. When deciding which contraceptive method to take, for example, you just mentioned using condoms, some condoms distributed by the family planning department, or taking oral contraceptives. How do you make decisions when taking different methods? For example, when choosing between condoms or contraceptive pills, how do you decide? Which contraceptive method do you choose?
 Character 2 : There is neither this nor that. How should I answer this?
 Role 1 : Do you have different opinions on this? For example, the man wants to use contraceptive pills, and you may prefer condoms or something like that.
 Character 2 : Not this.
 Role 1 : Who usually makes the decision?
 Role 2 : It is usually decided by my husband.
 Role 1 : It is to decide which approach to adopt. If you have different opinions, someone will have the final say.
 Role 2 : In the end, one person always has the final say.
 Role 1 : I see. Does your husband usually make this decision? Where do you usually learn about contraception?
 Character 2 : I don’t know what to say. They would promote it when they have time, but they haven’t promoted it much in the past few years since it was relaxed. In the past, they always promoted contraception and so on.
 Role 1 : Understand that some professionals from the Family Planning Office will come to promote contraceptive support.
 Role 2 : Understand.
 Role 1 : Do you talk to other people? For example, family members who have experience with family members, or friends or other people.
 Character 2 : No.
 Role 1 : OK. Do you think the knowledge promoted by the Family Planning Office is helpful for you and your husband to take contraceptive measures?
 Role 2 : There should be.

DOL_054:

Role 1 : Okay, next I would like to ask some questions related to reproductive health. Have you and your wife always taken any contraceptive measures?
 Character 2 : Yes.
 Role 1 : What approach is generally taken?
 Role 2 : Just wear a condom. The main thing is the condom.
 Character 1 : Have there been other ways?
 Character 2 : No more. I can't handle your fancy tricks.
 Role 1 : Because these are other more commonly used methods. You may also calculate the safe period, or take birth control pills, or have external ejaculation, etc. There are also some intrauterine contraceptive devices or contraceptive rings. Have you tried these?
 Character 2 : Nothing fancy, I haven’t thought about that aspect.
 Role 1 : OK. Got it. What are the main reasons why you take contraceptive measures?
 Role 2 : Avoid having a third child. Although the country is now open, you still don’t want to have a third child.
 Role 1 : What are your considerations?
 Role 2 : Whether you have personal pressure or not, if your family conditions can support it, then there is no pressure at all and you don’t care.
 Role 1 : I understand. It’s still a matter of the financial pressure of raising a child.
 Role 2 : You only have a few decades in this life. It would be different if you lived happily or lived with a mountain on your back like you do.
 Role 1 : So, will your wife ask you to use a condom because she is worried about pregnancy?
 Character 2 : I guess so.
 Role 1 : Do you sometimes have disagreements on this matter?
 Character 2 : Not this.
 Character 1 : OK, I understand. Who usually makes the main decision about which contraceptive method to use?
 Role 2 : Never shown.
 Role 1 : It is difficult to tell who makes the main decision.
 Character 2 : Yes.
 Character 1 : Is it more of a natural thing to do, or is it something like that?
 Role 2 : Let it be.
 Role 1 : Got it. Where do you usually get this contraception-related knowledge?
 Role 2 : Do you still need to understand? All these ideas and knowledge are spread to you by society. Do you still need to understand?
 Role 1 : It means that I feel that I have not taken the initiative to understand.
 Role 2 : You don’t need to understand at all.
 Role 1 : Where do you think you got this information passively?
 Role 2 : After you enter society, the people and things around you, including the short videos you watch, are all of them.
 Role 1 : There will be some information on the Internet that will enter society. Your friends or people you have met may more or less mention and talk about this aspect, right?
 Character 2 : Yes, you can’t avoid men.
 Role 1 : Do you think the information on the Internet and from your friends is helpful in helping you understand contraception?
 Character 2 : Definitely. You never stop learning.

DOL_055:

Role 1: Okay, next I might want to ask you some questions about children. I know you just said you have two sons. What was the situation that made you and your husband decide to have children?

Role 2: I let the first child take its course, and we kept it since we were married. As for the second child, to be honest, I didn’t want a boy at first, but my belly was getting bigger and I was having fetal movements, so I couldn’t bear to give it up, so I kept it.

Role 1: Yes. OK, next I would like to ask you about reproductive health. Because you just mentioned that your second child was conceived by accident. Have you and your husband taken any proactive contraceptive measures since you met on a blind date?

Role 2: I started using contraception after I had my first child.

Role 1: What kind of approach will generally be taken?

Role 2: Because we definitely wore condoms, because we also have IUDs here. At that time, my husband said that IUDs are bad for the body, and I was also afraid. I heard those older people say that those were all aunties, right? They said that IUDs are definitely not good, and they said that when the IUD is removed, the flesh will be taken down and used. I was afraid when I heard that, and I was against IUDs, and my husband also said that it was not so bad, so we definitely chose to wear condoms.

Character 1: Why did you choose contraception at that time?

Role 2: I feel this is the safest option. Taking medicine is not safe and I am afraid that taking medicine will harm my body.

Role 1: So the main reason was that after giving birth to my first child, I didn’t want to get pregnant in the short term.

Character 2: Not at that time. In the short term, I didn’t want to have children, and that’s it.

Character 1: Got it. In addition to condoms, have you ever used other contraceptive methods?

Role 2: No. I took the pills twice, but I felt that it had some effects on my body, which women can more or less feel. It was definitely not good, and it caused some inflammation. So I stopped taking it and started using condoms all the time. Plus, my husband was not at home for long periods of time. He only came back a few times a year.

Role 1: I see. Do you sometimes ask your husband to use condoms, for example because you are worried about getting pregnant?

Role 2: Yes, I will definitely say it. Sometimes he doesn’t want to wear it, but I will definitely ask him to wear it. I don’t want to suffer either.

Role 1: Yes. If, for example, your husband doesn't want to wear it, but you don't want to get pregnant, how would you handle this difference of opinion?

Character 2: I will force him to wear it.

Character 2: Of course I will, because if he doesn’t wear it I definitely won’t give it to him, right?

Role 1: How do you usually decide who is the main person in charge of deciding what contraceptive method to use? For example, when choosing condoms or oral contraceptives, who is the main person in charge of deciding?

Role 2: At the beginning, I used condoms. When I kept using condoms, he said that it was my husband's decision. He bought them himself, right? As a woman, I was definitely embarrassed, but as a man, he didn't care. He was an adult and everyone knew about it. So he went to the pharmacy to buy them. Then, we ran out of condoms twice. That's when I bought medicine. I bought emergency contraceptive pills twice. Emergency contraceptive pills are effective for three days. Then I told him not to drink this medicine. Then I looked up those medicines on the Internet. Drinking too much is not good for the body, right? Then I asked him to buy more, and he did.

Character 1: So the emergency contraceptive pill is because you were having sex at the time, but there were no condoms at home, so you said you would go buy the pill.

Character 2: Yes, because I drank it. I took the emergency medicine twice. People said that you only need to take it once or twice a year, and then I was afraid that it would be bad for your body, so I asked him to buy it. Then I told him to buy more if he wanted to, and keep it at home. It won’t go bad anyway.

Character 1: So there were no birth control pills or condoms at home at that time, but you told your husband that he could go buy birth control pills.

Character 2: Birth control pills, you mean birth control pills, right?

Character 1: Yes, I mean, under what circumstances do you decide to use birth control pills?

Role 2: I said that he didn't use contraception because there were no condoms at home and I was afraid of getting pregnant, as I am a person with a fertility physique. I was afraid of getting pregnant, so I told him to go to the street and buy some contraceptives, and then I asked him to go to the street to buy them.

Role 1: I understand. You usually don’t take the initiative to ask your husband to buy condoms.

Character 2: Yes. I always told him, I told him to buy a few more boxes at a time and keep them at home, so he doesn’t have to go out all the time.

Role 1: I see. Where do you generally learn about contraception?

Role 2: Knowledge about contraception. I feel that people naturally become aware of this after they get married.

Role 1: You didn’t take the initiative to learn, right?

Role 2: No, because if you don't want to get pregnant, because when you go to the factory, when you were in Foxconn, Foxconn was more humane. There was a place for condoms at the entrance of their factory. I also learned about it at that time, and after getting married, I said that I would definitely prevent it.

Role 1: I understand. Maybe I have seen condoms being distributed in my previous work, so I have some understanding of it.

Character 2: Yes, because that factory was better, they had a special place to store condoms. At that time, that factory might have been preventing AIDS or something like that, but since they had that measure, everyone must have known something about it.

Character 1: Yes. So when you use condoms, do you also consider preventing the spread of diseases?

Role 2: No, mainly because we both had a physical examination when we got married. We all had a physical examination before we got married. I was sure that as long as he didn't cheat and I didn't cheat, it was just the two of us, right? Generally, there wouldn't be any serious illness. I don't think there would be any minor illnesses. Women would definitely have some gynecological examinations, right? I mainly did it to prevent pregnancy, nothing else.

Role 1: Yes, have you learned some relevant knowledge from the Internet, for example?

Character 2: I haven't searched for these.

Role 1: So it was mainly because I just seemed to know it naturally, and also because I had seen some family planning products in my previous work, that's it.

Character 2: Yes, I rarely search these things online.

DOL_056:

Role 1: Next, I would like to ask you something about reproductive health. You just mentioned that when you and your wife were pregnant with your first and second sons, you didn't plan on it, it was all an unexpected surprise. Have you taken any contraceptive measures?

Character 2: Yes.

Role 1: What approach is generally taken?

Role 2: It’s a condom.

Role 1: Condoms. Anything else?

Character 2: Nothing else.

Role 1: No. For those who mainly use condoms, do you use them every time? Or? Occasionally.

Role 2: Use it occasionally.

Character 1: Mainly because you’re worried about an unexpected pregnancy, right?

Character 2: Yes, anyway, if you say no, wouldn’t that hurt the woman?

Role 1: Feels that having an abortion would be bad for the woman’s health.

Character 2: Yes.

Role 1: Have you ever worried about the spread of sexually transmitted diseases, for example, when using condoms?

Character 2: No, there's something going on between you two, right? It's not like you're fooling around outside or anything.

Role 1: I understand. During your sexual intercourse with your wife, does she actively ask you to use condoms because she is worried about pregnancy?

Role 2: Yes.

Role 1: If you have a disagreement on this matter, how do you usually deal with it?

Role 2: There is no disagreement, I just consider her anyway.

Role 1: Understand that if she brings it up, you will cooperate.

Role 2: I would not cooperate even if she didn’t bring it up because I’m also afraid of the child.

Character 1: Understand that having another child is very costly.

Character 2: Yes, you are a girl now, and the expenses for your schooling are too high.

Role 1: Even girls are not cheap. Generally speaking, do you and your wife have different opinions when you decide to use this contraceptive method?

Character 2: No. Usually not.

Role 1: Generally, no. If you are willing to use it, both parties are willing to use it, and if you are not willing to use it, both parties have reached an agreement.

Character 2: Yes.

Role 1: Got it. Where do you usually learn about contraception?

Role 2: From the Internet.

Role 1: Search for some relevant contraceptive knowledge on the Internet.

Character 2: Take a look.

Role 1: Have you talked to, for example, more experienced elders at home, or experienced friends, or professional medical staff?

Role 2: I don’t know how to talk. What can you talk about if it’s just the two people in this family?

Role 1: I haven’t told my family or friends. Have you consulted any professionals?

Role 2: It’s different now than it was before. There were no mobile phones or anything like that. Now everyone has a mobile phone. If you don’t understand something, you can go online.

Role 1: It is indeed very convenient to obtain information. Do you think the information you learn online, that is, the knowledge related to contraception, is helpful in your actual life?

Character 2: Maybe she is pregnant. Some people say you can’t get an IUD or something, and some of them say it’s harmful or something.

Role 1: Sometimes you see some information about the dangers of having an IUD inserted in women on the Internet, so it actually provides some guidance for you in choosing what contraceptive measures to take.

Character 2: Yes.

DOL_057:

Role 1: This may be some issues related to children. Because you just said that you got pregnant with your son by accident because your daughter was still young. I would like to know whether you and your wife have ever actively considered taking contraceptive measures?

Character 2: What do you think it will be like this after you have two children?

Character 1: All along, since I started having sex?

Character 2: No.

Role 1: Never took any contraceptive measures.

Character 2: Yes.

Role 1: During your sexual life, does your wife ask you to use condoms because she is worried about pregnancy?

Character 2: Not this.

Role 1: Do you have a consistent view on whether to use contraception during the entire sexual process? Or do you have different opinions?

Role 2: More consistent.

Character 1: So now maybe both parties really want to have another child, so they are not taking any active contraceptive measures, right?

Character 2: Yes.

Role 1: Do you know what specific methods of contraception are available?

Role 2: I don't know anything else about these except taking some medicine. Because in my hometown, people usually get an IUD.

Role 1: Mainly IUD and oral contraceptives?

Role 2: In our hometown, the upper links are usually the most involved.

Character 1: Do you mean to get an IUD inserted after you have given birth to the desired number of children?

Character 2: Yes.

Role 1: Where did you learn about the two types of contraceptives, IUD and birth control pills? For example, did you learn about these specific methods from some experienced elders in your hometown, or from your friends or family planning medical staff?

Role 2: Specifically, I listen to what the elderly say at home, and sometimes they also talk in the hospital.

Role 1: I’ve heard some of it from the elderly, and maybe I’ve also heard some of it in the hospital.

Character 2: Yes.

Role 1: Do you think that learning about contraception from different channels, such as hospitals and elders around you, is helpful to you? Is it helpful for you to choose whether to use contraception?

Role 2: I didn’t have much of a choice at the time. I thought it was too troublesome to get an IUD, so I didn’t think about it. I didn’t think about it, and neither did my wife. After we had two children, I never thought about getting an IUD.

Character 1: Of course you did mention the issue of the possible stage, because you really do want to have another child.

Character 2: Yes.

DOL_058:

Role 1: What were your thoughts at that time?

Role 2: At that time I just thought I should just let it go.

Role 1: So I didn’t take any contraceptive measures. I thought my parents also wanted grandchildren, so I just let nature take its course.

Role 2: No action was taken, and no action was taken later. It was there when we got married.

Character 1: When we got married?

Character 2: Yes.

Character 1: Oh, we took contraceptive measures when we got married.

Character 2: Yes.

Character 1: So after his parents urged him to have the baby, you decided to let nature take its course and not take any action.

Character 2: Yes.

Role 1: I understand. That does seem to be the case. Finally, I would like to ask some questions about reproductive health. You just mentioned that you and your husband took contraceptive measures in the beginning when you were together and did not plan to have children. What kind of contraceptive methods did you mainly take at that time?

Role 2: Condom.

Character 1: Condoms. Have you ever had anything else?

Role 2: Outside the body.

Role 1: In vitro, were these contraceptive measures taken at the time mainly due to concerns about pregnancy?

Character 2: Yeah.

Role 1: Have you considered, for example, the spread of sexually transmitted diseases?

Character 2: No.

Character 1: OK, I understand. So after having the baby, do you still take contraceptive measures?

Role 2: Yes.

Character 1: Is it the same method? Or are there other contraceptive methods?

Character 2: I have an IUD, so does that count?

Character 1: Yeah. The IUD is an intrauterine device, yes.

Character 2: Yeah.

Role 1: I understand. For example, before getting an IUD, especially during sex, did you ask him to use condoms because you were worried about getting pregnant?

Role 2: Yes.

Role 1: Would you take the initiative to ask him to use it?

Character 2: Yes, because if I’m still pregnant I can’t afford to support her.

Character 1: Yes. Is there a time when he doesn't want to?

Character 2: That’s not bad, no.

Role 1: Understand. Generally speaking, when deciding on which contraceptive method to use, such as whether to use an external contraceptive device or a condom, including your decision to get an IUD, who usually makes the main decision on these contraceptive methods?

Role 2: I got the IUD inserted myself, and it was his own decision.

Character 1: Does that mean he is the one who usually decides the other two?

Character 2: Yes.

Role 1: Have you ever had different opinions on what kind of contraceptive method to use?

Character 2: No.

Role 1: For example, your husband prefers to have external ejaculation, but you prefer to use condoms. Has this situation ever happened?

Character 2: No.

Role 1: Understand. Generally speaking, there are a series of different contraceptive measures, such as condoms, IUDs, IUDs, and in vitro fertilization, etc. Where do you usually learn about these contraceptive knowledge?

Role 2: I went to the hospital to get the IUD inserted. The doctor told me about it. I don’t know about the other two. Anyway, I don’t know. Anyway, this is what happened after I was with him.

Role 1: He told you that you can use these two options.

Character 2: Yeah.

Role 1: Or did you learn some relevant knowledge from the Internet before?

Character 2: No.

Role 1: Have you talked about how to use contraception with, for example, family members, experienced elders at home, or experienced friends?

Character 2: I don’t even feel comfortable saying that.

Character 1: Does this mean no?

Character 2: Yes.

Role 1: OK, I see. Do you think that the contraceptive method of IUD that you heard about from the doctor in the hospital before is helpful to you?

Character 2: I think it helps.

Role 1: Where does the main help come from?

Role 2: I can't get pregnant. I don't want children.

Role 1: You don't want to have another child, and you know there's something clear that can help you, right?

Character 2: Yes.

DOL_059:

Role 1: As mentioned earlier, both children were conceived unexpectedly without any preparation. Have you taken any contraceptive measures?

Character 2: Yes, definitely.

Role 1: What are the main methods you have adopted?

Character 2: For this thing, just buy condoms.

Character 1: Condom. Anything else?

Role 2: Birth control pills, that’s it, before we got married.

Role 1: This is the kind of safety measure that is taken before marriage. Is the contraceptive pill an emergency contraceptive, or does it need to be taken all the time?

Role 2: Emergency contraceptive pills.

Character 1: Got it. Anything else?

Role 2: I got an IUD after we got married, and I definitely won’t have another one since you already have two children.

Role 1: So you mean using an IUD after having two children?

Character 2: Yes.

Role 1: Before having two children, that is, after getting married, before having two children, will you take the initiative to use contraception?

Role 2: Even after marriage, that happens.

Role 1: What are the main considerations for adopting contraception during a process like this?

Role 2: Now that you have a child, you definitely don’t want to have another one.

Role 1: Still out of consideration of not wanting to get pregnant.

Character 2: Yes.

Role 1: During this process, generally speaking, how often do you use contraceptive methods?

Role 2: Generally, we don’t spend much time together after we get married, and we may go back to our hometown occasionally.

Role 1: It is less common, so contraceptive measures are less often taken.

Role 2: Yes, you are working outside and we live apart.

Role 1: Yes, does your wife ask you to use a condom during sex because she is worried about pregnancy?

Character 2: That will definitely be a requirement.

Role 1: If you have a disagreement, how do you usually deal with it?

Role 2: I have had disagreements, but they are rare. I am a man, so you know what I mean. Sometimes I don’t wear a condom, but it’s rare because I don’t want to get pregnant.

Role 1: If there is a disagreement, which approach will be more likely in the end, for example, not wearing condoms more often or wearing condoms more often?

Character 2: Of course we should wear condoms more often. We don't want to get pregnant.

Role 1: So you will still discuss it during the process.

Role 2: This thing will be discussed.

Role 1: Who generally makes the final decision? Who is the main person who makes the decision on, for example, using condoms? And you just mentioned taking birth control pills, and now deciding to get an IUD? Who is the main person who makes the decision on these contraceptive methods?

Role 2: It must have been me who said it, but she agreed. If she didn’t agree, there would be no way to do it.

Character 1: You mentioned it, but she also approved of it.

Role 2: Yes, if she doesn’t agree, then you can’t get the IUD.

Character 1: Yes. Did you ever have any disagreements about the specific method of contraception?

Role 2: Very rarely. Sometimes, how should I put it, sometimes we don’t wear condoms, but it’s very rare. It’s very rare in this situation. The rest are okay. There are no major differences.

Role 1: Got it. Where do you usually learn about contraception?

Role 2: I haven’t understood it.

Role 1: Never took the initiative to find out?

Role 2: Aren’t these all like this? Contraceptive pills, condoms. Sometimes I hear people say that maybe they get an IUD after they get married and have kids.

Role 1: Maybe you have heard it from some friends around you who have had experience.

Character 2: Occasionally, yes.

Role 1: Have you discussed these things with, for example, professional doctors and nurses?

Character 2: No.

Role 1: With other family members, such as those who already have experience.

Character 2: No.

Role 1: What about the information on the Internet?

Role 2: You don’t need to go online for this. If you don’t want a child, you can just get an IUD, right? There shouldn’t be anything special about this. Anyway, I haven’t looked into it.

Role 1: I never took the initiative to learn about it. It seemed like I just heard about it from others and then I knew it naturally.

Character 2: Yeah. Yes.

DOL_060:

Role 1: Have you and your wife ever taken any proactive contraceptive measures?

Character 2: It happened only in the first two years of marriage, and then it disappeared.

Role 1: What was the main method of contraception you used in the first two years?

Role 2: Use normal condoms for contraception.

Character 1: Is there another way?

Character 2: No.

Character 1: What was the main reason for using contraception at that time?

Role 2: I just think that I am young and want to live a life as a couple. I don’t want to have children so early and I like to play.

Role 1: Because they are still relatively young, they are not ready to be parents.

Role 2: Yes, the key thing was that I was still a child at that time, and suddenly I wanted to have a child.

Character 1: Yeah, not ready yet.

Character 2: Right.

Role 1: During this process, in addition to considering pregnancy, were you worried about the spread of sexually transmitted diseases and used contraceptive measures?

Character 2: No.

Role 1: During sexual intercourse between you and your wife, will she ask you to use a condom because she is worried about pregnancy?

Role 2: She didn't ask for it. She didn't care, because we both thought we wouldn't have it for the next two years, and if it did happen, it wouldn't matter, that's the situation.

Character 1: I see.

Role 2: I was more casual at that time.

Character 1: Yes. Do you two usually disagree on who makes the final decision on whether to use condoms?

Character 2: No.

Role 1: When you say you want to wear it, she usually says she wants to wear it, and when she says she wants to wear it, you usually agree.

Character 2: Yes.

Role 1: Where do you usually learn about contraception?

Role 2: It is the mobile network.

Role 1: On the Internet. Have you ever discussed these related matters with some experienced family members and elders around you?

Character 2: No.

Role 1: What about friends? For example, more experienced friends.

Character 2: Neither.

Character 1: No problem. I was just asking if you've talked to the doctor or nurse about contraception?

Character 2: No.

Role 1: Got it. So it was mainly some knowledge about contraception that I collected online.

Character 2: Yes.

DOL_061:

Role 1: I understand. Next, you might ask some questions about reproductive health. I want to know if you have ever taken any active contraceptive measures?

Role 2: Take, don’t be a child, take.

Role 1: If you don’t want to have children, then you can adopt this approach. Wasn’t there any such approach before?

Character 2: Yes.

Role 1: Yes, what methods do you generally use to prevent pregnancy?

Role 2: I have taken birth control pills and used condoms, and I don’t want to have children anymore, so I plan to wait a little longer before getting an IUD.

Character 1: Are the contraceptive pills you just mentioned the emergency ones, or the ones that you have to take all the time?

Role 2: Emergency contraceptive pills.

Role 1: In addition to condoms, birth control pills, and the possibility of getting an IUD, have you used other methods? For example, safe period, and ejaculation outside the vagina?

Role 2: There is also less ejaculation outside the body.

Role 1: Have you considered the safe period?

Character 2: Yeah.

Role 1: Understand, is the main reason for using these contraceptive measures due to concerns about unplanned pregnancy?

Character 2: Yeah.

Role 1: Have you ever considered, for example, the spread of sexually transmitted diseases and how to prevent the spread of sexually transmitted diseases?

Character 2: Not really.

Role 1: I see. Would you ask your husband to use condoms because you are worried about pregnancy?

Role 2: Yes.

Role 1: Will you have any disagreements on this matter?

Role 2: Disagreement?

Character 1: Yeah, let’s say you want him to use a condom, but he doesn’t want to?

Character 2: Not yet.

Role 1: There is none here, right? Do you use condoms most of the time, or for example, since you just mentioned condoms, emergency contraception pills, two different types, and withdrawal, these three different contraceptive methods, which one do you use most frequently?

Role 2: Condom.

Role 1: Do you basically use condoms every time?

Character 2: Yes.

Role 1: Generally speaking, when deciding, for example, whether to use condoms, emergency contraceptive pills, or ejaculation outside the vagina, or if you don't plan to have a baby now and want to consider getting an IUD, who usually makes the decision between you and your husband about which method of contraception to use?

Role 2: We both decided and discussed it.

Role 1: Who usually brings it up first?

Role 2: He.

Role 1: If he were to raise the issue, which one would he raise?

Role 2: The ring.

Character 1: Is it time to put on the ring now?

Character 2: Yeah.

Character 1: What happened before?

Character 2: I was like that before when I didn’t want children.

Role 1: What do you do when you want a child?

Character 2: It's all the same.

Role 1: It was the same as before, using condoms, withdrawal, or birth control pills?

Role 2: Condom.

Character 1: Mainly condoms, right? For example, if you decide to use emergency contraceptive pills, who usually makes the decision?

Role 2: Me.

Role 1: Did you decide to use the emergency contraceptive pill after you failed to use a condom, or did you decide to use the pill before you had sex?

Character 2: These things belong to both of us.

Character 1: You two are going to discuss this together, right?

Character 2: Yeah.

Role 1: I probably have two more questions here, and I have another question. Where do you generally learn about contraception?

Role 2: I’m old now, I know everything, I don’t need to understand anymore.

Role 1: I don’t need to know. Do you feel like you can learn these things naturally from the Internet? Or is it not from the Internet?

Role 2: I know everything from what I see online and through experience.

Role 1: Have you talked about this with some experienced family members, elders or friends around you?

Character 2: Yes, friends sometimes say that.

Role 1: Have you talked to, for example, doctors and nurses?

Character 2: No.

Character 1: Not this.

Role 2: It’s a simple question, why are you asking people?

Role 1: Yes, because there are some family planning offices or hospitals that promote contraceptive knowledge or distribute family planning supplies. Do you think talking to your friends about this helps you understand contraception now?

Character 2: Yes.

DOL_062:

Role 1: OK. Next, I would like to ask some questions related to reproductive health. Since you and your husband met, have you ever taken any contraceptive measures?

Role 2: Yes, before I got married.

Role 1: What kind of contraceptive measures are mainly taken before marriage?

Role 2: Just use a condom.

Role 1: Have you tried anything else besides this?

Character 2: No.

Role 1: For example, have you considered things like the safe period, emergency contraceptive pills, short-acting contraceptive pills, and ejaculation outside the body?

Character 2: No.

Role 1: What was the main reason for using contraception at that time?

Role 2: Because I was not married before, I had to do that. Otherwise, what if I got pregnant?

Role 1: Mainly worried about unexpected pregnancy.

Character 2: Yes.

Role 1: Have you ever considered worrying about the spread of sexually transmitted diseases?

Role 2: Generally speaking, no, I don’t think too much about it.

Role 1: You would ask your husband to use condoms before you got married because you were worried about getting pregnant.

Role 2: Yes.

Role 1: You would take the initiative to ask him if there was a time when you wanted him to use a condom but he didn't want to.

PERSON 2: Maybe once in a while.

Role 1: If this happens, what would you usually do?

Character 2: What should I do? I just talked about it, and in the end he still listened to me.

Role 1: There will be some disagreements at the beginning, but if you go on, he will still agree with you. Generally, when choosing which method of contraception to use, who makes the decision among you?

Role 2: Me.

Role 1: Mainly you, your husband will suggest other contraceptive methods, for example.

Character 2: No.

Role 1: So you haven't used any contraceptive methods since you got married, but you only used condoms before you got married. Where did you learn about contraception?

Role 2: We all know it naturally and need to understand it.

Character 1: It feels like when you get older and grow up, you know everything.

Character 2: Adults must be so developed now, right? So open.

Character 1: That’s because there is a lot of information on the Internet, and then I naturally learned about it from the Internet.

Character 2: Yes.

Role 1: Have you discussed these issues with your family members or friends who have experience with contraception? About how to use contraception?

Character 2: No, I just wear this and that, nothing else. Taking medicine is not good for the body.

Role 1: Actually, I have heard about this method, for example, taking birth control pills, but I have never tried it because I think it is not good for my health.

Character 2: Yes.

Role 1: Have you ever talked to some professional doctors or nurses about how to use contraception?

Character 2: No.

DOL_063:

Role 1: I understand. Have you and your wife ever taken any contraceptive measures?

Role 2: Yes.

Role 1: What approach is generally used?

Role 2: Is it commonly used?

Character 1: Yeah.

Role 2: Include some time in it, it’s the same thing anyway, so pay some attention to it.

Role 1: Safe period, will consider the safe period.

Role 2: Yes, including some useful things, anyway, this is indispensable.

Role 1: Some commonly used contraceptive measures may include condoms, safe period, or intrauterine devices, such as IUDs, birth control pills, withdrawal, etc. Which ones do you use most often?

Role 2: There are three.

Role 1: Three.

Role 2: safe period, condoms, and in vitro fertilization.

Role 1: I see. These three are the most commonly used. Are there any others that I haven't mentioned but you also use frequently?

Character 2: No, these three, basically just these three.

Role 1: OK, I understand. How often do you take contraceptive measures? Do you take it every time or occasionally?

Character 2: Every time, basically.

Role 1: Is the main reason for taking contraceptive measures because of the fear of unwanted pregnancy?

Character 2: Yes.

Role 1: Have you considered preventing the spread of sexually transmitted diseases?

Character 2: I don’t really have that.

Role 1: Understand.

Role 2: Husband and wife always have hygiene concerns before having sex, I have never considered this.

Role 1: Understand. Does your wife ask you to use condoms because she is worried about pregnancy?

Character 2: No, she wants to.

Role 1: I understand.

Role 2: Because this is after the third child, no, it is after the second child and when you are preparing to have the third child, the probability is basically like this.

Role 1: Right before this, she wouldn't ask you to wear a condom because she wants the baby herself.

Character 2: Yes.

Role 1: Understand. Have you ever had a disagreement on this matter? For example, she wants you to use a condom but you don't want to, or you want to use it but she doesn't want to.

Character 2: No.

Role 1: Understand. Who between you and your wife usually makes the main decision about which contraceptive method to use?

Role 2: Both of them are very what? Very natural, anyway, there are only these three, your safe period, you can also ignore it. The latter two are very natural anyway, there is no decision or no decision.

Role 1: Is there a certain method of contraception that one of you would prefer? For example, which one would you prefer, or which one would your wife prefer?

Role 2: In vitro.

Character 1: This is the option you prefer, right?

Role 2: Yes, both of them are inclined towards that.

Role 1: Both of us prefer this, I see. Where did you learn about contraception in general?

Character 2: I feel like I learned this thing by myself, and I don’t know where I learned it.

Character 1: No impression, right?

Role 2: I have no impression.

Role 1: Will you learn some relevant knowledge from the Internet?

Role 2: Also popularized.

Role 1: Have you ever discussed with some experienced family members or friends about how to use contraception?

Role 2: I talked to my friends, but not my family.

Role 1: Like professional medical staff?

Character 2: No.

Role 1: Do you think talking to your friends about these things helps you take contraceptive measures?

Role 2: That's helpful.

Character 1: How do I put it?

Role 2: Communication, right? People may learn something from each other when we communicate about what they know but we don’t. So I think it’s useful.

DOL64:

Interviewer: I understand. Next, I would like to talk about some issues related to reproductive health. Have you and your wife taken the initiative to take contraceptive measures, including before marriage until now?

Interviewee: What?

Interviewer: Do you take the initiative to take contraceptive measures? Since you met me,

Respondent: I have always used contraceptive measures.

Interviewer: There always is.

Interviewee: Yes, now the first child is an accident, and an unexpected pregnancy is also an accident, but it is very accurate, you know what I mean? There was a time when I didn't take any measures and it happened.

Interviewer: Actually, you pay attention every time, but you get pregnant once you don’t pay attention.

Respondent: Yes, I got it. The first one, the second one. I didn't take any medicine, I just used contraceptive measures myself.

Interviewer: Do you use condoms or something else?

Respondent: Wear a condom.

Interviewer: Besides condoms, have you taken any other contraceptive measures?

Respondent: I don’t understand the others, there is nothing else, I don’t know the others.

Interviewer: For example, some of the things that people often mention, including calculating the safe period for girls, or some people choose to use IUDs or oral contraceptives, and boys have ejaculation outside the body, etc. Have you ever taken such measures?

Respondent: No, none of that. I put on the condom unilaterally.

Interviewer: I see. During this long period of time, was the main reason for you to take contraceptive measures because you were worried about unwanted pregnancy?

Interviewer: Yes.

Interviewer: I was worried.

Respondent: I'm not worried about that. I personally prefer it this way and like to wear a condom for a longer time.

Interviewer: I see. Have you ever thought about, for example, preventing the spread of sexually transmitted diseases?

Interviewee: My husband and I have never thought about diseases. Is it really possible? I don’t think so.

Interviewer: Although contraceptive measures are called contraceptive measures, in addition to preventing pregnancy, they also have the function of preventing the spread of sexually transmitted diseases. So I would like to ask what you did at that time?

Respondent: I have never thought about the possibility of sexually transmitted diseases. I am simply afraid of an unexpected pregnancy.

Interviewer: I understand.

Respondent: I haven’t paid enough attention or consideration to this aspect yet.

Interviewer: I understand. Does your wife ask you to use condoms because she is worried about pregnancy?

Respondent: No, she didn’t ask for it. It was all my own initiative.

Interviewer: Do you have disagreements on whether to use condoms? For example, you want to use it but your wife doesn't want to, or your wife doesn't want to use it but your wife wants to use it but you don't want to?

Respondent: There is no disagreement on this. If I don't use a condom, I will ejaculate outside the body.

Interviewer: I see. In fact, your wife and you will not disagree on this matter.

Respondent: I don't disagree. I usually prepare this. Yes.

Interviewer: I understand.

Interviewee: I wasn't prepared, so I probably just drank some wine.

Interviewer: I understand. Who usually makes the decision on contraceptive measures? For example, do you use condoms or use external genitalia?

Respondent: I am the one who makes the decision about whether to use a condom.

Interviewer: Were there any disagreements on which approach to take?

Interviewee: There is no disagreement. Basically, it is my decision whether to use a condom or not, and it is also my decision whether to ejaculate outside the body. For example, the last time we had sex, I ejaculated outside the body.

Interviewer: I see. Where do you usually get information about contraception?

Interviewee: I've been in a relationship before and I know about this.

Interviewer: How did you first learn about it?

Interviewee: How did you know that and how would I remember it? This is common sense.

Interviewer: Do you think you will understand it naturally when you get older?

Respondent: Yes, when you reach a certain age, you will know how to take contraceptive measures.

Interviewer: Do you learn about contraceptive measures from websites, for example?

Respondent: No. There is no information on the website about contraceptive measures. You will know it yourself after a long time. No one teaches you this.

Interviewer: Do you usually talk to experienced friends, family members, or professional medical staff about how to prevent pregnancy? How to prevent pregnancy?

Interviewee: No, I don’t want to talk about it.

Interviewer: I see.

Interviewee: I haven't discussed these contraceptive issues. Yes, I talked to my wife. To put it bluntly, I went out to visit prostitutes, so I thought of taking those contraceptive measures. My family members couldn't say that they didn't think about it. They pay more attention to this.

Interviewer: I understand, especially if you want a second baby.

Respondent: Yes, but we have been using contraception recently.

Interviewer: What are the considerations behind this?

Interviewee: First, we also want to have a plan. Next year is the Year of the Dragon, and we want to have a dragon baby. Yes, that's the first reason. What's the second reason? We have this habit.

DOL_065:

Interviewer: I see. You just mentioned that both children were born unexpectedly. Have you and your wife taken any contraceptive measures since you met?

Interviewer: I did have it after I had my first child, but it was an accident.

Interviewer: Yes, after you gave birth to a baby, what kind of contraceptive methods have you adopted? What contraceptive methods are there?

Interviewee: How should I put this?

Interviewer: Generally speaking, we know some contraceptive measures, including the use of condoms, or the calculation of the safe period for girls, intrauterine contraceptive rings, birth control rings, or short-acting contraceptives for girls, and external ejaculation for boys, etc. Have you ever used these methods?

Interviewee: I have tried everything except IUD and birth control pills.

Interviewer: I understand. Was the main reason for trying these contraceptive measures at the time because you were worried about unwanted pregnancy?

Interviewee: Yes.

Interviewer: Have you ever considered worrying about the spread of sexually transmitted diseases?

Interviewee: No, they are an old couple.

Interviewer: Well, I haven’t thought about it. After you had your first baby and started to try active contraception, did you generally use contraception every time, or only occasionally?

Interviewee: Occasionally.

Interviewer: What is the approximate frequency?

Respondent: The frequency is probably that no measures are often taken.

Interviewer: I understand. Does your wife ask you to use condoms because she is worried about pregnancy?

Respondent: No, she would actively ask me not to use it.

Interviewer: She actively asked you not to use it. Does that mean you want to use condoms but she doesn't?

Interviewer: I am worried that there will be too many accidents.

Interviewer: If there is a disagreement on whether to use condoms, how do you handle it?

Interviewee: There will be no disagreement.

Interviewer: Will you reach a consensus in the end?

Interviewee: Yes.

Interviewer: More of using or not using?

Respondent: It should be used less frequently during the safe period, but is basically used during the dangerous period.

Interviewer: Regarding specific contraceptive methods, such as the three methods you just mentioned, such as condoms, safe period, and withdrawal, who usually makes the main decision between you and your wife on which one to choose?

Respondent: It's quite casual, no one makes the decision. If it's the safe period, no one needs to make the decision. Both people will agree to use condoms.

Interviewer: I understand. If it is not a safe period, there may be some disagreements in the discussion process?

Respondent: There is no disagreement during the non-safe period, so basically it is not used.

Interviewer: I understand. Okay. Where do you usually get information about contraception?

Interviewee: I think this should be common sense now.

Interviewer: It seems like everyone just knows it naturally.

Interviewee: Yes.

Interviewer: Do you go online to learn some relevant knowledge and information?

Interviewee: I should have learned about it online when I was a kid, but now I’m more mature.

Interviewer: Can you still recall how you first learned more about the relevant knowledge?

Interviewee: That’s on the Internet.

Interviewer: Based on the Internet, have you discussed this with family members or friends who have more experience with contraception?

Interviewee: No.

Interviewer: What about professional medical staff?

Interviewer: My wife studied medicine.

Interviewer: So her knowledge about contraception, is she helping you to improve your knowledge in this area?

Interviewee: Yes.

Interviewer: I see. Do you think that after talking with your wife, since she also studied medicine and has some knowledge about this, has it helped you understand contraception?

Interviewee: It’s not very helpful. I think no matter how safe this thing is, accidents can still happen.

Interviewer: I see. So what helped you the most in understanding contraception knowledge was learning from the Internet?

Interviewee: Yes.

DOL_066:

Role 1: I see. Next, I might like to talk about some things related to reproductive health. I would like to ask if you and your wife have ever taken any contraceptive measures?

Role 2: That depends on you, right? When you ask your child to do something, you mean you have a plan, right? If you have a plan, and you take appropriate measures, that's normal.

Role 1: When you are planning to have a baby, you may not take contraceptive measures, but you will take them at other times?

Character 2: Yes.

Role 1: Understand.

Character 2: Yes.

Role 1: What kind of contraceptive methods do you usually use? For example, people often mention condoms, the safe period for girls, intrauterine devices, emergency contraceptive pills, short-acting contraceptive pills, and ejaculation outside the body for boys, etc.?

Role 2: I just need to take some measures, any normal condom will do.

Role 1: Mainly condoms.

Role 2: Yes. (inaudible 01:03:07) measures.

Role 1: Have you considered other options? Other contraceptive measures.

Role 2: Have you considered anything else?

Role 1: In addition to worrying about unwanted pregnancy, have you ever been concerned about, for example, using contraceptive measures to prevent the spread of sexually transmitted diseases?

Character 2: No.

Role 1: Does your wife ask you to use condoms because she is worried about pregnancy?

Role 2: If you don't want children, you should take contraceptive measures.

Role 1: Will she ask you to use a condom?

Role 2: We have discussed it, there is no need to be too proactive, just take safety measures.

Role 1: Do you two have disagreements about whether to use condoms? For example, she wants you to use a condom but you don't want to, or you want to use a condom but she doesn't want to.

Role 2: We couldn't say no before, we couldn't say no, and we couldn't say it was too much, so we finally discussed it. For example, let's focus on condoms.

Role 1: Sometimes there may be some small disagreements, but in the end we will still reach an agreement and use condoms?

Character 2: Yes.

Role 1: How do you usually discuss this process?

Role 2: If you don't want a child, just take safety measures. It will be good for her as well as help prevent disease.

Role 1: Can you talk about some of the consequences of not adopting safety measures?

Character 2: Yes.

Character 1: Do you usually talk more about this, or does your wife talk more about it?

Role 2: All discussed.

Role 1: Both. Who usually chooses and decides whether to use a condom?

Role 2: Half each.

Role 1: Half and half. I would like to ask where do you generally learn about contraception?

Role 2: Search on Baidu and the Internet.

Role 1: Online. For example, talk to experienced family members or friends about how to prevent pregnancy?

Role 2: Sometimes I say it out loud when I'm joking with my friends. I don't usually talk about it seriously, I just say it in a joking way (inaudible 01:06:24), which is normal.

Role 1: During this joking process, do you think the things you talked about related to contraception were helpful in your later process of taking contraceptive measures?

Role 2: Yes, as long as you are healthy, that's good. Isn't that good? I also read the news. I have a colleague who doesn't have any good measures, and then they (inaudible 01:06:57).

Role 1: I do have relevant experience, around my friends.

Role 2: Yes, a colleague of ours may have discussed this before, but no appropriate measures were taken. I forgot what disease his wife had, so she went to see a doctor.

Character 1: In short, there was a consequence. My wife did not take any contraceptive measures and became sick.

Role 2: Yes. Then I went to Zhengzhou for medical treatment.

Role 1: Understand. Have you ever talked to a professional doctor or nurse about contraception?

Character 2: I don’t really have that.

Character 1: No, okay. I have one last one.

Role 2: You say.

Role 1: Please say what you wanted to say first.

Role 2: I usually go see some doctors, get medical treatment or do something else and listen to what the doctors say.

Role 1: Understand, but don’t take the initiative to talk. However, during the process of seeing a doctor, you will get some popular science knowledge from medical staff.

Role 2: Yes. People have heard about those defensive measures, and what diseases they might get if they don’t take them. They have heard about that.

Character 1: Are these helpful?

Role 2: It’s okay, I can’t say there is none.

DOL_067:

Interviewer: I would also like to know, since you and your husband met, have you taken the initiative to take contraceptive measures during your sexual life?

Interviewee: Yes, I will. Because I have two children, I have to take measures.

Interviewer: I see. So did you start taking this approach after you had your two babies? Or did you do it before that?

Interviewer: I took action after giving birth to my first child.

Interviewer: I understand. What kind of contraceptive methods do you usually use?

Interviewee: IUD insertion.

Interviewer: Did you get on right after the first one?

Interviewee: Yes, I put it on when I had my first child, and took it off when I had my second child.

Interviewer: At that time, I was planning to have a second child, so I had the IUD removed.

Interviewee: Yes.

Interviewer: I see. Have you used other safety measures besides IUD? For example, condoms, and the safe period for girls. Do you mean you have used condoms?

Interviewee: Yes.

Interviewer: What about others?

Interviewee: Nothing else.

Interviewer: For example, have you ever tried things like birth control pills, safe periods, or ejaculation outside the body for men?

Interviewer: It was taken in vitro.

Interviewer: It’s mainly about the safe period, IUD insertion and in vitro fertilization.

Interviewee: Yes.

Interviewer: I understand. When you take safety measures, are you mainly worried about unwanted pregnancy?

Interviewee: Worried.

Interviewer: Do you take any safety measures because you are worried about the spread of sexually transmitted diseases?

Interviewee: No.

Interviewer: During your sexual life, would you actively ask your husband to use condoms because you are worried about pregnancy?

Interviewee: Yes.

Interviewer: Were there any disagreements on this matter?

Interviewee: No.

Interviewer: Have you ever been in a situation where you want him to wear a condom but he may not be willing to do so at first?

Interviewee: It has happened.

Interviewer: If this situation occurs, how will you negotiate and deal with it?

Interviewee: In the end, they still have to ask for my opinion.

Interviewer: I will listen to you in the end.

Interviewee: Yes.

Interviewer: How would you convince him?

Interviewee: I told him that if I got pregnant and couldn’t have the baby, I would suffer and would have health problems.

Interviewer: I see. Usually, you two can reach an agreement in the end.

Interviewee: Yes.

Interviewer: You just mentioned that you had an IUD inserted after giving birth to your first baby. Who was the main person who made the decision at that time?

Interviewee: Me.

Interviewer: Did your husband have different opinions on this decision at that time?

Interviewee: No, we are the same.

Interviewer: I see. Apart from IUDs, if you choose to use condoms or have insemination outside the body, who usually decides?

Interviewee: Me.

Interviewer: It's also your decision. Do you have different opinions? For example, your husband may prefer to use a certain safety measure, while you prefer another one?

Interviewee: My husband prefers to have sex outside the body, and I will make him wear a condom, that's it.

Interviewer: I understand. If you have different options at this time, how will you negotiate?

Interviewer: I told him and he understood, and it became normal again.

Interviewer: You will also try to persuade him?

Interviewee: Yes, I will convince him.

Interviewer: What are the general reasons for persuasion in this regard?

Interviewee: The persuasive reason is that wearing a condom is safer, as sex outside the body cannot be controlled and may lead to an unwanted pregnancy.

Interviewer: Do you understand? Or will you discuss the risks of pregnancy with your husband?

Interviewee: Yes.

Interviewer: I see. Where do you usually learn about contraception? For example, do you search online?

Interviewee: No.

Interviewer: Where do you usually learn about it?

Interviewer: It should be available on mobile phones.

Interviewer: On the mobile phone, you can also get some network information.

Interviewee: Yes.

Interviewer: Have you discussed this with people around you? For example, have you discussed with experienced family members or more experienced friends about how to prevent pregnancy?

Interviewee: I didn’t hear what you just said.

Interviewer: I would like to ask, have you ever talked to your family or friends who have experience about how to use contraception?

Interviewee: No, we haven’t talked about it.

Interviewer: I understand. Have you talked to any professional doctors or nurses?

Interviewee: Yes.

Interviewer: Under what circumstances did you have this conversation?

Interviewee: After I got pregnant, I didn't want this child. After I gave birth to my daughter, I got pregnant once, but I miscarried the child and didn't want it. The difference was too small, just a few months, and my daughter was still breastfeeding at that time.

Interviewer: That’s right, they were too close.

Interviewee: Yes.

Interviewer: So when you went to the hospital, did you specifically talk to the doctor and the nurse about how to use contraception?

Interviewee: Yes.

Interviewer: Did you decide to get the IUD after talking to them?

Interviewee: Yes.

Interviewer: I see. After communicating with the doctors and nurses, do you think it helps you with the issue of contraception in your future married life?

Interviewee: Yes.

Interviewer: I understand.

Interviewee: It helps.

Interviewer: Yes, I feel more clear about how to prevent pregnancy.

Interviewee: Yes.

Interviewer: Does this help manifest itself in other ways? For example, you feel more independent in the process, or you have a better understanding of some knowledge, or you can communicate better with your husband.

Interviewer: Do you mean after talking to the doctor?

Interviewer: Yes, after talking to the doctor.

Interviewee: I know there are many ways to prevent pregnancy, and I will tell my husband how to do it and how to communicate with him.

Interviewer: I feel like I understand better how to communicate.

Interviewee: Yes.

DOL_068:

Role 1: Finally, I would like to ask some questions about reproductive health. Have you and your wife ever tried active contraceptive measures?

Role 2: Yes.

Role 1: What are the main approaches?

Role 2: with ring.

Character 1: You have an IUD now, right?

Character 2: Yes.

Role 1: Did you take any other safety measures before having the IUD inserted?

Role 2: Condom.

Character 1: Anything else?

Character 2: No more.

Role 1: Worried, I have considered it. Will your wife ask you to use condoms because she is worried about pregnancy?

Character 2: Yes.

Role 1: Do you have some disagreements on this matter? For example, she wants you to use it but you don't want to, or you want to use it but she doesn't want to?

Character 2: Neither.

Role 1: Generally speaking, will you choose to agree when she asks you something?

Character 2: Yes.

Role 1: How would she usually tell you and ask you to wear it?

Role 2: For her health, afraid of accidents.

Role 1: Afraid that an unexpected pregnancy would be bad for her health?

Character 2: Yes.

Role 1: I understand. Who makes the decision on the issue of IUD placement?

Character 2: She decided on her own.

Role 1: Does she want to get an IUD herself?

Character 2: Yes.

Role 1: What do you think?

Role 2: I agree with her. It's good for her, but I'm afraid she might get pregnant unexpectedly. Do you want it or not? Right?

Character 1: Yes. So when she proposed to have an IUD, you were quite supportive?

Character 2: Yes.

Role 1: Yes. What was her main reason for wanting to have an IUD? How did she bring it up with you?

Role 2: It's mainly for health reasons. One child is enough. I'm afraid that if I get pregnant and you say no, your parents will find out and say something. So for safety reasons, I'd rather get an IUD.

Role 1: Understand. In addition to IUDs, who usually decides on the use of condoms as a contraceptive method?

Role 2: Me.

Role 1: Do you have different opinions on this matter?

Character 2: No.

Role 1: If there are different opinions, how do you usually resolve them?

Role 2: Let’s sit together and talk. Ask quickly.

Role 1: Do you usually choose to wear a condom, or do you sometimes wear it and sometimes not?

Role 2: Sometimes I wear it, sometimes I don’t. I don’t wear it during ovulation.

Role 1: You may not wear it during the safe period. Where do you usually learn about contraception, such as condoms, safe period, birth control rings, IUDs, etc.?

Role 2: It seems like my wife knows a lot, and she just talks about these things.

Role 1: Did your wife introduce these to you?

Character 2: Yes.

Role 1: Understand. Do you learn some relevant knowledge from the Internet?

Character 2: Sometimes.

Role 1: Will you then talk to other more experienced family members or friends?

Character 2: No, let’s not talk about this.

Role 1: Yes. Have you talked to professional doctors and nurses about contraception?

Role 2: I don’t really know, and I don’t understand them.

Role 1: Understand. So you mainly go online to learn about relevant knowledge, and your wife knows more and will tell you.

Character 2: Yes.

DOL_069:

Interviewer: I understand. You just mentioned that you had two unexpected pregnancies. I would like to ask you and your husband, have you always taken the initiative to take contraceptive measures?

Interviewee: Yes, because there was an unexpected situation before. At that time, I believed in the safe period and would check the date. But I might have ignored ovulation. Sometimes it happens early, sometimes late. It might be because of this situation, and then it happened unexpectedly.

Interviewer: I see. Did you take any other contraceptive measures besides the safe period?

Interviewee: Yes. Condoms, or outside the body.

Respondent: Yes. Condoms or withdrawal.

Interviewer: Besides condoms, in vitro fertilization and safe period, have you ever taken birth control pills?

Interviewee: I rarely eat this.

Interviewer: I understand.

Interviewee: I have hardly ever eaten it.

Interviewer: OK. So when you take contraceptive measures, is your main concern the possibility of an unwanted pregnancy?

Interviewee: Yes.

Interviewer: Have you ever considered, for example, preventing the spread of sexually transmitted diseases?

Interviewee: Not really. I haven't thought about it that much. After all, I work in a hospital and I know a lot about this aspect. When I talked to him, I went over everything with him.

Interviewer: Just a checkup, a premarital checkup or something like that.

Interviewer: Yes, I examined him, but I didn’t tell him the truth at the time. I just said I was giving him a physical examination.

Interviewer: I understand. It’s quite professional on your side.

Interviewee: Yes.

Interviewer: In your daily life as a couple, do you actively ask your husband to use condoms because you are worried about pregnancy?

Interviewee: Yes. Because I am in my ovulation period these days, I will tell him.

Interviewer: Have you ever encountered a situation where, for example, you want him to use a condom, but he doesn't want to?

Interviewee: No, that's not the case. Anyway, after I told him about it, he was quite cooperative because he probably didn't want any accidents to happen.

Interviewer: I understand. You just mentioned several different methods, including condoms, calculating the safe period, and in vitro fertilization. Do you two have different preferences for specific contraceptive methods?

Interviewer: You mean we care more about which contraceptive method to use?

Interviewer: Yes.

Interviewee: Outside the body.

Interviewer: There will be more outside the body.

Interviewer: Yes, more outside the body.

Interviewer: Is this something you two are relatively consistent on, or do you have some different opinions?

Interviewee: Sometimes it’s different. For example, I want him to wear a condom, but he doesn’t want to, so I say why not have sex outside the vagina.

Interviewer: I understand. How would you discuss this with him? How would you tell your husband about this?

Interviewee: Sometimes, for example, I told him that it was the safe period these few days, and I told him to wear a condom, and then he said he didn’t want to wear a condom, and if he didn’t want to wear a condom, then he would ask questions, that’s it, but (inaudible 01:04:31). There was also an abortion in the middle, and if it happened again, he couldn’t hold his head up, he would be unhappy, and he would feel embarrassed.

Interviewer: He also feels that if he lets you get pregnant unexpectedly again and then has to abort it, he would feel embarrassed?

Interviewee: Yes, he felt very embarrassed, and the elderly in the family would definitely say that we were not paying attention.

Interviewer: So despite some social pressure and family pressure, they generally still listen to your advice and take contraceptive measures. Where did you first learn about contraception? I know you studied medicine, so maybe you also know more about it from professional knowledge?

Interviewer: It’s called contraceptive measures, right?

Interviewer: Yes.

Interviewee: I also check a little on the Internet and combine what he knows with what I know, that’s it.

Interviewer: I understand. In fact, I also search online to learn more about it. Is it mainly for communication between the couple?

Interviewee: Yes.

Interviewer: Do you discuss these things with other more experienced friends or more experienced family members?

Interviewee: No, we haven’t talked about it.

Interviewer: I see. Since you are also a medical worker, do you discuss this with other colleagues, such as those in the obstetrics and gynecology department or those working in family planning?

Interviewee: No. (inaudible 01:06:39), I didn’t really participate.

Interviewer: So your colleagues sometimes talk about it, but you don’t really participate in discussing it?

Interviewee: Yes.

DOL_070:

Role 1: I see. OK, now I want to ask you something about reproductive health. Have you and your husband ever taken any contraceptive measures?

Role 2: Yes.

Role 1: What approach will you take?

Role 2: A very common approach.

Role 1: Generally speaking, for example, people often mention things like condoms, safe period, contraceptive rings, IUDs, birth control pills, and withdrawal, etc. What methods do you usually use?

Role 2: Condom.

Role 1: Are condoms the most commonly used?

Role 2: Yes, basically I haven’t used anything else.

Role 1: Understand. Is the main reason you use condoms because you are worried about unwanted pregnancy?

Character 2: Yes.

Character 1: Were there any other reasons you considered?

Character 2: He must have used something else for other hygiene purposes as well.

Role 1: Consider preventing infectious diseases. For yourself, would you actively ask your husband to use condoms because you are worried about pregnancy?

Role 2: He has requirements, but he is generally more self-conscious.

Role 1: He will generally use it even if you don't mention it. Have you ever had a disagreement on this matter? For example, he doesn't want to use it, but you want him to use it, or you don't want to use it, but he wants to use it?

Character 2: No.

Role 1: Understand. Do you have different opinions on what specific contraceptive methods to use?

Role 2: No, because I have always used condoms and nothing else.

Role 1: Understand. So generally speaking, when choosing whether to use a condom, who usually makes the final decision?

Role 2: It's like this anyway. No decision has been made because after all, they are children. You can't cause unnecessary trouble because of this.

Role 1: Everyone knows that if you don’t use contraception there will be consequences and troubles, so everyone is relatively unanimous in their opinions.

Character 2: Yes.

Role 1: Understand. Where did you learn about these contraceptive measures and related knowledge?

Role 2: This knowledge is disseminated during the premarital checkup before marriage, and I have also seen this kind of content in books.

Role 1: Premarital examinations are popularized to medical staff, and there is also some relevant text information. Do you get some relevant information from the Internet?

Character 2: Not this.

Character 1: For example.

Role 2: I haven’t specifically heard about it, can you hear it?

Character 1: I didn't hear it just now, there was a break.

Role 2: I didn’t say anything on purpose…I looked it up on the Internet.

Role 1: I understand. The only time you tried to find out more about it was during the premarital checkup before marriage, right?

Role 2: Yes, this knowledge will be provided during premarital checkups.

Role 1: Do you sometimes talk to people around you, such as friends who have experience, about how to use contraception?

Role 2: This topic has not been discussed.

DOL_071:

Interviewer: You just mentioned that this baby is a little surprise that you got unexpectedly when you were discussing marriage. I would like to ask if you and your husband have taken any active contraceptive measures since you met?

Respondent: Yes.

Interviewer: What kind of contraceptive measures are generally taken?

Respondent: I think condoms are the safest.

Interviewer: Is this the only one? Or have you tried others?

Respondent: I haven't tried any other, only this one. I think medicine will definitely cause more harm to the body, and this is the only one I think is the safest.

Interviewer: You just mentioned some things besides birth control pills. Besides birth control pills, for example, there are other measures that you may take by yourself, such as calculating the safe period for girls or ejaculation outside the body for boys. Have you considered these?

Interviewee: I haven’t thought about this because we were not working together at the time and had very little time to see each other.

Interviewer: I understand. What is the main reason for you to take contraceptive measures?

Interviewee: Because I hadn’t considered marriage at that time, and I felt that I didn’t know enough about it.

Interviewer: I haven’t decided to get married yet, and I still don’t think I want to get pregnant.

Interviewer: Yes.

Interviewer: Have you ever been worried about, for example, health issues? For example, the spread of disease.

Respondent: Not really. We both lead a pretty healthy lifestyle, so we’ve never thought about it.

Interviewer: I see. In your daily married life, do you ask him to use condoms because you are worried about pregnancy?

Respondent: Yes.

Interviewer: Okay, do you usually take your Yuting?

Interviewee: Most girls, yes, are particularly worried. He means that you will get married sooner or later anyway.

Interviewer: Yes, sometimes there are some disagreements, but he thinks it doesn’t matter since they are getting married anyway.

Interviewee: Yes. (inaudible 00:42:44) There is no difference.

Interviewer: I see. If you have a disagreement when discussing whether to use condoms, how do you usually handle it?

Interviewee: Generally he listens to me.

Interviewer: How would you convince him?

Interviewee: Actually, I didn't need to convince him. I just said that since we were not married, if the child really came, it would be irresponsible to the child. He was quite open-minded. Once I said that, he listened and supported it.

Interviewer: If he wanted to give the reason for not wanting to use condoms in the beginning, it was mainly because he felt that he was going to get married anyway, so it would be the same whether he did it sooner or later, right?

Interviewer: Yes.

Interviewer: Yes, so what kind of contraceptive method should be used? For example, you just mentioned that you only used condoms. Who mainly decided when choosing this contraceptive method?

Interviewee: I think we both made this decision from the very beginning, and it has always been like this.

Interviewer: Did you not consider any other contraceptive measures during the whole process?

Interviewee: Yes, no.

Interviewer: I see. Where do you usually learn about contraception?

Interviewee: I feel like I learned this without a teacher. I grew up and became an adult. I seem to know it in my daily life. Finally, I didn’t say where I learned this.

Interviewer: I don’t have the impression that I took any initiative to learn anything like that.

Interviewer: Yes.

Interviewer: If you think back roughly, do you think you sometimes get some relevant information on the Internet?

Interviewee: I think so, but I can’t remember.

Interviewer: Or for example, some family members or friends who have experience, would you talk to them about how to use contraception?

Respondent: No, generally we rarely talk about such private matters.

Interviewer: I see. What about professional doctors and nurses? Do you talk to them about contraception? Do you ask them?

Respondent: We haven’t discussed this issue either.

Interviewer: It feels like I learned it naturally without any guidance.

Interviewer: Yes.

DOL_072:

Interviewer: Yes. You just mentioned that in the beginning, although you didn’t try hard or deliberately want the child, you didn’t deliberately use contraception.

Interviewee: We used contraception at that time. We used it for several years. When our child was young, we thought it was too much trouble to have and raise a child. We really didn’t want it at that time. But now the child is a little older, and we feel that the child is a little lonely. In fact, we used contraception before.

Interviewer: Before you had your first baby, did you take any contraceptive measures?

Interviewee: No.

Interviewer: After you had your first child, you felt a lot of pressure and didn’t want to have another child. Did you use contraception?

Interviewee: Yes.

Interviewer: What methods do you mainly use to prevent pregnancy?

Interviewer: I had an IUD at that time. I was wearing it.

Interviewer: Besides having an IUD inserted, have you tried any other methods?

Interviewee: No.

Interviewer: I understand. Wasn’t the main reason for having the IUD inserted also because you didn’t want to get pregnant again?

Interviewer: Yes, I really didn’t want it at that time.

Interviewer: Who proposed the IUD contraceptive method?

Interviewee: Myself.

Interviewer: You proposed it yourself. Does your husband support it?

Interviewee: He knew it too, and he could see clearly that I couldn’t take care of two people.

Interviewer: He can understand it from a practical level, but does he also support it on an emotional level?

Interviewer: I don’t think so. He was okay. He didn’t say anything against it, nor did he say anything in support of it.

Interviewer: It seems that we haven't really discussed what he thinks about this matter.

Interviewee: No.

Interviewer: I understand. You mentioned that you wanted to get an IUD, and after you told him, it seemed like you went ahead.

Interviewer: Because we actually had another child before this one, who was only 6 months old, and our current child is only 6 months old, and we got pregnant again at that time. We couldn't keep the baby at that time, so we went for an abortion directly because we were afraid of harming our bodies. They said we could just get an IUD instead, which wouldn't harm our bodies.

Interviewer: I see. So you didn’t take any proactive contraceptive measures until you got pregnant with your second child.

Interviewee: Yes.

Interviewer: I see. You just said that you have not tried any other methods except birth control, right?

Interviewee: No.

Interviewer: Where did you learn about contraception?

Interviewee: Now they are available in health centers. At that time, when I was wearing an IUD, he said it was relatively safe. If you bought a more expensive one, it wouldn't do much harm to you. I wore it for several years.

Interviewer: Mainly some local professional health centers.

Interviewer: The main reason I wore it was that I didn't have any physical reaction. I didn't react any differently to others, or feel any discomfort. I didn't feel any discomfort, so I just adapted to it.

Interviewer: I see. You first learned about contraception at the health center, and then tried it. You found it quite helpful and you didn’t feel any discomfort.

Interviewee: Yes.

Interviewer: Regarding contraceptive knowledge, have you consulted more experienced friends and family members?

Interviewee: I have asked some of them, but they couldn't give me a clear answer. They all said that everyone has their own way. Some said that just be careful, while others said that even paying attention is not enough. They said all kinds of things.

Interviewer: It feels like the information is mixed and not very helpful.

Interviewee: Yes.

Interviewee: I was in the hospital before.

Interviewer: You just talked about explaining some family planning issues in the hospital. Is it because you knew more about it in the hospital at that time?

Interviewer: At that time I was a nurse in the hospital. I worked there for more than a year, and then I quit.

DOL_073:

Role 2: We were thinking, because we had just gotten married, we just wanted to let nature take its course. If we got pregnant, we would have it. If not, forget it. We didn't take any active steps to prepare for pregnancy or anything. We just wanted to let nature take its course.

Role 1: I understand. I would also like to talk about some things regarding reproductive health. Have you and your husband taken any proactive contraceptive measures?

Role 2: Yes. We have been taking measures since the birth of the baby, because we don’t plan to have a second child.

Role 1: I am quite determined and will take measures every time. What kind of contraceptive measures do you usually take?

Role 2: Condom.

Character 1: Is there anything else besides this?

Character 2: No more.

Role 1: Is the main reason for taking contraceptive measures due to the fear of pregnancy?

Character 2: Yes.

Character 1: Have you considered, for example, preventing the spread of disease?

Character 2: No, but this.

Character 1: You say.

Role 2: I think you two are already married and have already been involved in this matter before. If you are now trying to prevent the disease, I think that's a bit too much, unless you suspect what he is doing outside.

Role 1: I understand. Who usually brings up the issue of condom use?

Role 2: He does it on his own without anyone bringing it up to him.

Role 1: No need to mention it specifically, he will use it every time.

Character 2: Yes.

Role 1: Do you two have any disagreements on the use of safety measures?

Character 2: No.

Role 1: For example, will there be disagreements regarding whether to use or not, or which contraceptive measure to take?

Character 2: No.

Role 1: Understand. Where do you usually go, or through what channels, to learn about contraception?

Role 2: I think most married women nowadays understand this.

Character 1: Did you learn this during your marriage?

Character 2: Yeah.

Role 1: Do you mean, for example, that your husband would tell you something, or how would you find out?

Role 2: No. When I said I didn’t want a second child after giving birth, because I was in the confinement period at that time, I often watched Douyin, and I once saw some men getting sterilized or something. I had just come out of the confinement period, and I felt that I was a little bit pregnant. After watching it, I said, "Why don’t you get sterilized? How can you prevent having a second child? Look, the wound is small and heals quickly." He said, "Why do we have so many condoms in our family? If you don’t want it, just wear a condom. Why do you want me to get sterilized?"

Role 1: I understand. But after seeing this method on the Internet, some contraceptive measures that men can take. You mentioned it to him, but he was not very happy about it. He thought there was an alternative method.

Character 2: He said there is this kind of thing that doesn't hurt, why do you want me to do the kind that hurts?

Character 1: I think this is a hurtful thing.

Character 2: Yes.

Role 1: Understand. In addition to the relevant knowledge that can be pushed on TikTok, have you learned it from other channels? For example, asking experienced family members or friends, or asking professional doctors and nurses, etc.?

Character 2: No.

DOL_074:

Character 1: Next, I would like to ask something about children. What was the opportunity that led you two to have a child?

Role 2: Normal pregnancy.

Character 1: Is this planned?

Role 2: I didn't have any plans, I just got married and got pregnant. I didn't use any contraception, that's all.

Role 1: I understand. Next, I would like to talk to you about some things related to reproductive health. First of all, I would like to ask, have you and your husband ever taken active contraceptive measures?

Role 2: Yes.

Role 1: What kind of contraceptive measures do you usually take?

Role 2: Mostly condoms.

Role 1: Condoms. Others include, for example, the safe period for girls, IUDs, and birth control pills.

Character 2: (inaudible 00:52:58).

Character 1: You say. Hello, can you hear me?

Role 2: It just broke. How can you consider the safe period and also consider this.

Role 1: Have you ever tried something like having boys ejaculate outside the body?

Role 2: Yes, I have.

Character 1: What about other contraceptive pills?

Role 2: My friend has taken it. She is the type who thinks that something is wrong with my period and buys me emergency contraceptive pills. I have never taken it.

Role 1: I understand. If you take safety measures, whether it is using condoms, safe periods, or ejaculation outside the vagina, is the main reason to avoid unwanted pregnancy?

Character 2: Yeah, that's right.

Role 1: Have you ever considered, for example, worrying about the spread of certain diseases?

Character 2: No.

Role 1: Understand. Who usually suggests taking contraceptive measures, such as using condoms?

Role 2: I don’t remember anything. For example, I didn’t consider having a child. I just thought it was a dangerous period, so I just wore a condom. That’s it.

Role 1: For example, if you know that it is your dangerous period, will you take the initiative to ask your husband to use it?

Character 2: Yeah, that's right.

Role 1: Do you ever have disagreements? For example, are there times when you want him to use a condom but he doesn't want to?

Character 2: No, not really.

Role 1: Yes, what kind of safety measures do you use? For example, who usually decides the three types of safety measures mentioned above, condoms, safe period and ejaculation outside the vagina?

Role 2: Me. Because I understand this cycle.

Role 1: Do you have any disagreements when it comes to which specific contraceptive method to use?

Character 2: No.

Role 1: There will be no contradiction in this matter.

Character 2: He won't get involved in this, he doesn't care.

Role 1: I understand. Generally, you have the final say. You calculate based on the cycle, and then you decide what method to use.

Character 2: Yes.

Role 1: Understand. Where do you usually learn about these contraceptive knowledge?

Role 2: On the phone.

Role 1: Is it online or on your mobile phone?

Character 2: Yes.

Role 1: Have you asked other family members or friends who have had experience, for example?

Role 2: Very little.

Role 1: Do you understand, or talk to professionals such as doctors and nurses who work in family planning?

Character 2: No.

DOL_075:

Interviewer: I understand. You just mentioned that your second baby was unexpected and unplanned. I would like to ask, have you taken any proactive contraceptive measures?

Respondent: No.

Interviewer: Did you take any contraceptive measures after giving birth to your two babies?

Respondent: No.

Interviewer: Never?

Interviewee: Yes, because after I gave birth to my first child, my menstruation came after 10 or 11 months.

Interviewer: I understand. It’s mainly due to my physical condition, so I don’t consider contraception anymore.

Interviewee: Because I didn’t think of using contraception for my first child, I got pregnant when my second child was over one year old. I thought if it was a girl I would definitely keep the baby, but when I checked it turned out to be a boy so I didn’t keep the baby.

Interviewer: I understand. Before giving birth to the second baby, she was pregnant with a boy.

Interviewee: The second one behind.

Interviewer: The second one was pregnant with a boy, I see. Otherwise, it would be three babies, three boys.

Interviewee: Yes, that would be too stressful, so I didn’t stay.

Interviewer: I understand.

Respondent: I don’t take any contraceptive measures intentionally now, but I am careful.

Interviewer: How do you usually pay attention to it? For example, do you consider the safe period, or male ejaculation outside the body?

Interviewee: Yes, in vitro.

Interviewer: Mainly outside the body, OK, I understand. In your sexual life with your husband, do you ever worry about pregnancy and want him to use the safe method of ejaculation outside the body?

Interviewee: You didn’t understand.

Interviewer: In your married life, would you ask your husband to have sex outside the vagina because you are worried about pregnancy?

Interviewee: I don't care, because his grandmother sometimes said that if he had a girl, his life would be good. The child's father felt that two boys were enough and he didn't want another girl. Although I like girls in my heart, they don't want them, and I'm afraid that if it's a boy again, I'll be the one to suffer.

Interviewer: I see.

Interviewee: So I just let things take their course. If it’s a girl, I’ll keep her. If not, I’ll not keep her.

Interviewer: I see. Have you and your husband ever had a disagreement on whether to have ejaculation outside the vagina?

Respondent: No. He had time and said he would ejaculate inside me, and I said if you want to have a child, then just ejaculate.

Interviewer: I see. Do you usually reach a consensus in the end?

Interviewee: Yes, usually outside the body.

Interviewer: I understand.

Respondent: When I have time, I will just let it go.

Interviewer: I see. Where do you usually learn about contraception?

Respondent: I didn’t try to find out, because apart from getting an IUD, there doesn’t seem to be any 100% safety measure.

Interviewer: Where did you learn about this contraceptive method?

Respondent: The old-fashioned people at home all say this, and they also see it on their phones when they have time.

Interviewer: One is that experienced older family members will tell you, and the other is that there may be some information on the Internet.

Interviewer: Yes, because some people asked if they wanted to have children, and he said no, because he already had an IUD. Some said they wanted to have an IUD because they were afraid of getting pregnant.

Interviewer: I see. In addition to talking to older family members and looking for information online, have you consulted with friends who have experience, or discussed how to use contraception?

Interviewee: No, not really.

Interviewer: What about professional doctors and nurses?

Respondent: No.

Interviewer: I see. OK. Do you think that after talking to your family about the contraceptive methods and the risks of various contraceptive methods, did it help you and your husband in choosing a contraceptive method?

Interviewee: We both have our own methods. Sometimes he goes out and sometimes I am not at home. We are rarely together. The one time we are together a week is always in vitro fertilization. I feel the chance of getting pregnant is not too high.

DOL_076:

Role 1: Next, I would like to ask some questions related to reproductive health. You just mentioned that the second baby was actually conceived relatively soon, and you got pregnant unexpectedly. I would like to know what contraceptive measures you and your husband will take during your relationship?

Role 2: This will happen.

Role 1: What kind of contraceptive measures are generally taken?

Role 2: In this case, the contraceptive measures taken are to use condoms.

Role 1: In addition to using condoms, there are other contraceptive measures, such as the safe period for girls, intrauterine devices, IUDs, or emergency contraceptive pills, short-acting contraceptive pills, and external ejaculation for boys. Have you considered these methods?

Character 2: Not this.

Role 1: Only considered condoms.

Character 2: Yes.

Character 1: OK. What is the main reason for you to adopt security measures?

Role 2: I don’t want to have any more children.

Role 1: The main reason is that I don’t want to get pregnant.

Character 2: Yes, that is to say, if you wait until you are pregnant, you should not do it anymore, and the adults will suffer too.

Role 1: Yes, have you ever worried about the spread of disease, so have you adopted safety measures?

Role 2: I haven’t worried about that.

Role 1: Understand. In your daily life, do you ask your husband to use condoms because you are worried about pregnancy?

Role 2: Yes.

Role 1: Have you ever had a disagreement on this issue? For example, you want him to use it, but he may not be willing to do so at first.

Character 2: Not this.

Role 1: Understands. Once you propose, he will immediately cooperate.

Character 2: Yes.

Role 1: I understand. Do you use condoms every time after you don’t want to get pregnant?

Role 2: Yes, I use it every time. Because I am afraid of getting pregnant. If I don't use it, it will be very harmful to my body.

Character 1: Yes. When making decisions about what kind of contraceptive method to use, do you have different opinions?

Role 2: There is basically no disagreement.

Role 1: For example, if condoms are used, both parties will be more willing to accept the use of condoms.

Character 2: Yes.

Role 1: Who usually decides whether to use condoms?

Character 2: This is my decision.

Role 1: Understand. Where do you usually learn about contraception?

Role 2: These will also be publicized in the clinic in our village.

Role 1: Some professional doctors or nurses will do some popular science propaganda.

Character 2: Yes. After all, they don’t want children, so they will go to the village to spread the word.

Role 1: I understand. Apart from this, would you go online to learn about related knowledge?

Character 2: This will happen too.

Role 1: For example, can you talk to some experienced elders at home, or experienced friends about how to use contraception?

Role 2: Usually on the phone.

Role 1: Do you know, or do you do more research on the Internet?

Character 2: Yes.

Role 1: I understand. Do you think your knowledge of contraception is more helpful from the Internet or from medical staff?

Role 2: Network, online publicity.

Role 1: Understand. It would be more helpful to search for relevant knowledge on the Internet.

Role 2: Yes, there is a relatively complete description on the Internet.

DOL_077:

Role 1: I understand. Next, there are some questions related to reproductive health. Have you and your husband ever taken any contraceptive measures?

Character 2: Yes.

Role 1: What kind of contraceptive measures do you usually take? For example, we usually talk about condoms and women's safe period.

Character 2: Yes, condoms.

Character 1: Mainly condoms, right?

Character 2: Yes.

Role 1: Have you ever chosen other methods such as safe period, intrauterine device, emergency contraceptive pills, short-acting contraceptive pills, and male ejaculation outside the body?

Character 2: No, basically just use condoms.

Role 1: I understand. How often do you use condoms? Do you use them every time or occasionally?

Role 2: Basically used every time.

Role 1: Understand. What are the main reasons for using condoms?

Role 2: We don’t want a second child either, it’s better to be safe.

Role 1: Understand. After giving birth, I will still think about and worry about unexpected pregnancy.

Character 2: Yes.

Role 1: Understand. In sexual intercourse between couples, who usually suggests using condoms?

Character 2: We both think about it anyway.

Character 1: No one brings this up more often.

Character 2: It should be me.

Role 1: You will bring it up more often. Have you ever had a situation where, for example, you want your husband to use condoms, but he is not willing to do so, or is not willing to do so at the beginning?

Character 2: No.

Role 1: Understand.

Character 2: Yes.

Role 1: Has the reverse situation ever occurred? For example, your husband wants to use it, but you are not willing?

Character 2: No.

Role 1: Understand.

Character 2: Yes.

Role 1: Regarding the choice of contraceptive method, for example, you just mentioned that you usually use condoms. Who decides? Because among the many other contraceptive methods, who decides to choose condoms?

Role 2: I should be the one who decides, because I feel like, what was it called last month? IUD, no, what was it?

Character 1: Yes, the IUD.

Character 2: Yes, but I feel like that’s not very comfortable.

Role 1: I don’t really think about that.

Character 2: Yes.

Character 1: What about other methods?

Role 2: I haven't considered other methods. I feel like condoms are the simplest and seem pretty safe.

Role 1: Simple and safe.

Role 2: Yes, I’m afraid of side effects from taking that medicine, so I dare not use it. It’s okay to use it occasionally, but I don’t feel it’s safe if I use it all the time.

Role 1: Understood. Have you and your husband ever taken birth control pills in your daily life?

Character 2: I only remember three or two times, very rarely.

Role 1: Understand. Under what circumstances do people usually decide to use contraceptive pills?

Role 2: Sometimes there are no condoms at home.

Role 1: Understand.

Character 2: Yes.

Role 1: Have you ever had any disagreements about using different methods of contraception?

Role 2: There is no difference. I always use this one, as if I have determined it. I have never thought about this issue.

Role 1: Understand. You think that IUDs or birth control pills may be harmful to the body. If you express your unwillingness to use this method, what is your husband's attitude?

Character 2: He had no objection.

Role 1: Or do you support your ideas and choices?

Character 2: Yes.

Role 1: Where do you usually learn about these contraceptive knowledge?

Role 2: Sometimes our hospital will send out some promotional materials, and our gynecologists will send you some links on WeChat.

Role 1: Understand that some professional medical staff will provide some education and the like.

Character 2: Yes.

Role 1: Do you think this is helpful?

Role 2: I think it's OK, because we never seem to check these things ourselves, and basically rely on them. If they promote it, we will pay attention to it, but if they don't promote it, we won't check these things on purpose.

Role 1: Understand. If you don’t actively learn about it, it is still helpful if a professional can introduce it.

Character 2: Yes.

Role 1: I understand. You just said that you probably wouldn't actively look up related information online. In your daily life, have you ever asked experienced friends or family members about how to prevent pregnancy?

Role 2: No, basically if you want to ask, you can go to the hospital and talk to a gynecologist or something.

DOL_078:

Role 1: I understand. I would like to ask if you and your husband have ever taken any contraceptive measures?

Role 2: Taken.

Role 1: What kind of contraceptive measures do you usually take?

Role 2: I got the IUD from the hospital.

Character 1: When was it before?

Role 2: After giving birth to the first child.

Role 1: I understand.

Role 2: Yes, I wore the IUD after giving birth to my first child, and took it off when I was preparing for my second child. Now I use condoms.

Role 1: I understand.

Role 2: Not wearing an IUD yet.

Role 1: Are you considering getting another IUD in the future?

Role 2: I have this plan. I feel it will be painful to remove the IUD, so I have been hesitating.

Role 1: I see. What is the main reason why you take contraceptive measures?

Role 2: I’m afraid it will happen again.

Role 1: Yes. Have you ever considered worrying about the spread of disease?

Role 2: I haven't thought about it. Because there shouldn't be any diseases between husband and wife. I'm just worried about getting pregnant again unexpectedly, because I don't plan to have another one.

Role 1: Have you ever experienced this, for example, occasionally using emergency contraceptives or short-acting contraceptives, or using the safe period or having ejaculation outside the body?

Role 2: I have taken birth control pills, but people say they are harmful to the body. So I won't take them anymore.

Role 1: Understand. I have never tried things like calculating the safe period and ejaculating outside the vagina.

Role 2: I haven't calculated it. I'm afraid I won't be able to calculate it accurately, what if I make a mistake.

Role 1: Yes, I understand. In daily sex, do you actively ask your husband to use condoms because you are worried about pregnancy?

Character 2: Yes.

Role 1: Are there situations where you want him to use it, but he may not be willing to do so at first?

Role 2: No, he is quite cooperative. He is just afraid of what if she gets pregnant? He doesn't plan to keep it. If he does it again, it will hurt me, right? My husband is quite cooperative.

Role 1: Usually you are the one who brings it up, and he will usually cooperate after you do so.

Role 2: Yes. As long as you do that, he will.

Role 1: I understand. At present, I have also had an IUD inserted after giving birth to my first child. Sometimes I have taken emergency contraceptive pills once or twice. Who usually decides which contraceptive method to use, such as condoms, IUDs, and contraceptive pills?

Role 2: I usually decide to take the pill. Condoms are normal. If you are afraid of something, you should prepare in advance. Now my husband wants me to wear an IUD again for safety, but I don't want to wear it now because I am afraid.

Role 1: I see. How do you discuss the issue of having an IUD with your husband now?

Role 2: He suggested that I go to a higher school, which is safer. I kept saying I would think about it, and since I didn't know what it was, I decided to do it this way.

Character 1: I am still a little worried, because after all, it was very uncomfortable when I had my IUD removed last time, so I don’t really want to do it.

Role 2: Yes. Some of them seem to be unwilling to accept it. Some of my friends around me also seem to be not very (inaudible 00:50:35). They have back pain or some other problems. Anyway, they have always been a little resistant. After giving birth to my second child, I have not taken care of it.

Role 1: Understand. I feel that one reason is that my previous experience was not very good, and the other is that I have also seen some side effects among my friends.

Character 2: Yes. So I am still hesitating.

Role 1: Generally speaking, you mentioned that you might ask your friends about contraception, or you might talk to them about it, and there might be some side effects. In addition to talking to your friends, have you talked to other people, such as professional doctors and nurses, about contraception-related knowledge?

Role 2: No. When I went to have the IUD removed or put it in, I told the doctor which one was better, and I didn't try (inaudible 00:51:42). I didn't try anything else, but there are side effects, and everyone's body is different, so it doesn't mean that everyone will experience that kind of situation.

Role 1: When getting an IUD inserted, you will learn about the related side effects, right?

Role 2: Yes, I didn’t go to the hospital specifically to find out.

Role 1: I understand. How did you learn about each contraceptive method?

Role 2: How did you get to know it? I got to know it quite naturally. I didn’t try to find out specifically.

Character 1: How did you know?

Role 2: Watching it on mobile phone.

Role 1: Saw some information on the Internet.

Character 2: Yes.

Role 1: I understand. For example, have you consulted some experienced elders at home?

Character 2: No. I think I know all of this after I get married and let it happen naturally.

Role 1: Understand, let it be, and feel like you will know it during the marriage process.

Role 2: I just knew the feeling, but I never asked my family about it.

DOL_079:

Role 1: Understand. You just said that the baby came when you were not ready at first. Have you and your wife taken any contraceptive measures?

Character 2: Yes, it has always existed.

Role 1: What kind of contraceptive measures will be mainly taken?

Role 2: Condom.

Character 1: Anything else?

Role 2: None else.

Character 1: Have you ever taken birth control pills or anything like that?

Character 2: No, I really don’t have that.

Role 1: Do girls usually calculate the safe period?

Role 2: I may not have paid much attention to this aspect in normal times.

Role 1: Understand. Have boys ever considered methods like ejaculation outside the vagina?

Character 2: No.

Character 1: What about things like IUDs?

Character 2: I've never thought about that.

Role 1: I understand. What is the main reason for you to take this measure of condoms?

Role 2: More convenient and safer.

Role 1: I really don't want to get pregnant, so I consider this to be safer.

Character 2: Yes.

Role 1: Have you considered the spread of sexually transmitted diseases? To prevent the spread of sexually transmitted diseases.

Character 2: I probably haven’t thought about this aspect. I haven’t thought about this aspect.

Role 1: In your daily sexual life, does your wife ask you to use condoms because she is worried about pregnancy?

Role 2: Put it on on your own initiative during the period.

Role 1: You will wear it on your own initiative without her asking.

Character 2: Yes.

Role 1: I see. Have you ever had some small disagreements on this matter? For example, one person wants to use it, but the other doesn't?

Character 2: No, she definitely wants me to use it, but I’ve never thought about not using it, I use it every time.

Role 1: You will use it whenever she asks you to.

Character 2: Yes.

Role 1: Even when it’s not mentioned, you will sometimes take the initiative to use it.

Character 2: Yes.

Role 1: Understand. When choosing a specific contraceptive method, such as condoms, who is the main person who makes the decision?

Role 2: This should be the general trend. Anyway, I still make the decision in this regard.

Role 1: On the one hand, you feel that this is a major trend, and on the other hand, you also propose to use this method.

Role 2: Yes, the safety rate is relatively high.

Role 1: The main consideration was the safety rate. Were there any disagreements? For example, was there any possibility that she would like to use other contraceptive methods?

Role 2: If I give her medicine, it might have a serious impact and not be good for her health, so I never thought about it.

Role 1: Understand. Where do you usually learn about contraception?

Role 2: Usually online.

Role 1: Check it online.

Role 2: Yes, surf the Internet.

Role 1: Have you tried to ask family members or friends who have experience about contraception?

Character 2: No, that was never discussed.

Role 1: Understand. Would professional doctors and nurses talk about this?

Role 2: I don’t know that either. I usually don’t go to the hospital unless there is something wrong. Young people nowadays may learn more about it online.

Role 1: It’s more convenient online, indeed.

Character 2: Yes.

DOL_080:

Role 1: Understand. How has your husband been using contraception?

Role 2: I have an IUD now. I had it installed after I gave birth to my daughter.

Character 1: That's mainly because I don't want to get pregnant again.

Role 2: Yes, I don't want to suffer anymore. When I gave birth to my daughter, no one asked me. If they had asked me, I would have had a sterilization. I thought at the time, if we can live together forever, then we can live together. If not, I said that if we separate, we will definitely have a son and a daughter. I said I don't want to have another child, not with anyone. I had a caesarean section at the time, and they didn't ask me. I was quite nervous at the time and forgot about it. Before giving birth, I was thinking that I would have a sterilization. No matter who I marry or what I do in the future, I don't want to have children anymore. Later, nothing happened. Half a year later, I asked someone. There was an aunt in our family who was in the hospital. I said that I had an IUD inserted, and he said that was OK. He said it was OK, and now I have an IUD inserted.

Role 1: I see. In addition to the above, have you considered any other security measures?

Role 2: Not really. I've had the IUD for a few years now, and I've been working for three years now, and I haven't thought about any other safety tests.

Role 1: Do you know if other approaches have been taken before?

Role 2: I used to wear that kind of condom.

Role 1: Understand. Have you tried using birth control pills or ejaculation outside the body? There is also a safe period.

Role 2: I have ejaculated outside the body a few times, but I never take birth control pills. I think the drug is toxic and is definitely not good for a woman's body.

Role 1: I see, yes. Before the IUD was inserted, would you normally ask him to use condoms because you were worried about pregnancy?

Role 2: He is very self-aware, you know. He is very self-aware. Anyway, he doesn't want children.

Role 1: I understand. This is mainly due to economic pressure.

Character 2: Yes, because we were all young at that time and felt it was too early for you to have children and there was no time for you to enjoy your own world.

Role 1: I understand. Have you ever had a situation where one person wants to use condoms and the other doesn't?

Character 2: Not really.

Role 1: Still relatively consistent.

Role 2: We were all pretty much unanimous. The child was still young at the time, and we didn’t want any accidents to happen, so there was no controversy over the matter.

Role 1: Understand. When you and your husband choose a specific contraceptive method, for example, we just mentioned condoms, and then there is the IUD, including the occasional ejaculation outside the vagina before, who usually decides which method to choose?

Role 2: It's usually me. I was the one who got the IUD. I got it because I thought it was more risky for boys to get sterilized. They would say that men wouldn't be able to get an erection or something. Later, I thought that although it wasn't very safe for women to get the IUD either, most girls would get it anyway, so I followed the crowd and got it too.

Role 1: I understand. For example, do you have different opinions on the different methods you choose, such as condoms, and whether to choose condoms or ejaculation outside the vagina? For example, does anyone prefer one over the other?

Role 2: I haven't told him specifically, it depends on him. If he doesn't want to wear it this time, he may choose to ejaculate outside the body. If he wants to wear it, he can wear it. I can do whatever I want. It's his own decision.

Role 1: I understand. That is, as long as he chooses to take contraceptive measures, you generally have no disagreement with him?

Character 2: Yes.

Role 1: Where do you usually learn about contraception?

Role 2: Generally, everyone knows it. It seems that there is no specific information learned from somewhere. It seems that it is common sense and one knows it naturally.

Role 1: I see. Would you search online to find out what the different methods are?

Role 2: I haven't searched it before. For example, if you are using an external condom, as long as you don't ejaculate inside, or if you ejaculate inside the condom, you will definitely not get pregnant, so I haven't searched it before.

Role 1: I understand. Have you ever consulted or discussed with more experienced family members or friends about how to prevent pregnancy?

Character 2: No, people in rural areas are more reserved. We have never discussed this kind of thing.

Role 1: Understand. Have you ever talked to professional doctors and nurses about how to use contraception?

Role 2: I haven't talked about it. After I had the IUD inserted, the doctor said it was a way to protect myself, so we never talked about sensitive words.

DOL_081:

Interviewer: Have you and your husband ever taken any proactive contraceptive measures?

Respondent: Yes.

Interviewer: What kind of contraceptive measures are generally taken?

Respondent: Wear a condom.

Interviewer: Anything else?

Interviewee: Never, never took any medicine or anything like that.

Interviewer: I understand. For example, have you ever considered the calculation of the safe period or ejaculation outside the vagina?

Respondent: No, I never thought about it.

Interviewer: I see. What is the main reason for you to take contraceptive measures?

Interviewer: I just said that I don’t want to have children when my career is on the rise.

Interviewer: I see. Have you considered preventing the spread of sexually transmitted diseases?

Interviewee: I haven't really thought about this, because we think that sexually transmitted diseases are usually transmitted between boys or girls. He may have a messy private life outside. In this case, I think generally, unless there is too much going on between husband and wife, it shouldn't happen.

Interviewer: I understand. In your daily life, do you actively ask your husband to use condoms because you are worried about pregnancy?

Respondent: Yes.

Interviewer: Have there ever been disagreements on this matter? For example, one party wants to use a condom, but the other party is reluctant, or reluctant at first.

Interviewee: No, he respects me a lot.

Interviewer: I want to know, when you are choosing contraceptive measures, for example, when you decide to use condoms as a contraceptive measure, who makes the decision?

Interviewee: We both think so. We think this is the safest and harmless method.

Interviewer: Yes. Regarding what you just mentioned, such as how to prevent pregnancy and what kind of contraceptive measures are the safest, etc., where did you learn this knowledge about contraception?

Interviewee: In books, movies and TV shows, all of these.

Interviewer: Do you search for relevant information on the Internet?

Respondent: I haven’t searched it. I think adults should understand this.

Interviewer: For example, when you say books, do you generally mean some professional popular science books, or also some literary and artistic works?

Interviewee: There are no popular science books. I think when you read a literary work and it talks about these things, you might take a closer look. That's it.

Interviewer: I understand. There are related scenes in movies and TV shows, so you can learn a little bit from that.

Interviewer: Yes.

Interviewer: I see. For example, do you consult with family members who have experience or more experienced friends about how to use contraception?

Interviewee: No, I never talk about this with my family.

Interviewer: Very good. Did you ask for advice or professional knowledge from professional medical staff?

Respondent: No, I haven’t.

DOL_082:

Role 1: I understand. Can I ask how you and your husband have been using contraception?

Role 2: Menstrual period, according to the menstrual period.

Role 1: Yes, I just heard you mention that you might calculate the safe period, right?

Character 2: Yes.

Role 1: Apart from this, have you taken any other contraceptive measures?

Role 2: Wear a condom.

Character 1: Anything else?

Character 2: No more.

Role 1: Understand. Have you ever taken emergency contraceptive pills or short-acting contraceptive pills, or have boys ever taken any methods like ejaculation outside the body?

Character 2: No.

Role 1: Understand. What is the main reason for you to take contraceptive measures?

Role 2: The main reason is that I don’t want to have children.

Role 1: Yes. Are you worried about the spread of sexually transmitted diseases?

Character 2: I haven’t thought about that.

Role 1: Understand. During your usual sexual life, would you actively ask your husband to use condoms because you are worried about pregnancy?

Character 2: No.

Role 1: Have there been any small disagreements on this matter? For example, one person may want to use it, while the other may not be willing to do so at first.

Role 2: Man, my husband doesn't really like wearing condoms.

Role 1: I understand. How would you discuss this situation? How would you solve it?

Role 2: I will tell him why we cannot have children now, and he will understand.

Role 1: If you tell him the reason why you can’t have children, he will still understand and will still use it.

Character 2: Yes.

Role 1: Understand. Does it happen every time?

Character 2: No, occasionally.

Role 1: This disagreement still occurs occasionally, so I don't use it.

Character 2: Yes.

Role 1: Understand.

Role 2: For example, if his menstrual period is calculated, he doesn't want to wear a condom.

Role 1: Understands that if it is during the safe period, he will not wear it.

Character 2: Yes.

Role 1: When it comes to choosing which contraceptive method to use, such as condoms or the safe period, who usually makes the main decision?

Role 2: Me.

Role 1: How would you propose it?

Role 2: I would say, for example, today is the safe period, please put on a condom, and I would remind him.

Role 1: Understand. For example, when you decide whether to calculate the safe period or use condoms, has your husband ever had a different opinion from you?

Role 2: Yes, he thinks every day is a safe period.

Role 1: Understand.

Character 2: Doesn't like wearing condoms.

Role 1: I see. What would you say to him in this situation?

Role 2: I would tell him that the first seven days and the last seven days of menstruation are the safe period, and it is not safe after that period.

Character 1: Does he generally understand?

Role 2: Yes, I can still understand it. If it was like this time when we were having a baby, and we were pregnant before, there would be no other way, we couldn’t go to work, and we had to have this child.

Role 1: Yes, I will still think about the consequences. Considering that you two don’t want to have children recently, I will still agree.

Character 2: Yes.

Role 1: Understand. Where did you learn these contraceptive-related knowledge?

Role 2: We learned it when we were in school. When we were in school, we started to have physiology classes when we were in junior high school, and we all understood it.

Role 1: I have learned this in physiology class.

Character 2: Yes.

Role 2: Yes.

Role 1: For example, would you go online to learn some relevant knowledge?

Role 2: I will also try to understand.

Role 1: What do you usually search for?

Role 2: I will search for some contraceptive methods.

Role 1: Regarding specific contraceptive methods, do you usually ask family members or friends who have experience about how to use contraception?

Role 2: I haven’t asked because there is so much information on the Internet now.

Role 1: Understand. One reason is that you may have learned some of this in physiology class in junior high school, and the other is that it is very convenient to learn this knowledge online.

Character 2: Yes.

Role 1: Have you ever talked to professionals such as doctors or nurses about how to use contraception?

Role 2: No, I haven't talked about it. I have talked about it. I have an IUD.

Role 1: Yes, you found out about the IUD insertion from the doctors and nurses, right?

Character 2: Yes.

DOL_083:

Interviewer: Have you and your husband always taken the initiative to take contraceptive measures?

Respondent: Yes.

Interviewer: What kind of contraceptive methods do you usually use?

Respondent: Condoms.

Interviewer: Besides condoms, have you considered any other methods?

Respondent: The others are useless.

Interviewer: Have you ever tried some of the commonly used methods here, including calculating the safe period for women, intrauterine devices, emergency or short-acting contraceptive pills, and male external ejaculation?

Respondent: No, I don't think there is an absolute safe period. I think no matter when, there is still a certain risk. Contraceptive pills are not good for the body, there is nothing else.

Interviewer: Actually, I looked into some other measures and understood their impacts before making a decision.

Respondent: I haven’t used any other ones.

Interviewer: I understand. What is the main reason for you to take contraceptive measures?

Interviewee: I don't plan to have children now, but I must take some measures. Otherwise, even if I want to have a second child, I must have a plan. For example, you need to take folic acid in advance, adjust your body, and so on. You need to do something first in terms of the right time, right place, and right people.

Interviewer: I understand.

Interviewer: It’s not like there were some unexpected things, like I caught a cold today, and then I got pregnant, and I took medicine, and so on, it’s just not good.

Interviewer: The main thing is to ensure that the baby is healthy and your body is healthy during pregnancy.

Interviewee: Yes, this is definitely something we need to consider.

Interviewer: When taking safety measures, have you ever considered the risk of spreading sexually transmitted diseases?

Interviewee: I haven’t thought about this.

Interviewer: I understand. During your sexual intercourse, do you actively ask your husband to use condoms because you are worried about pregnancy? Yes.

Interviewee: Have there been any minor disagreements in this matter? For example, one person wanted to use another person but was reluctant at first?

Interviewer: Yes, sometimes he doesn’t want to use it. Anyway, he doesn’t want to use it. But in the end, if you don’t use it, it’s not a problem, so he won’t say anything.

Respondent: Will you tell him what the consequences will be if he doesn't get pregnant?

Interviewer: Yes, he also knows the consequences.

Respondent: It just needs to be emphasized again.

Interviewer: Yes.

Interviewee: I understand. Who was the main person who made the decision when choosing this method of contraception?

Interviewer: We have always used condoms, and no one has made the decision to use other contraceptives. We think this is safer. As you said, it may play a role in the spread of diseases.

Interviewee: We didn’t consider anything else at the beginning, and both parties unanimously chose this one.

Interviewer: Yes.

Respondent: Yes. Where do you usually learn about contraception?

Interviewer: I know it to some extent. I can’t say I understand it very thoroughly, but I know the basics.

Respondent: For example, would you search for information about contraception on the Internet?

Interviewer: I didn’t search online on purpose, but here we do it before we get married. It seems like there was some kind of examination or something before we get the marriage certificate. It seems like it was mentioned. It seems like it happened.

Respondent: Maybe I talked to professional doctors and nurses during the marriage checkup.

Interviewer: Yes, they should have popularized this kind of thing. I remember that they did have it. I remember that they did have it.

Interviewee: I understand. The hospital may provide some relevant popular science work during the physical examination.

Interviewer: Yes.

Interviewer: After hearing these popular science articles in the hospital, did they help you with contraception in your later married life?

Interviewer: Yes, it is helpful. It certainly doesn’t hurt to know more.

Respondent: Yes. In addition, I have also consulted with experienced family members or friends to learn or discuss how to use contraception.

Interviewer: No, after all, these are relatively private topics and I haven’t discussed them with anyone.

DOL_084:

Interviewer: Have you and your husband ever taken any proactive contraceptive measures?

Respondent: Yes.

Interviewer: What approach do you usually take?

Interviewee: I went to the hospital and had the IUD inserted.

Interviewer: After giving birth to the baby?

Interviewer: Yes.

Interviewer: Did you take any contraceptive measures before having your baby?

Interviewee: No, not that one.

Interviewer: How did you decide to wear an IUD? Was it you who proposed it, or your husband?

Interviewer: After I gave birth to the child, my mother said that I should just have one child and then I would go after I recovered.

Interviewer: I see. So I didn’t want to have any more children, so I got an IUD.

Interviewer: Yes.

Interviewer: After having the IUD inserted, have you taken any other contraceptive measures for any other reasons?

Interviewee: Not really.

Interviewer: I understand. Have you ever asked your husband about using condoms because you were worried about getting pregnant? Before coming to Shanghai, did you hope that your husband would use condoms because you were worried about getting pregnant?

Respondent: I was not allowed to have sex right after giving birth, and I was not allowed to have sex until I recovered.

Interviewer: So as soon as your body recovered, you got an IUD installed.

Interviewer: Yes.

Interviewer: I see. Have you ever had any disagreement with your husband about the IUD?

Respondent: No.

Interviewer: He is also very supportive.

Interviewee: Yes, I am quite supportive.

Interviewer: I see. Where do you usually learn about contraception?

Interviewee: That’s what my mother said.

Interviewer: Mom will tell you on her own initiative.

Respondent: Yes. My mother said to put it on and not wear it anymore, and I said okay.

Interviewer: When your mother introduced you to the idea of having an IUD inserted, she gave you a brief introduction to the method.

Interviewee: Not really, I just went to the hospital.

Interviewer: When she went to the hospital, she said something, such as paying attention to safety and so on. These things were introduced.

Interviewee: That wasn’t mentioned.

Interviewer: I understand. When you went to the hospital later, did you ask the doctors and nurses for more detailed information about the contraceptive method of IUD?

Interviewee: No, the doctor wouldn't explain so much to you. When I gave birth, there were a lot of people in the hospital, such as the obstetrics and gynecology department. For example, if you wanted to have an IUD inserted, you just lined up and got it done.

Interviewer: I understand. I don’t have time to educate everyone about science.

Interviewer: Yes.

Interviewer: In terms of contraception, besides your mother recommending this method, do you usually talk to friends with experience about related matters? About how to use contraception?

Respondent: Yes, they do. They don’t wear IUDs because they are afraid of hurting their bodies or causing inflammation, so they all use condoms.

Interviewer: I am still worried that having an IUD may affect my health.

Interviewer: Yes.

Interviewer: Did talking to your friends and your mother help you make your final decision?

Respondent: It’s okay. I think it’s more convenient to wear an IUD.

Interviewer: I understand. After comprehensive consideration, I decided to get an IUD.

Interviewer: Yes.

Interviewer: Also about contraception knowledge, for example, finding out relevant information on the Internet yourself?

Interviewee: I didn’t search it. The Internet didn’t seem to be that developed at that time.

Interviewer: I see. It’s not that convenient.

Interviewee: Yes, it’s not that convenient.

DOL_085:

Interviewer: I understand. OK, next I would like to ask some questions related to reproductive health. Have you and your husband ever taken any contraceptive measures?

Respondent: Yes.

Interviewer: What method do you usually choose?

Interviewee: Sheung Wan.

Interviewer: Did you have the IUD inserted after you gave birth to your two babies?

Interviewee: Yes, after giving birth to my second child.

Interviewer: Who proposed the decision to have an IUD inserted?

Interviewee: I proposed it.

Interviewer: What were your main considerations at the time?

Respondent: Because I am easy to conceive, the second child would be an unexpected pregnancy.

Interviewer: Yes. When you proposed to have an IUD, what did your husband think?

Interviewee: He is more supportive and agrees.

Interviewer: I understand. Before getting the IUD or before having your second child, did you take any contraceptive measures?

Respondent: No, after I gave birth to my second child, it took me more than a year, almost two years, before I got my period.

Interviewer: I understand. What about when you first got married?

Respondent: No, when I first got married, because I wanted a baby, but then I didn’t.

Interviewer: I understand that, in your entire married life, in addition to having an IUD after giving birth to your two children, have you taken any other contraceptive measures? For example, considering the safe period or ejaculation outside the vagina, as well as birth control pills, etc.?

Respondent: Yes, I took into consideration ejaculation outside the vagina, birth control pills, and the safe period.

Respondent: Yes, we considered withdrawal, birth control pills, and safe periods.

Interviewer: Condoms were never used.

Interviewer: Yes.

Interviewer: I understand. For example, when choosing a specific contraceptive method, who usually makes the decision? In addition to the IUD you just mentioned, which was proposed by you, what about the others?

Interviewee: It seems that I decide everything else.

Interviewer: You make all the decisions. Usually, you will bring it up, and then will your husband disagree with you?

Respondent: No.

Interviewer: When it comes to wanting to take contraceptive measures, is it usually you who brings it up? Or is it both of you?

Respondent: Both of them.

Interviewer: Have you ever asked your husband about this because you were worried about getting pregnant, for example, asking him to use a condom or have external ejaculation?

Interviewer: Yes.

Interviewer: Have you ever had different opinions on this matter? For example, you want him to use a condom, but he doesn't want to or doesn't want to at first. Yes, or you want to.

Respondent: No.

Interviewer: You don’t have that, do you?

Respondent: No.

Interviewer: Have there been any disagreements on the contraceptive measure of ejaculation outside the body?

Respondent: No.

Interviewer: What about birth control pills?

Respondent: Neither.

Interviewer: Yes. Where do you usually learn about contraception?

Interviewee: Baidu. I usually look at Baidu more.

Interviewer: Go search and see what methods are available, and then choose which one.

Interviewer: Yes.

Interviewer: Have you consulted family members or friends who have had experience with this issue?

Respondent: No.

Interviewer: Have you consulted any professional doctors or nurses?

Respondent: Yes.

Interviewer: Which do you think is more helpful, this or getting information on the Internet? Or do you think both are helpful?

Respondent: I think both are helpful.

DOL_086:

Interviewer: I understand. OK, next I would like to ask something about reproductive health. Have you and your wife ever taken any contraceptive measures?

Interviewee: Actively, but only occasionally.

Interviewer: Occasionally.

Interviewee: Occasionally, yes.

Interviewer: What kind of contraceptive measures are generally taken?

Interviewee: That means wearing a condom.

Interviewer: Have you tried anything else besides this?

Interviewee: Other ejaculation outside the body, occasionally.

Interviewer: Anything else? For example, has your wife ever taken birth control pills?

Interviewer: No, I wouldn’t let her eat that kind of food. After all, it’s too harmful to the body. I feel it’s too harmful.

Interviewer: I understand. When you took contraceptive measures, was it mainly because you were worried about getting pregnant?

Interviewee: Yes. After all, they are two boys.

Interviewer: Yes, were there any other considerations?

Interviewee: I haven't done anything else on purpose, except these two methods.

Interviewer: I understand. Has your wife ever asked you to use condoms because she was worried about pregnancy?

Interviewee: I am usually more proactive. I am mainly afraid of having another baby. If it is a girl, it is okay. If it is a boy, there is a high probability that I will not want it. If I do not want it, then my wife will have to suffer, right?

Interviewer: I understand. So, have you ever had a disagreement on whether to use condoms? For example, one person wants to use it, while the other doesn't.

Interviewee: I don’t really have that.

Interviewer: I understand. How do you usually discuss which specific contraceptive method to adopt? For example, as you just mentioned, either condoms or withdrawal, how do you usually discuss it?

Interviewee: How to discuss this? I don’t think it’s necessary. If you have something ready, you will use it naturally. If you don’t have it or don’t have it, you will adopt another strategy.

Interviewer: I understand. Did you have any disagreements on which specific approach to take?

Interviewee: No, I really don't. After all, I don't want to take the risk of having another child. A girl is better. If it's a boy, I can say I can't afford it. I can't afford it if it's a boy.

Interviewer: Yes. Where do you usually get information about contraception?

Interviewee: Do I need to know about this? I feel like this is very normal. It's not like before marriage, when people couldn't support their children, right? Now it seems that in rural areas, people are more open to supporting their children, and it feels normal. Before marriage, young people would feel shy when discussing this topic in rural areas. But now, even the children in junior high school know about it.

Interviewer: If you recall, how did you first learn about these things?

Interviewer: Was it you who first proposed to take this measure or something else?

Interviewer: Yes, you took contraceptive measures. How did you first get the relevant knowledge about contraception?

Interviewer: At first I didn’t watch that kind of movies, you know?

Interviewer: Was that on the Internet? Or?

Interviewee: Online.

Interviewer: So you knew more at the beginning.

Interviewee: At first, I was curious and went to Internet cafes. At that time, Internet cafes were not that kind of thing. Our rural area was still relatively backward. There were few Internet cafes, and I had little exposure to such things. I only watched others playing in Internet cafes and stood behind them to watch.

Interviewer: Yes. And then in the process of watching, the initial understanding is learning in the process of watching.

Interviewee: Yes.

Interviewer: Have you ever asked family members or friends who have experience about how to use contraception?

Interviewee: I haven’t discussed this with my family, but I have discussed it with my friends.

Interviewer: Besides talking to your friends, have you ever talked to professional doctors or nurses about how to use contraception?

Interviewee: Not yet.

Interviewer: I see. Do you think talking with friends or learning about it online can help you understand contraceptive knowledge?

Interviewee: Yes. After all, you learned it from the Internet in the beginning.

Interviewer: Do you understand the help in talking with friends?

Interviewee: The topics that men talk about are always the same. Men talk about women, you know? They always talk about these. Isn't that just chatting about the mountains? The topics that men talk about are (inaudible 01:03:58).

Interviewer: Do you think that when you talk to your friends about different contraceptive measures, how to choose them, etc., will it be helpful in practical terms?

Interviewee: I really don’t have one. After all, there are only these two methods.

DOL_087:

Interviewer: That's right. Have you taken any contraceptive measures since you got married to your husband?

Interviewee: I am afraid of using it. What is that called? I am afraid of having an IUD. My sister said she would bleed when she had an IUD. Later, she used birth control. I was afraid, so I asked my husband to use a condom. I didn’t have anything.

Interviewer: I understand. In addition to condoms, have you tried other methods, such as calculating the safe period or taking birth control pills, and ejaculation outside the body? Have you tried these?

Respondent: No. Why? I don’t know how to calculate that. Then I use condoms for everything. Most people use condoms, nothing else.

Interviewer: When you wanted to avoid pregnancy, did you use condoms every time?

Interviewee: No, not even sometimes.

Interviewer: I understand.

Interviewee: If I don't wear it, then there's nothing I can do. If I'm pregnant, then I'll have it. Usually before I come here, isn't it a safe period? Anyway, sometimes it's just like that. It's OK, I'm lucky. One time I forgot to take the contraceptive pill.

Interviewer: I understand.

Respondent: If you are afraid of getting pregnant, you can take birth control pills. Buy them now.

Interviewer: I understand. Generally speaking, if you use condoms, do you take the initiative to ask your husband to use condoms because you are worried about pregnancy?

Interviewee: I would also mention it, and he would be quite conscious of it. He is also worried that I would get pregnant. If I got pregnant, I would suffer, and he would also suffer because of his money.

Interviewer: That is.

Interviewer: Indeed.

Interviewer: He was worried about the children.

Interviewer: You say.

Interviewee: I said that if I get pregnant and have the baby, there will be no one to take care of my son.

Interviewer: Indeed.

Interviewee: He is worried too.

Interviewer: Who usually makes the final decision on whether to use condoms or take birth control pills later?

Respondent: He buys them himself. He usually buys them himself. He buys a lot of them. He uses them. He is used to it. Most of the time, he is the one who negotiates. He will take the initiative to use them. He usually uses them.

Interviewer: I understand. Have you ever wanted him to use it but he didn’t want to?

Respondent: This rarely happens.

Interviewer: Sometimes, if it appears, because you just said that there may be some, it is not used every time. If in this case, how do you usually discuss it?

Interviewee: There is nothing to discuss. If he doesn’t want it, then he can have a child.

Interviewer: If he really doesn’t want to, you won’t force him?

Respondent: I won’t force it. If it doesn’t work, just take some birth control pills.

Interviewer: I understand. For example, what does your husband think about taking birth control pills?

Interviewee: I don’t eat it often, and I haven’t talked about it. Anyway, you talk, and when the time comes, I eat, and it has nothing to do with him.

Interviewer: You yourself clearly don't want it, so you won't tell him again and you will just take birth control pills yourself.

Interviewee: That’s something my family has prepared.

Interviewer: I understand.

Interviewer: He also knew that if he didn’t use a condom, I would take the medicine.

Interviewer: I see. Where did you learn about contraception? For example, condoms, birth control pills, safe periods, and IUDs?

Interviewee: I don’t know much, little sister, everyone knows this. Although I don’t have a high level of education, I think I know this, right? My little sister and my elder sister all talk about this among friends, and sometimes they talk about it when they chat.

Interviewer: Experienced family members sometimes chat with each other and ask each other questions.

Interviewee: Yes, it seems that most people know these things. Mobile phones are so advanced nowadays, there is nothing they don’t know.

Interviewer: It is also relatively easy to search for relevant knowledge on the Internet.

Interviewee: It seems like everyone knows about this. I can just buy a box of medicine from the pharmacy downstairs from my house and that’s it. It’s not that difficult. It’s not a big deal.

Interviewer: I understand. Do you sometimes chat with friends about this? Ask each other about how to prevent pregnancy.

Interviewee: I told Wang Dan that she had an IUD. Many people around her had IUDs, but I was afraid, so I didn’t wear one.

Interviewer: I have also learned about this and heard that some people have experienced side effects like bleeding, so you are still reluctant to take this approach?

Interviewee: Yes, that’s right.

Interviewer: I understand. Have you talked to a professional doctor or nurse about contraception?

Interviewee: No, we haven’t talked about it.

DOL_088:

Interviewer: I understand. I also understand that it is very hard for you to take care of children. Have you taken any contraceptive measures since you met your husband?

Respondent: Take.

Interviewer: What kind of contraceptive measures will you mainly take?

Interviewee: Do I need to tell you this? Can it be kept confidential?

Interviewer: Yes, if you feel uncomfortable, you don’t have to say it. What is the main reason for you to take contraceptive measures?

Respondent: Not yet.

Interviewer: Mainly worried about pregnancy.

Interviewer: Yes.

Interviewer: Do you take contraceptive measures because you are worried about the spread of sexually transmitted diseases?

Interviewee: I haven’t thought about that.

Interviewer: In your daily sex life, do you actively ask your husband to use condoms because you are worried about pregnancy?

Interviewee: Can we move on to the next topic?

Interviewer: Yes, this session is probably all about reproductive health. Do you think this topic is not easy to answer? Hello, do you think this question is not easy to answer?

Interviewee: Yes.

Interviewer: OK, I will ask all the questions in this session, but if you feel uncomfortable talking about it, you can tell me. Who is the main person who makes the decision when choosing different contraceptive measures?

Interviewee: That’s all we need to do to reach a consensus.

Interviewer: Are there any disagreements that usually arise?

Respondent: There is no disagreement.

Interviewer: Who will take the initiative to propose specific contraceptive measures?

Respondent: As long as everyone thinks it’s okay, that’s fine.

Interviewer: Have there ever been situations where, for example, you want him to take contraceptive measures, but he is reluctant to do so?

Interviewee: Not really.

Interviewer: I see. If you decide not to have children, do you always use contraception? Or sometimes you don't?

Respondent: Always.

Interviewer: Always, no matter what contraceptive measures you choose, where do you usually learn about contraception-related knowledge?

Interviewee: Check online.

Interviewer: Learn from the Internet. For example, do you ask experienced family members or friends for relevant information?

Respondent: No.

Interviewer: No, will you talk to professional doctors and nurses?

Interviewee: Not really.

Interviewer: I understand. Do you think the information you get from the Internet will be helpful for your contraceptive measures in real life?

Respondent: It’s okay at the moment.

DOL_089:

Interviewer: I see. Have you taken any contraceptive measures since you got married?

Respondent: I started taking contraceptive measures after giving birth.

Interviewer: What contraceptive measures are generally taken?

Interviewee: In rural areas, we use birth control and wear IUDs.

Interviewer: I understand. Now you get an IUD inserted after giving birth?

Interviewer: Yes.

Interviewer: I understand. What is the main reason for having an IUD inserted?

Respondent: I don’t want a second child. I’m afraid of having a second child.

Interviewer: I understand. Have you chosen contraceptive measures because you are worried about the spread of sexually transmitted diseases?

Interviewee: No, I’m just afraid of having children.

Interviewer: I understand. In addition to IUDs, before having IUDs, have you used any contraceptive measures such as condoms, birth control pills, or ejaculation outside the vagina, or safe period?

Respondent: Since we wanted to have children after we got married, we didn’t take any measures in this regard.

Interviewer: I understand. When you were pregnant and gave birth, who suggested having an IUD?

Interviewee: It is basically a natural phenomenon in our place. Women will get the IUDs on their own.

Interviewer: If you don’t want to have another baby, you will go.

Interviewer: Yes.

Interviewer: Did your husband have any different opinions from you on the issue of IUD insertion?

Respondent: No.

Interviewer: Where did you hear about the contraceptive method of IUD?

Interviewee: We all have IUDs in our place. It has been like this since my mother’s generation.

Interviewer: It seems that my experienced family members have also seen this happen, and most of my friends are probably the same.

Interviewee: Yes, it’s always like that.

Interviewer: Yes. Do you usually talk to your family members or friends who have experience about other contraceptive knowledge?

Respondent: No. Because all my friends are in the same situation.

Interviewer: I see. In general, in terms of contraceptive knowledge, do you have any other channels to learn about it?

Respondent: I haven’t paid much attention to this aspect.

Interviewer: When you needed to know something, did you search for relevant knowledge on the Internet?

Interviewee: After we got married, we wanted to have a child, so we didn't know about contraception. When the child was about one year old, we put on an IUD. During that period, before putting on the IUD, we also took some contraceptive measures.

Interviewer: What kind of contraceptive measures will be mainly taken at that stage?

Respondent: Condoms.

Interviewer: Mainly condoms.

Interviewee: Yes.

Interviewer: Did you use condoms because you were worried about getting pregnant?

Interviewer: Yes.

Interviewer: If condoms were used during the process, who would suggest it?

Interviewee: It was mainly me who brought it up.

Interviewer: Does your husband usually cooperate?

Respondent: Yes.

Interviewer: Were there any disagreements on this matter?

Respondent: No.

Interviewer: I understand. Apart from this, have you tried any other contraceptive methods during this period?

Respondent: No.

Interviewer: Yes. In terms of contraceptive knowledge, have you discussed with professional medical staff what to do?

Respondent: No.

DOL_090:

Interviewer: I see. What kind of contraceptive measures have you taken?

Respondent: Contraceptive measures, wearing condoms.

Interviewer: Have you ever considered things like the safe period, ejaculation outside the vagina, birth control pills, or IUDs?

Respondent: Yes.

Interviewer: Under what circumstances did the two unexpected pregnancies occur?

Respondent: I thought it was a safe period.

Interviewer: I see.

Respondent: I thought it was a safe period, but it is not a safe period, and the safe period is not safe either.

Interviewer: Yes. After your first unexpected pregnancy, did your views on these contraceptive measures change or which one you would consider taking?

Respondent: The best thing is sterilization.

Interviewer: Did you change your contraceptive method after giving birth to your second child? Or did you continue to use condoms?

Respondent: No.

Interviewer: Have you ever worried about an unexpected pregnancy?

Respondent: Worried.

Interviewer: Would you suggest, for example, more frequent use of condoms, or some other approach? Would you suggest it?

Respondent: I would like him to either get a vasectomy, male sterilization, take birth control pills, and have a safe period. Nothing is 100% effective, and in vitro fertilization is not 100% effective either. Condoms may have a higher chance of preventing pregnancy, but there is also the possibility of them breaking, or something like that. But the best method is to have no sperm.

Interviewer: I understand what you mean. When you communicate with your husband about contraceptive measures, do you have disagreements? For example, do you want to use condoms, but he doesn't want to.

Respondent: Maybe.

Interviewer: If this happens, what do you usually do?

Respondent: Just ejaculation outside the body.

Interviewer: Do you feel at ease with this?

Respondent: It will be better. The safe period is the least accurate.

Interviewer: I see.

DOL_091:

Interviewer: I see. Before you had children and planned to have a baby, how did you use contraception? For example, some common methods that people use more are condoms, safe period, ejaculation outside the body, or contraceptive pills, etc. Which one do you mainly use?

Interviewee: Condoms.

Interviewer: What is the frequency you use?

Respondent: How often do you use it?

Interviewer: Yes, for example, do you use it every time, or almost every time, or something like that.

Interviewee: I see what you mean. Yes, that's right.

Interviewer: You sounded quite cautious about this matter at the time. Were you worried about an unexpected pregnancy?

Interviewee: Yes.

Interviewer: What was your mood at that time? Why do you two think you were so cautious?

Interviewee: The problem is that I am not married yet.

Interviewer: I see. Have you ever had a disagreement with your husband or boyfriend on the use of condoms? For example, one party wanted to use it, but the other party didn't?

Interviewee: No.

Interviewer: I see. Where do you usually learn about contraception?

Interviewee: Do I need to know this?

Interviewer: Maybe you know more about it from the Internet or other channels, right?

Interviewee: Yes. You don’t need to know this.

Interviewer: That would be related to, for example, whether the parents have any.

Interviewee: I don’t discuss this issue with anyone. No one.

Interviewer: I see.

Interviewer: This is a rather obscure question, so even if you want to ask me I don’t want to reply to you.

Interviewer: I see. Do you talk to doctors and nurses about contraception?

Interviewer: Of course I will. It’s different from doctors in that I will answer whatever the doctor asks.

Interviewer: I see. Will you take the initiative to seek consultation from them?

Interviewee: No.

Interviewer: That is to say, we will only tell them the information when they need it and ask.

Interviewee: Yes, that’s right.

DOL_092:

Role 1: I understand. How have you been taking contraceptive measures? Commonly used methods may be condoms, safe period, birth control pills, or ejaculation outside the body. Which methods have you mainly used?

Role 2: Before having the second child, I basically ejaculated outside the body and used condoms. It didn't matter. After having the second child, I was determined not to have a third child, so I did what is called? Internal ejaculation.

Role 1: IUD.

Character 2: Yes.

Role 1: Intrauterine device.

Character 2: Yes.

Role 1: Understand. In the early stage, you may think that ejaculation outside the vagina or using condoms is safer, but after experiencing it once, you still think you have to be extremely safe.

Character 2: Yes, it is.

Character 1: Yes, indeed. When you used condoms and ejaculation outside the body, how often did you use condoms?

Role 2: Should it be, frequency?

Role 1: Yes, you mean to use it every time.

Role 2: No

Character 1: General?

Role 2: Generally not used, but sometimes used.

Character 1: I see. When you are not using it, are you more worried about unwanted pregnancy or the spread of diseases?

Role 2: Still worried.

Character 1: Are you worried every time?

Role 2: I’m not worried every time, but I’m pretty worried most of the time. That’s the thought.

Role 1: Would you communicate with your husband about your concerns?

Character 2: Yes, I didn’t say it at first, but I still said it later.

Role 1: Why don’t you tell me at the beginning why you didn’t communicate with him?

Role 2: Why don't you communicate? I didn't have such a strong idea at first. I thought I was just too worried. It was just my own idea. We usually pay attention to personal hygiene, so I was just worried on the surface. I didn't feel worried in my heart. But later I felt that I couldn't do that because I still had gynecological inflammation after giving birth. I went to see a doctor, and then I felt very worried. From then on, I might use condoms more.

Role 1: Maybe it’s a combination of two things. On the one hand, I’m worried about an unexpected pregnancy, but on the other hand, I also think that this might be healthier.

Character 2: Yes, it is.

Role 1: Sometimes you try to communicate with him, for example, when it comes to using condoms, do you think he is more supportive of your idea, or do you sometimes have different opinions?

Character 2: Not quite the same, he just doesn’t like using that thing.

Role 1: I see. What should I do now?

Role 2: It is ejaculation outside the body.

Character 1: So do you feel like you've compromised? If you resort to having sex outside, do you think you're completely okay with that, or do you still feel like you've given in a little?

Role 2: Maybe I gave in and didn't fully accept it. I think that's also problematic. I think the probability of pregnancy is still there.

Character 1: Yes, that will lead you to some comparisons.

Character 2: Not really.

Character 1: There are no other emotions, right?

Character 2: Not really.

Role 1: Where do you usually learn about contraception?

Role 2: Where did you learn about it? I really can't remember, it was a long time ago. I went to the hospital to spread it. What was the reason? Yes, I went to the hospital for a checkup for gynecological inflammation. The doctor asked me what method I used, and then I think the doctor's method spread it more.

Role 1: I see. Yes, that’s what the doctor told you when you were seeing the doctor. It sounds like you rarely talk about this with your family or friends, right?

Character 2: Yes.

Role 1: Would you find out from the Internet?

Role 2: Yes, you can find it on the Internet. Just search on Baidu.

Role 1: I understand.

Character 2: There should be.

DOL_093:

Character 1: I see. How have you been using contraception?

Role 2: Condom.

Role 1: Sometimes you may consider other measures such as safe period, ejaculation outside the body, birth control pills, or intrauterine devices.

Role 2: I haven't considered an IUD, because many of the female friends we know sometimes have back pain or stomach pain. I have never done sterilization.

Role 1: Do you sometimes not wear condoms? How often do you wear condoms? How often do you think that is?

Role 2: 90%, he did a pretty good job in this aspect, because he is a fat guy, and I am a thin, skinny person. I have such a poor physique, I said don't accidentally have to have surgery again, it will hurt my body more.

Character 1: Okay. I just said that it sounds like your second pregnancy was an accident.

Character 2: I haven't had any since.

Role 1: Under what circumstances was the second pregnancy an accidental one?

Role 2: I should say I wore a condom at that time, but maybe it fell off in the middle or something, I have no idea what happened.

Character 1: Yes, so you didn’t expect that, right?

Character 2: Yeah, no idea, no plan.

Role 1: I see. When you first got married, you wanted to have children naturally, so you didn't take any contraceptive measures, right?

Character 2: Right.

Role 1: I understand. Do you and your husband have different opinions on contraceptive measures? Are there times when one of you wants to use it but the other doesn't?

Role 2: He respects me in this respect.

Character 1: So he tends to listen to your opinions, right?

Role 2: Yes, (inaudible 00:55:41) It’s a bit long. He still respects me in this respect. He does a good job in this respect. But now I don’t know whether I should say it or not. I’d rather not say anything. Now we have very few.

Role 1: I understand.

Role 2: His physical condition was getting worse and worse, he was getting fatter and fatter, and coupled with the fact that they were often separated from each other, he had very little time to spare.

Role 1: I understand. From what aspects do you generally learn about contraception?

Character 2: Your what? Your voice is a little low.

Role 1: Okay, let me ask you, how did you learn about contraception?

Role 2: From the Internet, when I was learning, there was also a small part of it in biology class when I was in school.

Role 1: Yes. Do you talk to your family or friends about contraception? Do they share some useful knowledge with you?

Role 2: I have talked to my friends about it and learned about the intrauterine devices they use. I have talked to my sister about it, but no one else has mentioned it.

Role 1: I see. Do you talk to doctors and nurses about these things?

Role 2: We didn’t really talk about it.

Role 1: I see. You just mentioned that you would use condoms in 90% of cases. What is the situation like in the other 10%?

Role 2: Because some people may not wear it at the beginning, and sometimes they only wear it in the middle.

Character 1: I didn’t wear it in the early stage, but only wore it in the middle, is that right?

Character 2: Yes.

Role 1: Do you have any concerns during this process? Are you worried about accidentally getting pregnant?

Role 2: I would also be a little worried.

Role 1: Would you tell him about your concerns?

Character 2: What did you say?

Role 1: Will you tell him about your concerns?

Role 2: I can speak, I definitely can speak.

Role 1: Was it because you strongly requested that he put on the condom, or did he do it on his own initiative?

Role 2: He will take the initiative on his own, and I don’t ask him to do it too strongly.

DOL_094:

Character 1: Okay. How many children do you have now?

Role 2: Two children.

Role 1: Under what circumstances did you decide to have a child?

Role 2: Just let it go.

Role 1: How long after you got married did you have your first child?

Role 2: Two or three years.

Character 1: So this is also part of the plan, right?

Character 2: Yes, just let it be.

Role 1: Understand, how have you usually used contraception since you got together? Commonly used methods include condoms, safe period, withdrawal, or contraceptive pills?

Role 2: Condom.

Role 1: Before you tried to conceive, how often did you use condoms? For example, every time, or about 50% or 80%.

Role 2: Use it every time.

Character 1: How did you decide to take this contraceptive measure?

Role 2: Because miscarriage is too harmful to the body.

Character 1: You are still worried about the chance of pregnancy, right?

Character 2: Yes.

Role 1: Have you ever had a disagreement with your husband over condom use? For example, were there times when you didn't want to use it but he did, or when he didn't want to use it but you did?

Character 2: No.

Character 1: It sounds like we're pretty much in agreement on this, right?

Character 2: Yes.

Role 1: Why do you think the two people are so highly consistent on this matter?

Role 2: The body is definitely the most important.

Character 1: Indeed, who decided at the beginning that we could mainly adopt this approach?

Role 2: My husband.

Role 1: I understand. Sometimes you may have concerns, for example, about whether contraceptive measures are safe?

Character 2: Not yet.

Role 1: I see. Will you talk to your family about how to use contraception?

Role 2: My mother asked and I told her about it.

Role 1: Do you think she can help you?

Role 2: Yes.

Character 1: Can you elaborate on this?

Character 2: What help do you mean? Which help?

Role 1: For example, you think your mother, for example, the information she gave you, you think it is useful, or sometimes when you want to find someone to ask, because everyone certainly didn’t know these things when they were young, do you think she can help you with these things, this information, this knowledge?

Role 2: My mother usually doesn't ask me about these things, except after I got married when she had time. For example, right after giving birth, she would tell you that she is afraid of getting pregnant because she is worried that it will be bad for your health. That's it.

Role 1: I see. Do you talk to your friends about contraception?

Character 2: No.

Character 1: Why?

Role 2: Because we don’t normally talk about these topics.

Role 1: I still feel a little awkward and embarrassed.

Character 2: Yeah. I don’t know how to talk about this.

Role 1: I see. Would you talk to your doctor or nurse about this?

Role 2: We didn’t talk about this.

Role 1: I understand. How do you usually learn about contraception?

Role 2: Mobile phone.

Role 1: Is it the Internet?

Character 2: Yes.
